# Phase I Randomized Clinical Trial of VRC DNA and rAd5 HIV-1 Vaccine Delivery by Intramuscular (IM), Subcutaneous (SC) and Intradermal (ID) Administration (VRC 011)

**DOI:** 10.1371/journal.pone.0091366

**Published:** 2014-03-12

**Authors:** Mary E. Enama, Julie E. Ledgerwood, Laura Novik, Martha C. Nason, Ingelise J. Gordon, LaSonji Holman, Robert T. Bailer, Mario Roederer, Richard A. Koup, John R. Mascola, Gary J. Nabel, Barney S. Graham, the VRC 011 Study Team

**Affiliations:** 1 Vaccine Research Center, National Institute of Allergy and Infectious Diseases, National Institutes of Health, Bethesda, Maryland, United States of America; 2 Biostatistics Research Branch, Division of Clinical Research, National Institute of Allergy and Infectious Diseases, National Institutes of Health, Bethesda, Maryland, United States of America; University of Alabama, United States of America

## Abstract

**Background:**

Phase 1 evaluation of the VRC HIV DNA and rAd5 vaccines delivered intramuscularly (IM) supported proceeding to a Phase 2 b efficacy study. Here we report comparison of the IM, subcutaneous (SC) and intradermal (ID) routes of administration.

**Methods:**

Sixty subjects were randomized to 6 schedules to evaluate the IM, SC or ID route for prime injections. Three schedules included DNA primes (Wks 0,4,8) and 3 schedules included rAd5 prime (Wk0); all included rAd5 IM boost (Wk24). DNA vaccine dosage was 4 mg IM or SC, but 0.4 mg ID, while all rAd5 vaccinations were 10^10^ PU. All injections were administered by needle and syringe.

**Results:**

Overall, 27/30 subjects completed 3 DNA primes; 30/30 subjects completed rAd5 primes. Mild local pruritus (itchiness), superficial skin lesions and injection site nodules were associated with ID and SC, but not IM injections. All routes induced T-cell and antibody immune responses after rAd5 boosting. Overall, >95% had Env antibody and >80% had Env T-cell responses.

**Conclusions:**

The pattern of local reactogenicity following ID and SC injections differed from IM injections but all routes were well-tolerated. There was no evidence of an immunogenicity advantage following SC or ID delivery, supporting IM delivery as the preferred route of administration.

**Trial Registration:**

Clinicaltrials.gov NCT00321061

## Introduction

Route of administration can influence the safety and immunogenicity profile and dosage requirements of a vaccine regimen, and each vaccine in use for prevention of human disease has a preferred route of administration. Choosing a preferred vaccine delivery route should take into consideration such factors as achieving a protective immune response, cost per dose, the ease of storage, transport and administration, manufacturing efficiency and stability, and safety for both the administrator and recipient. Injected vaccines are commonly administered intramuscularly (IM), with examples being influenza trivalent inactivated vaccine, inactivated polio, pneumococcal, hepatitis B and others. Other injection routes include subcutaneous (SC), such as for MMR (measles, mumps, rubella), varicella, meningococcal polysaccharide, and intradermal (ID), such as was used for the Dryvax smallpox vaccine in the past and more recently for an inactivated influenza vaccine.

To put this study into historic perspective, prior to the initiation of protocol VRC 011 to compare the IM, SC and ID routes of administration, the NIAID Vaccine Research Center (VRC) had clinical trial data indicating that HIV-1 DNA and recombinant adenoviral vector serotype 5 (rAd5) vaccines showed promising cellular and humoral immunogenicity, and plans for further evaluation as preventive HIV vaccine strategy in a prime-boost regimen were in progress [1]–[4]. It has since been determined through the HVTN 505 clinical trial that this DNA prime-rAd5 boost regimen is not effective in prevention of HIV [5]. Nonetheless, given that there are few randomized vaccine studies specifically designed to compare routes of administration, we are reporting this clinical trial to add to this knowledge base, and to contribute to the public record on one of the few vaccine regimens to be tested in an efficacy trial for prevention of HIV.

In the efficacy study of the prime-boost regimen, the DNA vaccine, VRC-HIVDNA016-00-VP, was administered IM in a 3-injection schedule at the 4 mg dosage by Biojector and the recombinant adenoviral vector serotype 5 (rAd5) vaccine, VRC-HIVADV014-00-VP, was administered IM as a single booster injection at the 10^10^ particle unit (PU) dosage by needle injection [5]. At the same time that plans were proceeding to develop a large efficacy study of these HIV vaccines in a prime-boost schedule, the VRC 011 study was designed to evaluate alternative routes of administration, and their relative safety and immunogenicity. It is important to note that at that time a large efficacy study, known as the Step Study, with repetitive dosing of a different adenoviral vector vaccine (Merck rAd5) [6] was also underway and the outcome affected other studies with rAd5 vaccines.

VRC 011 was designed to evaluate routes of administration for priming injections and was prospectively focused on T cell responses to EnvA and antibody responses to EnvC based on the earliest studies with the DNA and rAd5 vaccines [1]–[4]. In parallel the HIV Vaccine Trials Network (HVTN) performed a study, HVTN 069, to evaluate alternative routes of administration for the VRC rAd5 booster injection [7].

The DNA vaccine had been given primarily IM by Biojector, which is a needle-free delivery device that produces a cone-shaped distribution of injectate with the majority of vaccine deposited in muscle, but some portion also deposited in skin and subcutis. Biologically, vaccine deposited in the skin or subcutaneous tissue may induce a different pattern of immune responses than vaccine deposited in muscle and may affect the functional properties of the immune response, including the pattern of cytokine production by lymphocytes [8]. Langerhans cells are the primary antigen presenting cell (APC) in the skin [9], [10] and antigen presentation exclusively by Langerhans cells may be more efficient in antigen presentation than other dendritic cell (DC) subpopulations, perhaps requiring a smaller quantity of antigen to become activated and migrate to regional lymph nodes where adaptive immune responses can be initiated [11], [12]. In order to better control and observe the effect of depositing the vaccine in a particular layer of tissue, only needle injection was used in this study.

The highest concentration of the DNA vaccine available was a 4 mg/mL formulation. This allowed the usual 4 mg dosage to be administered for IM and SC injection, but the ID route was limited to a 0.1 mL administration volume and therefore a dosage of 0.4 mg. The rAd5 vaccine was available as both a 10^10^ PU/mL and 10^11^ PU/mL formulation and by all routes a 10^10^ PU dosage could be administered by using 1 mL of the former for IM and SC injection and 0.1 mL of the latter for the ID injections.

## Methods

The protocol for this trial and supporting CONSORT checklist are available as supporting information; see Protocol S1 and Checklist S1.

### Ethics Statement

This study was approved by the National Institute of Allergy and Infectious Diseases Institutional Review Board, and was performed in accordance with 45 CFR Part 46, U.S. Food and Drug Administration regulations for investigational products, and principles expressed in the Declaration of Helsinki. All subjects completed the consent process and signed written informed consent documents. The authors confirm that the clinical trials for the recruitment and screening of study volunteers and for the administration of the study agents reported here are registered.

### Study Design

VRC 011 (NIH 06-I-0149, NCT00321061) was conducted at the National Institutes of Health (NIH), Bethesda, MD by the Vaccine Research Center (VRC). It was a Phase I, randomized study in healthy adults, 18–50 years old. Recruitment and screening of volunteers was through an IRB-approved screening protocol (NIH 02-I-0127, NCT00031304) with informed consent to be screened for an HIV vaccine clinical trial.

The primary objectives were to examine safety and tolerability of the vaccines by three routes of administration in regimens that included either three prime vaccinations with the DNA vaccine at Weeks 0, 4 and 8 followed by one rAd5 vaccination booster at Week 24 (Group 1) or one prime rAd5 vaccine at Week 0 followed by one rAd5 booster at Week 24 (Group 2). The secondary objectives related to immunogenicity included the following: 1) a difference in ELISpot responses to EnvA of 3-fold or greater among those receiving SC when compared to IM 4 weeks after the end of the prime regimen among the groups who received DNA prime; 2) a difference in ELISA responses to EnvC of 5-fold or greater SC when compared to IM 4 weeks after the end of the prime regimen among the groups who received DNA prime; 3) a response rate of at least 50% in EnvA ELISpot or EnvC ELISA among those receiving ID DNA prime at 4 weeks after the prime injection(s); 4) a difference in ELISpot responses to EnvA of 3-fold or greater among those receiving SC when compared to IM 4 weeks after the end of the prime regimen among the groups who received rAd5 prime; and 5) a difference in ELISA responses to EnvC of 5-fold or greater SC when compared to IM 4 weeks after the end of the prime regimen among the groups who received rAd5 prime. Exploratory analyses included comparing the six priming schedules with respect to ELISA, ELISpot, and ICS responses at 4 weeks after the boost.

A total of 60 subjects were randomized to these two groups of 30 subjects in a 3×2 factorial design. Within each group, subjects were randomized in a 1∶1∶1 ratio to receive the prime vaccinations by the IM, SC or ID routes. The randomization plan was developed by the protocol statistician using computer-generated random numbers and ensured that each of 6 randomization schedules was comprised of 10 subjects total, including 5 subjects with negative (<1∶12) and 5 with positive (≥1∶12) screening adenovirus serotype 5 antibody (Ad5Ab) titers. When administered IM or SC, the DNA vaccine dosage was 4 mg. When administered ID, the DNA vaccine dosage was 0.4 mg. The dosage of all rAd5 vaccine injections was 10^10^ PU by all three routes of injection. All rAd5 booster injections were IM, regardless of the priming injection(s). All injections were administered by needle and syringe. The vaccination schedule assignment became known to both clinicians and subjects after completion of an electronic enrollment in the study database. The study was fully accrued and prime injections administered in accordance with the original study design. A significant change in plan that occurred during the conduct of the study was the decision to not administer rAd5 vaccine boosts to subjects not yet boosted, who had enrolled with positive screening Ad5Ab titers after the results of the Step Study were taken into consideration [6].

Clinical safety evaluations included laboratory tests, physical assessments, 5-day reactogenicity subject assessments and clinician assessment of the injection site at Day 3±1 day after the priming injection(s). Clinic visits through study week 42 and a long-term follow-up contact at Study Week 94 were required. The blood samples collected 4 weeks after prime injection(s) were prospectively planned for the route of administration immunogenicity assessment and other timepoints for exploratory immunogenicity.

### Vaccines

The DNA vaccine, VRC-HIVDNA016-00-VP [3], and rAd5 vaccine, VRC-HIVADV014-00-VP [2], [13], have been described elsewhere used alone and in combination [4], [14]–[17]. Briefly, the DNA vaccine is composed of 6 closed, circular DNA plasmids that encode HIV-1 Gag, Pol and Nef (from clade B) and Env glycoprotein from clade A, clade B, and clade C; each plasmid comprises 16.67% (by weight). The rAd5 vaccine is composed of 4 recombinant replication-defective adenovirus serotype 5 vectors that encode for HIV-1 Gag/Pol polyproteins (from clade B) and Env glycoproteins from clades A, B, and C, combined in a 3∶1∶1∶1 ratio, respectively. Vaccines were tested in compliance with good manufacturing practices before release for use in clinical trials.

### Immunology Assay Methods

The laboratory assay methods used in this study have been previously published [1], [2], [4]. The primary immunogenicity endpoints were defined prospectively as cellular immune responses to the EnvA antigen and antibody responses to the EnvC antigen, based on earlier studies in which these antigens were associated with greater magnitude of T cell and antibody responses, respectively [4]. The prospectively defined endpoints to consider if SC or ID might offer benefit over the IM route were: 1) EnvA ELISpot responses of 3-fold or greater after SC injections compared to IM at 4 weeks after DNA prime regimens or in SC or ID injections compared to IM at 4 weeks after rAd5 prime regimens; 2) response rate of at least 50% in EnvA ELISpot or EnvC ELISA at 4 weeks after DNA ID prime injections; 3) ELISA responses to EnvC of 5-fold or greater after SC injections compared to IM at 4 weeks after DNA prime or rAd5 prime regimens; and 6) comparison of the six priming schedules with respect to EnvC ELISA, and EnvA ELISpot, and ICS responses at 4 weeks after the rAd5 boost.

### Statistical Methods

All available data were used, according to randomization assignment. The analyses for the primary objectives of safety and tolerability are solely descriptive, with percentages and (where applicable) exact confidence intervals reported. For the secondary analyses for immunogenicity, we used assay-specific pre-defined positivity criteria to categorize each individual as a responder or a non-responder at each time point. For ELISpot, a response was defined to be peptide-stimulated number of spots per million of at least 59 and >4-fold above background. For ICS, a positive response was defined to be one where the proportion of positive cells was statistically higher in the peptide-stimulated condition as compared to the background-stimulated condition by a one-sided Fisher's exact test, and the difference in the percentages was at least.045%. For ELISA, a positive response was defined as any titer of 30 or greater.

Analyses of immunological data included univariate summaries (such as confidence intervals) as well as comparisons between the randomized groups. Between-group comparisons use Fisher's Exact Test for binary data (such as response/non-response) and Wilcoxon rank sum tests for the magnitude of the response. Linear and logistic regressions were used to examine the response rates and magnitudes for effect of group across route, and to look for an effect of pre-existing Ad5 titer. Since these were secondary analyses, there was not adjustment for multiple comparisons between groups or between assays, but instead required a p-value threshold of .01 for statistical significance. P-values between .01 and .05 are noted as suggestive.

## Results

### Subject Population

Recruitment and screening of 191 volunteers occurred from September 13, 2005 through October 1, 2007. Sixty eligible and willing subjects were enrolled into the vaccine study between May 16, 2006 and Oct 15, 2007. Study vaccinations were administered from May 16, 2006 through March 31, 2008 and the final long term follow-up visit was completed August 17, 2009. Demographics for the vaccine study participants are shown in Table 1 by group and overall.

**Table 1:** VRC 011 Baseline Characteristics of Participants.

Category	Characteristic	DNA Prime Group (n = 30)	rAd5 Prime Group (n = 30)	Overall (n = 60)
Gender - no. (%)	Male	15 (50)	19 (63)	34 (57)
	Female	15 (50)	11 (37)	26 (43)
Race - no. (%)	White	21 (70)	24 (80)	45 (75)
	Black or African American	7 (23)	4 (13)	11 (18)
	All other races combined	2 (7)	2 (7)	4 (7)
Ethnicity - no. (%)	Non-Hispanic/Latino	28 (93)	29 (97)	57 (95)
	Hispanic/Latino	2 (7)	1 (3)	3 (5)
Age - mean [std. dev.]	Mean years [S.D.]	29.2 [8.5]	29.4 [8.5]	29.3 [8.5]
BMI - mean [std. dev.]	Mean [S.D.]	24.3 [4.3]	25.2 [3.5]	24.7 [3.9]

### Vaccination and study completion

**Figure 1:**
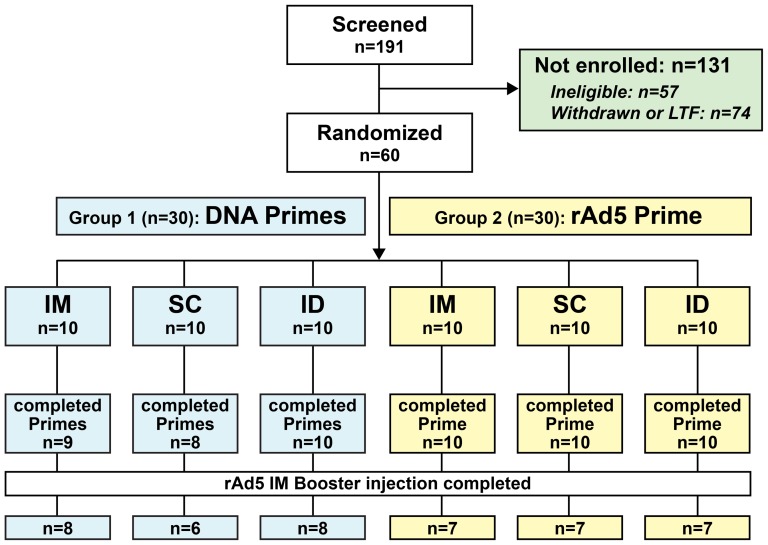
VRC 011 Disposition Flow Diagram of Screening, Randomization and Vaccination Completion.

The disposition of subjects with regard to vaccinations is shown for the 6 schedules in the CONSORT diagram (**Figure 1**). All 60 subjects started study injections. In Group 1, 27/30 completed 3 DNA primes and 22/30 completed the boost. In Group 2, 30/30 completed the rAd5 prime and 21/30 completed the boost. Reasons for discontinuations from vaccination were: lost to follow-up (n = 1), protocol noncompliance (n = 1), withdrawal (n = 4), adverse events (n = 4), discontinuations in consideration of the Step Study results with a different adenoviral vector vaccine (MRK-Ad5) in subjects who were Ad5 seropositive at screening or assessed as not meeting criteria for low risk for HIV exposure (n = 7). Overall, 54/60 completed clinic visits through Week 42 and 53/60 completed the long-term follow-up contact.

### Safety

There were no serious adverse events. Adverse events after which vaccinations were discontinued in Group 1 included: palate cyst (grade 2) assessed as unrelated to DNA IM prime and intermittent generalized urticaria (grade 3) 25–29 days after DNA ID prime assessed as probably not related and in Group 2 included: urticaria (grade 1) starting 12 days after rAd5 IM prime assessed as possibly related and Grade 2 erythema (10×8 cm at maximum) at the injection site 3–5 days after rAd5 SC prime injection assessed as definitely related.

The frequency of solicited local and systemic reactogenicity parameters is shown in Table 2 and Table 3, respectively. The solicited local parameters of redness and swelling varied by route of administration (p<.001 for both) with greater frequency by either SC or ID routes compared to IM. The unsolicited local reactions varied by route of administration and these are provided as a line listing in Table 4. In the DNA prime group unsolicited local reactions included 1 subject with an injection site nodule after 3^rd^ DNA ID and 3 subjects with mild injection site pruritus (without rash) after 3^rd^ DNA ID. In the rAd5 prime group unsolicited local reactions included 5 subjects with mild superficial injection site lesions (without pustules or vesicles) after rAd5 ID prime; 5 subjects (2 rAd5 SC and 3 rAd5 ID) with an injection site nodule, and 1 subject with an injection site papule after rAd5 IM prime. These healed without need for treatment. Eight subjects (6 rAd5 ID and 2 rAd5 SC) had mild injection site pruritus (without rash). The rate of pruritus and local injection site lesions varied by route (p<.001) with greater frequency by either the SC or ID routes compared to IM.

**Table 2:** Maximum Local Reactogenicity Summary for All Vaccinations.

	DNA IM Prime (N = 10)	DNA SC Prime (N = 10)	DNA ID Prime (N = 10)	rAd IM Prime (N = 10)	rAd SC Prime (N = 10)	rAd ID Prime (N = 10)	rAd IM Boost (N = 43)
	number (%)						
PAIN/TENDERNESS							
None	4 (40)	2 (20)	6 (60)	3 (30)	1 (10)	5 (50)	9 (20.9)
Mild	5 (50)	8 (80)	4 (40)	7 (70)	9 (90)	5 (50)	32 (74.4)
Moderate	0 (0)	0 (0)	0 (0)	0 (0)	0 (0)	0 (0)	2 (4.7)
Severe	0 (0)	0 (0)	0 (0)	0 (0)	0 (0)	0 (0)	0 (0)
Missing	1 (10)	0 (0)	0 (0)	0 (0)	0 (0)	0 (0)	0 (0)
SWELLING							
None	10 (100)	7 (70)	1 (10)	9 (90)	3 (30)	1 (10)	39 (90.7)
Mild	0 (0)	3 (30)	9 (90)	1 (10)	7 (70)	9 (90)	4 (9.3)
Moderate	0 (0)	0 (0)	0 (0)	0 (0)	0 (0)	0 (0)	0 (0)
Severe	0 (0)	0 (0)	0 (0)	0 (0)	0 (0)	0 (0)	0 (0)
Missing	0 (0)	0 (0)	0 (0)	0 (0)	0 (0)	0 (0)	0 (0)
REDNESS							
None	9 (90)	6 (60)	0 (0)	9 (90)	2 (20)	1 (10)	39 (90.7)
Mild	1 (10)	4 (40)	10 (100)	1 (10)	7 (70)	9 (90)	4 (9.3)
Moderate	0 (0)	0 (0)	0 (0)	0 (0)	1 (10)	0 (0)	0 (0)
Severe	0 (0)	0 (0)	0 (0)	0 (0)	0 (0)	0 (0)	0 (0)
Missing	0 (0)	0 (0)	0 (0)	0 (0)	0 (0)	0 (0)	0 (0)
ANY LOCAL SYMPTOM							
None	5 (50)	1 (10)	0 (0)	3 (30)	1 (10)	1 (10)	9 (20.9)
Mild	5 (50)	9 (90)	10 (100)	7 (70)	8 (80)	9 (90)	32 (74.4)
Moderate	0 (0)	0 (0)	0 (0)	0 (0)	1 (10)	0 (0)	2 (4.7)
Severe	0 (0)	0 (0)	0 (0)	0 (0)	0 (0)	0 (0)	0 (0)
Missing	0 (0)	0 (0)	0 (0)	0 (0)	0 (0)	0 (0)	0 (0)

**Table 3:** Maximum Systemic Reactogenicity Summary for All Vaccinations.

	DNA IM Prime (N = 10)	DNA SC Prime (N = 10)	DNA ID Prime (N = 10)	rAd IM Prime (N = 10)	rAd SC Prime (N = 10)	rAd ID Prime (N = 10)	rAd IM Boost (N = 43)
	number (%)						
MALAISE							
None	5 (50)	7 (70)	5 (50)	9 (90)	6 (60)	6 (60)	25 (58.1)
Mild	3 (30)	3 (30)	5 (50)	1 (10)	4 (40)	3 (30)	14 (32.6)
Moderate	1 (10)	0 (0)	0 (0)	0 (0)	0 (0)	0 (0)	3 (7)
Severe	0 (0)	0 (0)	0 (0)	0 (0)	0 (0)	0 (0)	0 (0)
Missing	1 (10)	0 (0)	0 (0)	0 (0)	0 (0)	1 (10)	1 (2.3)
MYALGIA							
None	7 (70)	9 (90)	8 (80)	8 (80)	6 (60)	6 (60)	27 (62.8)
Mild	2 (20)	1 (10)	2 (20)	2 (20)	4 (40)	3 (30)	11 (25.6)
Moderate	0 (0)	0 (0)	0 (0)	0 (0)	0 (0)	0 (0)	4 (9.3)
Severe	0 (0)	0 (0)	0 (0)	0 (0)	0 (0)	0 (0)	0 (0)
Missing	1 (10)	0 (0)	0 (0)	0 (0)	0 (0)	1 (10)	1 (2.3)
HEADACHE							
None	5 (50)	7 (70)	9 (90)	8 (80)	4 (40)	6 (60)	25 (58.1)
Mild	4 (40)	2 (20)	1 (10)	2 (20)	5 (50)	3 (30)	16 (37.2)
Moderate	0 (0)	1 (10)	0 (0)	0 (0)	1 (10)	0 (0)	1 (2.3)
Severe	0 (0)	0 (0)	0 (0)	0 (0)	0 (0)	0 (0)	0 (0)
Missing	1 (10)	0 (0)	0 (0)	0 (0)	0 (0)	1 (10)	1 (2.3)
CHILLS							
None	9 (90)	10 (100)	10 (100)	10 (100)	8 (80)	8 (80)	35 (81.4)
Mild	0 (0)	0 (0)	0 (0)	0 (0)	2 (20)	1 (10)	4 (9.3)
Moderate	0 (0)	0 (0)	0 (0)	0 (0)	0 (0)	0 (0)	3 (7)
Severe	0 (0)	0 (0)	0 (0)	0 (0)	0 (0)	0 (0)	0 (0)
Missing	1 (10)	0 (0)	0 (0)	0 (0)	0 (0)	1 (10)	1 (2.3)
NAUSEA							
None	9 (90)	9 (90)	10 (100)	10 (100)	9 (90)	9 (90)	38 (88.4)
Mild	0 (0)	1 (10)	0 (0)	0 (0)	1 (10)	0 (0)	4 (9.3)
Moderate	0 (0)	0 (0)	0 (0)	0 (0)	0 (0)	0 (0)	0 (0)
Severe	0 (0)	0 (0)	0 (0)	0 (0)	0 (0)	0 (0)	0 (0)
Missing	1 (10)	0 (0)	0 (0)	0 (0)	0 (0)	1 (10)	1 (2.3)
TEMPERATURE							
None	8 (80)	10 (100)	10 (100)	10 (100)	9 (90)	9 (90)	36 (83.7)
Mild	1 (10)	0 (0)	0 (0)	0 (0)	1 (10)	0 (0)	5 (11.6)
Moderate	0 (0)	0 (0)	0 (0)	0 (0)	0 (0)	0 (0)	1 (2.3)
Severe	0 (0)	0 (0)	0 (0)	0 (0)	0 (0)	0 (0)	0 (0)
Missing	1 (10)	0 (0)	0 (0)	0 (0)	0 (0)	1 (10)	1 (2.3)
ANY SYSTEMIC SYMPTOM							
None	3 (30)	6 (60)	5 (50)	7 (70)	3 (30)	4 (40)	18 (41.9)
Mild	5 (50)	3 (30)	5 (50)	3 (30)	6 (60)	5 (50)	19 (44.2)
Moderate	1 (10)	1 (10)	0 (0)	0 (0)	1 (10)	0 (0)	5 (11.6)
Severe	0 (0)	0 (0)	0 (0)	0 (0)	0 (0)	0 (0)	0 (0)
Missing	1 (10)	0 (0)	0 (0)	0 (0)	0 (0)	1 (10)	1 (2.3)

**Table 4:** Listing of Unsolicited Local Reactions After Prime Injections.

Prime Route	Reaction Type	Onset Day	Duration (days)
**DNA ID**	**abrasion**	**2**	**12**
**DNA ID**	**nodule**	**2**	**16**
**rAd5 SC**	**nodule**	**14**	**3**
**rAd5 ID**	**nodule**	**1**	**4**
**rAd5 SC**	**nodule**	**7**	**7**
**rAd5 ID**	**nodule**	**28**	**14**
**rAd5 ID**	**nodule**	**14**	**28**
**rAd5 ID**	**erosion/scab**	**8**	**9**
**rAd5 ID**	**erosion/scab**	**6**	**16**
**rAd5 ID**	**erosion/scab**	**10**	**14**
**rAd5 ID**	**erosion/scab**	**14**	**2**
**rAd5 ID**	**erosion/scab**	**8**	**7**
**rAd5 IM**	**papule**	**1**	**4**
**DNA ID**	**pruritus**	**0**	**8**
**DNA ID**	**pruritus**	**2**	**1**
**DNA ID**	**pruritus**	**2**	**7**
**rAd5 ID**	**pruritus**	**0**	**5**
**rAd5 ID**	**pruritus**	**0**	**6**
**rAd5 ID**	**pruritus**	**3 and 11**	**1 each**
**rAd5 ID**	**pruritus**	**2**	**1**
**rAd5 ID**	**pruritus**	**0**	**1**
**rAd5 ID**	**pruritus**	**2**	**1**
**rAd5 SC**	**pruritus**	**2**	**2**
**rAd5 SC**	**pruritus**	**9**	**1**

### Vaccine-induced T cell responses

**Figure 2:**
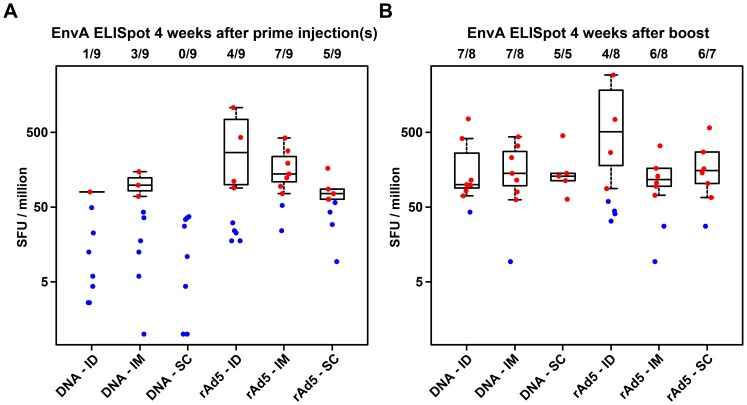
ELISpot responses among the different groups after priming with vaccine and route indicated on X-axis (panel A) and after rAd5 boosting IM (panel B). The numbers above each boxplot represent the fraction of participants in each group with available data at that time point who were judged to be responders using predefined criteria. The responders are represented on the plot with red dots, and are used to construct the boxplots; blue points represent non-responders and are not included in the boxplots.

**Figure 3:**
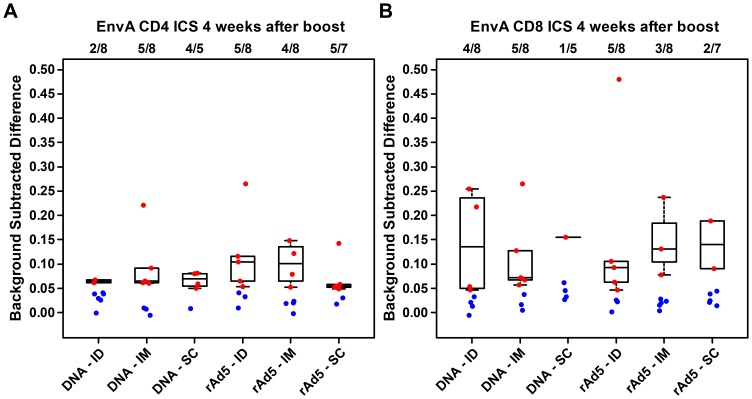
ICS responses among the different priming groups after boosting with rAd5 IM for CD4 T cells (A) and CD8 T cells (B). The numbers above each boxplot represent the fraction of participants in each group with available data at that time point who were judged to be responders using predefined positivitycriteria. The responders are represented on the plot with red dots, and are used to construct the boxplots; blue points represent non-responders and are not included in the boxplots.

The highest ELISpot response rates (87.5% to 100%) were in groups primed by DNA and boosted with rAd5, but there was no difference between routes of administration. The difference in median EnvA-specific ELISpot response magnitude at 4 weeks after priming injections was not statistically significant (**Figure 2A**). As there was no suggestion that either ID or SC prime regimens produced better responses than the IM prime regimen, no further comparisons were done. For DNA primed groups, the mean magnitude of ELISpot responses for EnvA at the timepoint 4 weeks post boost among the responders was similar, in the range of 100–140 for all three regimens, while for rAd5 prime groups, median responses were 509 in the ID group, 116 in the IM group, and 152 in the SC group (**Figure 2B**). Although the observed median magnitude in the ID group meets the predefined threshold of 3-fold higher than the median response among those who responded in the IM group, this result must be interpreted carefully, because the number of responders included in each group differs, and there were fewer responders in the group with the higher median response. In addition, the comparison of response magnitude among responders is not significant either within regimen or when combined across regimens. The ICS data evaluating CD4 and CD8 T cell cytokine production did not show significant differences in frequency or magnitude between groups (**Figures 3A and 3B**).

### Vaccine-specific antibody responses

**Figure 4:**
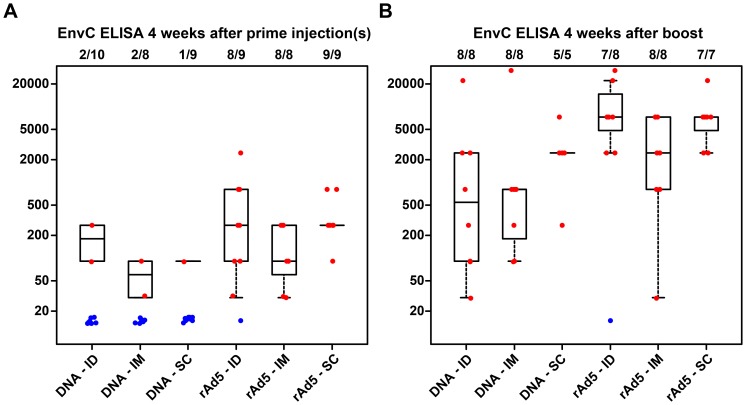
ELISA responses among the different groups after priming by route and vaccine indicated on X-axis (A) and boosting with rAd5 IM (B). The numbers above each boxplot represent the fraction of participants in each group with available data at that time point who were judged to be responders using predefined criteria. The responders are represented on the plot with red dots, and are used to construct the boxplots; blue points represent non-responders and are not included in the boxplots.

For the DNA prime regimens, no statistical comparisons were done for the response magnitude of the ELISA responses to EnvC at 4 weeks after the prime due to the small numbers of responders in the three groups. For rAd5 prime regimens, neither the ID nor SC prime groups met the predetermined criteria of a 5-fold increase above the IM group (**Figure 4A**).

For DNA prime regimens at four weeks post-boost, all participants were considered responders as determined by ELISA responses to EnvC. The median response magnitude was 540 in the ID group, 810 in the IM group, and 2430 in the SC group. Neither the ID group nor the SC group met the criteria of a 5-fold increase above IM titers, and neither the ID group nor the SC group was statistically significantly different from the IM group. For rAd5 prime regimens, there were 7/8 responders in the ID group, 8/8 in the IM group, and 7/7 in the SC group and the median response magnitude was 7290 for both the ID and SC groups and 2430 for the IM group. Neither the ID group nor the SC group met the criteria of a 5-fold increase above IM titers, and neither the ID group nor the SC group was statistically significantly different from the IM (**Figure 4B**). This did not change when adjusting for pre-existing Ad5 or when route was tested across both regimens.

Vaccine-induced seropositivity/reactivity (VISP/R) as measured by commercial diagnostic test kits was also monitored throughout the study (Table 5). Forty-four of the 60 subjects had at least 1 positive HIV EIA recorded during the study and 39/44 were either positive or indeterminate by Western blot testing. Forty-two of 54 subjects who completed the Study Week 42 testing remained EIA positive from vaccine-induced antibody; no subjects were HIV-infected.

**Table 5:** Frequency of Vaccine-Induced Seropositivity/Reactivity Through Study Week 42.

	ELISA Results		*Western Blot results					RNA PCR results	
	Negative (−)	Positive (+)	Negative (−)	Uninter-pretable	Indeter-minate	Positive (+)	N/A	Negative (−)	Positive (+)
**DNA ID prime**	2	8	0	0	6	2	2	10	0
**DNA IM prime**	4	6	1	1	3	1	4	10	0
**DNA SC prime**	6	4	0	0	2	2	6	10	0
**rAd ID prime**	3	7	0	0	3	4	3	10	0
**rAd IM prime**	0	10	1	0	6	3	0	10	0
**rAd SC prime**	1	9	1	1	5	2	1	10	0

Subjects were tested at weeks 12, 30 and 42 regardless of the number of vaccines completed. This shows frequency of a “reactive EIA” using a commercial diagnostic kit (Abbott HIV-1/HIV-2 rDNA) at any time during the study. Western blot was performed only for samples with positive EIA. HIV uninfected status was confirmed by RNA PCR tests, which were consistently negative for all subjects throughout the study.

## Discussion

In this study, all three routes of administration for prime injections were well-tolerated, although the local reactogenicity profile is different in some respects for intradermal (ID) and subcutaneous (SC) routes of administration compared to the intramuscular (IM) route. There were more reports of local pruritus following ID and SC injections than reported following IM injections. The type of superficial skin lesion (small erosion/scab) sometimes associated with the DNA vaccine when administered IM by Biojector [17] were not reported for the DNA vaccine when administered by needle and syringe in this study for any of the routes of administration. However, similar superficial skin lesions were observed following the administration of the rAd5 vaccine by intradermal injection. There were also reports of small nodules at the injection site following SC or ID injections.

The study vaccination schedule completion frequency (72% overall) was affected by the protocol amendment made in response to the outcome of the Step Study with the MRK-Ad5 vaccine in which the hazard ratio of HIV-1 infection between vaccine and placebo recipients was higher in Ad5-seropositive, but not Ad5-seronegative, men [6]. The VRC-rAd5 HIV vaccine is different in several ways structurally from the MRK-Ad5 HIV vaccine, including that the VRC-rAd5 vaccine has a partial E3 and complete E4 deletion in addition to the E1 deletion at the site of the insertion of the gene encoding the vaccine antigen [18]. In addition, the cell line in which the two vaccines were produced differ (293-ORF6 for the VRC-rAd5 [2] and PER.C6 for the MRK-rAd5 [19]), and the encoded HIV antigens in the Merck vaccine were Gag, Pol and Nef, while the VRC-rAd5 vaccine encoded Env from three clades of HIV-1, Gag and Pol [18]. Nonetheless, the safety concerns raised by the Step study had to be taken into account during the conduct of the VRC 011 study and this affected the frequency at which the vaccination schedules were completed for Ad5-seropositive subjects.

There was not sufficient evidence of the prospectively defined immunogenicity endpoints for the SC or ID route of administration to suggest that these routes, rather than IM administration route, offer greater magnitude of immune response. With regard to the DNA vaccine, a prior study had shown an immune response advantage to using the Biojector for administration over a standard needle and syringe injection method [17]. The comparison of the immunogenicity results from the VRC 011 study with other studies of these vaccines is limited by the difference in method of administration. Consistent with our results in the VRC 011 route evaluation, studies evaluating the IM and ID injection route of an avian influenza DNA vaccine showed more local reactogenicity with the ID route and although both routes were immunogenic, there was no suggestion of an immunogenicity or dose-sparing advantage for the ID route [20]. Similarly, evaluation of the rAd5 vaccine by ID, SC and IM routes that was performed by the HIV Vaccine Trials Network in the HVTN 069 study showed more local reactogenicity for the ID and SC routes and no apparent immunogenicity advantage [7].

While we did not identify a delivery route that substantially improved the immunogenicity of the DNA prime-rAd5 boost vaccine regimens, all groups demonstrated boosting of antibody responses to the HIV Env. In the groups receiving two rAd5 injections, the pre-existing Ad5 immunity from the first vaccination did not diminish the ability of the adenoviral vector vaccine to induce antibody. This is consistent with previously reported results of DNA-rAd5 and rAd5-rAd5 regimens evaluated in the HVTN 068 study [21].

More than magnitude of immune response, the character of an immune response may be a key factor for achieving protective efficacy. In the interim since the Step Study ended in 2007, much work ensued to better define potential correlates of protection against HIV infection. The RV144 study conducted in Thailand, with a canarypox-vectored prime (vCP1521) followed by a protein vaccine boost with the gp120 AIDSVAX B/E vaccine, is the only HIV vaccine study thus far to show some protection [vaccine efficacy 31.2% (95% CI, 1.1 to 52.1; P = 0.04)] against HIV infection [22]. The RV144 study results, reported in 2009, were followed by laboratory evaluations to assess for potential immune correlates of protection that suggested V1V2-specific IgG was a correlate of protection in this general population cohort [23]. Further analysis of the specificity and function of antibodies induced in the RV144 regimen compared to antibodies elicited by the VRC regimen is ongoing. A T cell response that controls HIV-1 replication [24] in conjunction with an effective antibody response may be necessary to achieve a greater frequency of protection than was seen with the RV 144 vaccine regimen. A more precise understanding of the immune correlates of protection, antigen design, and vaccine formulation and delivery is needed to achieve the goal of vaccine-induced durable protection against HIV infection.

## Supporting Information

Protocol S1 **Trial Protocol.**
(PDF)

Checklist S1 **CONSORT Checklist.**
(DOC)
