# Supplementary material for: Phase I Randomized Clinical Trial of VRC DNA and rAd5 HIV-1 Vaccine Delivery by Intramuscular (IM), Subcutaneous (SC) and Intradermal (ID) Administration (VRC 011)
Source: PLoS One. 2014 Mar 12;9(3):e91366. doi: 10.1371/journal.pone.0091366 (PMC3951381; doi:10.1371/journal.pone.0091366)
Supplement: Protocol S1 — Trial Protocol. (PDF) [file pone.0091366.s001.pdf]

**Version 2.0**
**March 6, 2007**

# VACCINE RESEARCH CENTER

## Protocol VRC 011
## (NIH 06-I-0149)

## A Phase I Clinical Trial of Intramuscular, Subcutaneous and Intradermal Administration of an HIV-1 Multiclade DNA Vaccine, VRC-HIVDNA016-00-VP, and an HIV-1 Multiclade Adenoviral Vector Vaccine,VRC-HIVADV014-00-VP, in Uninfected Adult Volunteers

Vaccines Provided by
Vaccine Research Center/NIAID/NIH, Bethesda, MD

Clinical Trial Sponsored by:
National Institute of Allergy and Infectious Diseases (NIAID)
Vaccine Research Center (VRC)
Bethesda, Maryland

IND Sponsored by:
National Institute of Allergy and Infectious Diseases
Division of AIDS (DAIDS)
Bethesda, Maryland

*BB-IND* 12326 - *held by DAIDS*

Principal Investigator:

Barney S. Graham, M.D., Ph.D.
Vaccine Research Center, National Institute of Allergy and Infectious Diseases (NIAID)
National Institutes of Health (NIH)
Bethesda, MD 20892

IRB Initial Review Date: March 27, 2006

# TABLE OF CONTENTS

Page
Abbreviations Used in VRC 011 .....5
Table of IND and Protocol Numbers Discussed in VRC 011 .....7
Précis .....8

**1. INTRODUCTION AND RATIONALE ..... 10**

1.1 HIV-1: Etiology, Disease Course, and Epidemiology ..... 10
1.2 Previous Experience with the Study Vaccines ..... 10
1.3 Rationale for Evaluating Different Routes of Vaccine Administration ..... 17
*1.3.1 Rationale for Evaluating IM, SC and ID Administration of the DNA Vaccine ..... 19*
*1.3.2 Rationale for Evaluating rAd5 Prime with rAd5 Boost Regimen that Include IM, SC and ID Routes of Administration ..... 22*
1.4 Measures of Immunogenicity ..... 24

**2. BACKGROUND ON VACCINE ..... 25**

2.1 HIV-1 DNA Vaccine Plasmids in VRC-HIVDNA016-00-VP ..... 25
2.2 Adenoviral Vectors in VRC-HIVADV014-00-VP ..... 26
2.3 Preparation of the Bulk Plasmid and Final Product ..... 27
*2.3.1 VRC-HIVDNA016-00-VP ..... 27*
*2.3.2 VRC-HIVADV014-00-VP ..... 28*

**3. STUDY OBJECTIVES ..... 28**

3.1 Primary Objectives ..... 28
3.2 Secondary Objectives ..... 28
3.3 Exploratory Objectives ..... 29

**4. STUDY DESIGN ..... 29**

4.1 Study Population ..... 31
*4.1.1 Inclusion Criteria ..... 31*
*4.1.2 Exclusion Criteria ..... 32*
4.2 Schedule of Clinical Procedures and Laboratory Assays ..... 34
*4.2.1 Screening ..... 35*
*4.2.2 Day 0 through Week 42 Clinical Follow-Up and Week 94 Follow-up ..... 35*
4.3 Monitoring for HIV Infection ..... 40
4.4 Intercurrent HIV Infection ..... 41
4.5 Concomitant Medications ..... 41
4.6 Criteria for Withdrawal of a Subject from Injection Schedule ..... 41
4.7 Criteria for Stopping Study ..... 42

**5. SAFETY AND ADVERSE EVENT REPORTING ..... 43**

5.1 Adverse Events ..... 43
5.2 Serious Adverse Events (SAE) ..... 43
5.3 Adverse Event Reporting to the IND Sponsor ..... 44
5.4 Adverse Event Reporting to the Institutional Review Board ..... 45
5.5 Serious Adverse Event Reporting to the Institutional Biosafety Committee ..... 45

**6. STATISTICAL CONSIDERATIONS ..... 46**

6.1 Overview ... 46
6.2 Objectives ... 46
6.3 Endpoints ... 46
*6.3.1 Safety* ... *46*
*6.3.2 Immunogenicity* ... *46*
*6.3.3 Social Impacts* ... *46*
6.4 Sample Size and Accrual ... 47
*6.4.1 Randomization of Treatment Assignments* ... *47*
*6.4.2 Power Calculations for Safety* ... *47*
*6.4.3 Sample Size Calculations for Immunogenicity* ... *49*
6.5 Statistical Analysis ... 51
*6.5.1 Analysis Variables* ... *51*
*6.5.2 Baseline Demographics* ... *51*
*6.5.3 Safety Analysis* ... *51*
*6.5.4 Immunogenicity Analysis* ... *52*
*6.5.5 Social Impact Analysis* ... *52*
*6.5.6 Interim Analyses* ... *52*

**7. PHARMACY PROCEDURES ... 53**

7.1 Study Agents for Prime-Boost Regimen ... 53
*7.1.1 DNA 6-Plasmid Vaccine, VRC-HIVDNA016-00-VP* ... *53*
*7.1.2 Adenoviral Vector Vaccine, VRC-HIVADV014-00-VP* ... *53*
7.2 Preparation of Study Agent for Administration ... 54
*7.2.1 Preparation of VRC-HIVDNA016-00-VP for Administration by Needle and Syringe* ... *54*
*7.2.2 Preparation of VRC-HIVADV014-00-VP for Administration by Needle and Syringe* ... *54*
7.3 Study Agent Labeling ... 55
7.4 Procedures to Preserve Blinding ... 55
7.5 Study Agent Accountability ... 55
*7.5.1 Documentation* ... *55*
*7.5.2 Disposition* ... *55*

**8. HUMAN SUBJECT PROTECTIONS AND ETHICAL OBLIGATIONS ... 56**

8.1 Informed Consent ... 56
8.2 Risks and Benefits ... 56
*8.2.1 Risks* ... *56*
*8.2.2 Benefits* ... *58*
8.3 Institutional Review Board ... 58
8.4 Protocol Registration ... 58
8.5 Subject Confidentiality ... 59
8.6 Plan for Use and Storage of Biological Samples ... 59
8.7 Subject Identification and Enrollment of Study Participants ... 60
*8.7.1 Participation of Children* ... *60*
8.8 Compensation ... 60
8.9 Safety Monitoring ... 61

**9. ADMINISTRATIVE AND LEGAL OBLIGATIONS .............................................. 61**
9.1 Protocol Amendments and Study Termination ................................................ 61
9.2 Study Documentation and Storage ................................................ 61
9.3 Study Monitoring, Data Collection and Data Monitoring ................................................ 62
*9.3.1 Study Monitoring ................................................ 62*
*9.3.2 Data Collection ................................................ 62*
9.4 Language ................................................ 62
9.5 Policy Regarding Research-Related Injuries ................................................ 62
**10. REFERENCES ................................................ 63**

## APPENDICES

I Study Informed Consent Form ................................................ 68
II Contact Information ................................................ 85
III Schedule of Evaluations ................................................ 87
IV Table for Grading Severity of Adverse Events ................................................ 92

# Abbreviations Used in VRC 011

| Abbreviation | Term |
|---|---|
| AAV | adeno-associated virus |
| Ab | antibody |
| Ad | adenovirus |
| Ad5 | adenovirus 5 |
| ADL | activities of daily living |
| AE | adverse event |
| AIDS | Acquired Immunodeficiency Syndrome |
| ALT | alanine aminotransferase |
| ANC | absolute neutrophil count |
| AoU | Assessment of Understanding |
| APC | antigen presenting cells |
| AST | aspartate aminotransferase |
| Biojector | Biojector®2000 |
| BMI | body mass index |
| CAB | Community Advisory Board |
| CAVE | Capital Area Vaccine Effort |
| CBC | complete blood count |
| CDC | Centers for Disease Control and Prevention |
| cDNA | complementary deoxyribonucleic acid |
| cGMP | current Good Manufacturing Practices |
| CMV | cytomegalovirus |
| CPK | creatine phosphokinase |
| CsCl | cesium chloride |
| CTL | cytotoxic T lymphocytes |
| DAIDS | Division of AIDS |
| DNA | deoxyribonucleic acid |
| DSMB | Data and Safety Monitoring Board |
| EAE | expedited adverse event |
| ELISA | enzyme-linked immunosorbent assay |
| ELISpot | enzyme-linked immunospot assay |
| Env | envelope |
| FACS | fluorescence-activated cell sorter |
| FDA | Food and Drug Administration |
| FFB | final formulation buffer |
| ffu | fluorescent forming unit |
| F-PERT | fluorescent product enhanced reverse transcriptase |
| GCP | Good Clinical Practices |
| GLP | Good Laboratory Practices |
| GMP | Good Manufacturing Practices |
| GMT | geometric mean titer |
| gp | glycoprotein |
| HAART | highly active antiretroviral therapy |

| | |
|---|---|
| HBV | hepatitis B virus |
| HCV | hepatitis C virus |
| HHV | human herpes virus |
| HIV | human immunodeficiency virus |
| HLA | human leukocyte antigen |
| HTLV | human T cell leukemia virus |
| HVTN | HIV Vaccine Trials Network |
| IBC | Institutional Biosafety Committee |
| ICH | International Conference on Harmonisation |
| ICS | intracellular cytokine staining |
| ID | intradermal |
| IFN-$\gamma$ | interferon gamma |
| IM | intramuscular |
| IND | investigational new drug application |
| IRB | Institutional Review Board |
| ITT | intent-to-treat |
| LDL | low density lipoprotein |
| LIMS | Laboratory Information Management System |
| LTR | long terminal repeat |
| MCB | master cell bank |
| MPW | medical pathology waste |
| MUWRP | Makerere University-Walter Reed Project |
| NIAID | National Institute of Allergy and Infectious Diseases |
| NIH | National Institutes of Health |
| N/S | needle/syringe |
| NSAID | nonsteroidal anti-inflammatory drug |
| NVITAL | NIAID Vaccine Immune T-Cell and Antibody Laboratory |
| OD | optical density |
| OTCD | ornithine transcarbamylase deficiency |
| P*f*CSP | *Plasmodium falciparum* circumsporozoite |
| PPD | purified protein derivative |
| PBMC | peripheral blood mononuclear cells |
| PBS | phosphate buffered saline |
| PCR | polymerase chain reaction |
| pfu | plaque forming unit |
| Pol | polymerase |
| PT | prothrombin time |
| PTT | partial thromboplastin time |
| PU | particle unit |
| rAd5 | recombinant adenoviral vector vaccine |
| RAC | recombinant DNA advisory committee |
| RCA | replication-competent adenovirus |
| RCC | Regulatory Compliance Center |
| RPR | rapid plasma reagin |
| SAE | serious adverse event |

| | |
|---|---|
| SARS | Severe Acute Respiratory Syndrome |
| SC | subcutaneous |
| SD | study day |
| SFU | spot-forming units |
| SHIV | simian/human immunodeficiency virus |
| SIV | simian immunodeficiency virus |
| T1S1 | transcriptionally inert spacer element |
| ULN | upper limit of normal |
| UNAIDS | Joint United Nations Programme on HIV/AIDS |
| VRC | Vaccine Research Center |
| WBC | white blood cell |
| WFI | water for injection |
| WNV | West Nile virus |

## Table of IND and Protocol Numbers Discussed in VRC 011

| IND Number | Vaccines | VRC Protocol Identifier | NIH protocol number |
|---|---|---|---|
| **HIV DNA VACCINES** | | | |
| BB-IND 10681 | VRC-HIVDNA009-00-VP | **VRC 004**<br>HVTN 052<br>RV 156 | 03-I-0022 |
| BB-IND 11661 (preventive) | VRC-HIVADV014-00-VP | **VRC 006**<br>*HVTN 054* | 04-I-0172 |
| BB-IND 11750 | VRC-HIVDNA016-00-VP | **VRC 007** | 04-I-0254 |
| BB-IND 11894 (preventive) | VRC-HIVDNA009-00-VP prime<br>VRC-HIVADV014-00-VP boost | **VRC 009**<br>*HVTN 057*<br>*HVTN 068*<br>*RV 156A* | 05-I-0081 |
| BB-IND12326 | VRC-HIVDNA016-00-VP prime<br>VRC-HIVADV014-00-VP boost | **VRC 008**<br>**VRC 010**<br>*HVTN 204*<br>*IAVI-V001*<br>*RV-172* | 05-I-0148<br>05-I-0140 |
| **WNV DNA VACCINE** | | | |
| BB-IND 12242 | VRC-WNVDNA017-00-VP | **VRC 302** | 05-I-0126 |

# Précis

**Protocol VRC 011:** A Phase I Clinical Trial of Intramuscular, Subcutaneous and Intradermal Administration of an HIV-1 Multiclade DNA Vaccine, VRC-HIVDNA016-00-VP, and an HIV-1 Multiclade Adenoviral Vector Vaccine, VRC-HIVADV014-00-VP, in Uninfected Adult Volunteers

**Study Design:** The VRC DNA vaccine and VRC recombinant adenoviral vector (rAd5) vaccine have been previously shown to elicit immune responses to HIV-1-specific peptides when administered intramuscularly (IM) alone and in prime-boost schedules. This Phase I, randomized, open-label exploratory study will evaluate the safety and tolerability and the immune responses when IM, subcutaneous (SC) or intradermal (ID) routes of administration are used for the priming vaccinations in a prime-boost schedule. The randomization will ensure that subjects with negative and positive screening adenovirus type 5 antibody (Ad5Ab) titers will be equally represented in each prime-boost schedule evaluated in the study. Group 1 subjects will receive three DNA prime vaccinations followed by a rAd5 boost vaccination and Group 2 subjects will receive one rAd5 prime vaccination followed by a rAd5 boost vaccination. It is also of interest to explore whether vaccination by SC or ID route alters the functional qualities of the immune response. About half of the subjects who screen for HIV vaccine studies at the VRC Clinic have negative Ad5Ab titer and half have positive Ad5Ab titers.

The hypotheses are: 1) IM, SC and ID are all safe routes of administration for both the DNA and rAd5 vaccines; 2) all regimens will elicit immune responses to HIV-1-specific peptides; 3) intradermal administration will allow a lower dosage of the DNA vaccine to be used for eliciting an immune response; and 4) rAd5 booster administered after a rAd5 prime will boost the cellular and humoral immune response. The primary objectives relate to evaluation of the safety and tolerability of the DNA and rAd5 vaccines when administered by IM, SC and ID routes. Secondary objectives are related to evaluation of the immunogenicity of the vaccines when administered by SC and ID routes as compared to the IM route and the social impact of participating in an HIV-1 vaccine trial. Exploratory evaluations of the immunogenicity of the vaccination regimens are also planned.

**Product Description:** VRC-HIVDNA016-00-VP (DNA vaccine) is composed of 6 closed, circular DNA plasmids that encode HIV-1 Gag, Pol and Nef (from clade B) and Env glycoprotein from clade A, clade B, and clade C; each plasmid comprises16.67% (by weight) of the vaccine. VRC-HIVADV014-00-VP (rAd5 vaccine) is composed of 4 recombinant non-replicating adenoviral vectors that encode for HIV-1 Gag/Pol polyproteins (from clade B) and Env glycoprotein from clade A, clade B, and clade C, which are combined in a 3:1:1:1 ratio, respectively.

**Subjects:** Sixty healthy adult volunteers, 18 to 50 years old, 30 subjects with negative Ad5Ab titers (<1:12) and 30 subjects with positive Ad5Ab titers (≥1:12).

**Study Plan:** Subjects with negative and positive Ad5Ab titers will be equally randomized to the six prime-boost schedules evaluated in the study as shown in the schema below. All injections will be administered by a needle and syringe device appropriate for the route of administration specified.

| DNA prime with rAd5 boost schedules | Group 1 | pre-entry Ad5Ab Titer | N= | DNA Prime Day 0 | DNA Prime Day 28±7 | DNA Prime Day 56±7 | rAd5 Boost Day 168 (-7, +14 days) |
|---|---|---|---|---|---|---|---|
| | | | | (at least 21 days between DNA vac.) | | | |
| IM prime (N=10) | 1A | <1:12 | 5 | 4 mg IM | 4 mg IM | 4 mg IM | $10^{10}$ PU IM |
| | 1B | ≥1:12 | 5 | 4 mg IM | 4 mg IM | 4 mg IM | $10^{10}$ PU IM |
| SC prime (N=10) | 1C | <1:12 | 5 | 4 mg SC | 4 mg SC | 4mg SC | $10^{10}$ PU IM |
| | 1D | ≥1:12 | 5 | 4 mg SC | 4 mg SC | 4mg SC | $10^{10}$ PU IM |
| ID prime (N=10) | 1E | <1:12 | 5 | 400 μg ID | 400 μg ID | 400 μg ID | $10^{10}$ PU IM |
| | 1F | ≥1:12 | 5 | 400 μg ID | 400 μg ID | 400 μg ID | $10^{10}$ PU IM |
| **rAd5 prime with rAd5 boost schedules** | **Group 2** | **pre-entry Ad5 Ab Titer** | **N=** | **rAd5 Prime Day 0** | **-** | **-** | **rAd5 Boost Day 168 (-7, +14 days)** |
| IM prime (N=10) | 2A | <1:12 | 5 | $10^{10}$ PU IM | - | - | $10^{10}$ PU IM |
| | 2B | ≥1:12 | 5 | $10^{10}$ PU IM | - | - | $10^{10}$ PU IM |
| SC prime (N=10) | 2C | <1:12 | 5 | $10^{10}$ PU SC | - | - | $10^{10}$ PU IM |
| | 2D | ≥1:12 | 5 | $10^{10}$ PU SC | - | - | $10^{10}$ PU IM |
| ID prime (N=10) | 2E | <1:12 | 5 | $10^{10}$ PU ID | - | - | $10^{10}$ PU IM |
| | 2F | ≥1:12 | 5 | $10^{10}$ PU ID | - | - | $10^{10}$ PU IM |
| **TOTAL** | 2 groups<br>6 schedules | N=60<br>[30 Ad5Ab neg.;<br>30 Ad5Ab pos.] | | DNA prime N= 30 (90 DNA vacs.)<br>rAd5 prime N=30 (30 rAd5 prime vacs) | | | 60 rAd5 boosts |

**Study Duration**: Each vaccination regimen and clinical follow-up schedule requires 42 weeks on study. A long-term follow-up clinic visit one year later (week 94) will be encouraged to allow HIV testing, long-term immunology evaluation and interview about significant health changes. However, subject may opt for long-term contact to occur by telephone, e-mail or mail for the interview only. Subjects with vaccine-induced HIV antibody may have annual HIV testing at NIH for five years after the last required clinic visit.

# 1. INTRODUCTION AND RATIONALE

## 1.1 HIV-1: Etiology, Disease Course, and Epidemiology

Worldwide, the rate of new human immunodeficiency virus (HIV) infections continues to increase at an unacceptably high level. Although new Acquired Immunodeficiency Syndrome (AIDS) diagnoses and deaths have fallen significantly in developed countries since the advent of highly active antiretroviral therapy (HAART), in the developing world the HIV/AIDS epidemic continues to accelerate [1]. According to the Joint United Nations Programme on HIV/AIDS (UNAIDS) and the World Health Organization, as of the end of 2004, it is estimated that 35.9-44.3 million people are living with HIV/AIDS, including an estimated 4.3-6.4 million new cases in 2004 [2]. Worldwide there were an estimated 2.8-3.5 million deaths due to HIV/AIDS in 2004 [2] and there have been as many as 30 million deaths as a result of HIV infection since the beginning of the epidemic [1].

Beyond the human tragedy of HIV/AIDS, the costs of the epidemic pose a significant impediment to the economic growth and political stability of many countries. In developing countries and in segments of the U.S. population, anti-HIV therapies are frequently beyond financial reach. Accordingly, effective, low-cost tools for HIV prevention, such as a vaccine, are urgently needed to bring the HIV epidemic under control. For this reason, the Vaccine Research Center (VRC) and Division of AIDS (DAIDS) at the National Institute of Allergy and Infectious Diseases (NIAID) of the National Institutes of Health (NIH) are committed to the development of safe, effective vaccines to prevent HIV infection and AIDS worldwide.

The use of multivalent vaccines, containing a defined mixture of immunogens from a number of prevalent subtypes should be a feasible approach to achieve broadly-protective HIV vaccines. The World Health Organization UNAIDS HIV Vaccine Advisory Committee has recommended that candidate HIV vaccines be designed based upon the strains prevalent in the country in which trials are to be conducted [3]. The Vaccine Research Center, NIAID, NIH and the World Health Organization-Joint United Nations Programme on HIV/AIDS organized a meeting focused on the genetic diversity of HIV and strategies to develop vaccine candidates. A consensus was reached that generation of multiclade candidate vaccines is a high international scientific priority [4]. This approach is the foundation for the multiclade design of the prime-boost vaccination regimen development strategy. The DNA vaccine,VRC-HIVDNA016-00-VP, as well as the adenoviral vector vaccine, VRC-HIVADV014-00-VP encode *gag* and *pol* gene sequences from clade B as well as more diverse *env* genes from clades A, B and C. The DNA vaccine also encodes a clade B *nef* gene sequence. Clades A, B and C together represent the viral subtypes responsible for about 85% of new HIV infections in the world [5].

## 1.2 Previous Experience with the Study Vaccines

**DNA Vaccine Dose and Safety:** The Vaccine Research Center (VRC), National Institute of Allergy and Infectious Diseases (NIAID), National Institutes of Health (NIH), in collaboration with the Division of AIDS (DAIDS), NIAID, NIH have conducted a series of clinical studies with two similar multiclade deoxyribonucleic acid (DNA) vaccines. These are the multiclade 4-plasmid DNA vaccine (VRC-HIVDNA009-00-VP) and the multiclade 6-plasmid DNA vaccine (VRC-HIVDNA016-00-VP) that will be used in this study. Table 1A below shows the status as of December 2005 of both intramural and extramural studies in which these vaccines have been

administered to healthy subjects.

### Table 1A: Experience with VRC Multiclade HIV-1 DNA Vaccines in Uninfected Subjects

| HIV DNA vaccine formulation | Study | Dose (mg) | # active doses (subjects in active arm) | Comment |
|---|---|---|---|---|
| **VRC-HIVDNA009-00-VP**<br>4 plasmids (multiclade):<br>clade B *gag-pol-nef,*<br>clade A *env*,<br>clade B *env*,<br>clade C *env*. | VRC 004<br>(BB-IND 10681) | 2<br>4<br>8 | 15 (5)<br>60 (20)<br>44 (15) | Study unblinded Sept 2004; also included 10 placebo subjects (1 placebo and 1 of 8 mg doses not given). |
| | HVTN 052 (BB-IND 10681) | 4 | ~300 (120) | Completed October 2005. Of 540 blinded doses (300 active and 240 placebo), 8 total were not given (still blinded). |
| | RV156<br>(BB-IND 10681) | 4 | ~45 (15) | Completion expected May 2006. Also includes 15 placebo subjects (45 placebo injections) |
| | HVTN 068<br>(BB-IND 11894) | 4 | enrollment in progress | Planned accrual: 66 subjects (6 get placebo; 30 get 4-plasmid DNAx2 with rAd5 boost and 30 get rAd5 prime with rAd5 boost). Open to accrual February 2006.. |
| **VRC-HIVDNA016-00-VP**<br>6 plasmids (multiclade):<br>clade B *gag,*<br>clade B *pol*,<br>clade B *nef*,<br>clade A *env*,<br>clade B *env*,<br>clade C *env*. | VRC 007<br>(BB-IND 11750) | 4 | 44 (15) | Study completed July 2004. Open label; 1 injection not given. |
| | VRC 008<br>(BB-IND 12326) | 4 | 120 (40) | Ongoing study. Enrolled 40 subjects (May-Sept 2005); no placebos. |
| | HVTN 204<br>(BB-IND 12326) | 4 | enrollment in progress | Planned accrual is 480 subjects (240 will receive placebo). Open to accrual Sept 2005. |
| | IAVI V001,<br>(BB-IND 12326) | 4 | enrollment in progress | Planned accrual is 104 subjects (26 will receive placebo; 24 will receive rAd5 only). Open to accrual November 2005. |
| | RV 172<br>(BB-IND 12326) | 4 | enrollment pending | Planned accrual is 324 subjects (138 will receive placebo; 48 will receive rAd5 only). |

Protocols VRC 004 (03-I-0022) and VRC 007 (04-I-0254) were conducted by the VRC Clinic at the NIH Clinical Center (NIH CC) to evaluate two similar multiclade DNA vaccines.

The VRC 004 study was a Phase I randomized, placebo-controlled, dose-escalation study of the 4-plasmid DNA vaccine, VRC-HIVDNA009-00-VP. Unblinded results indicated that dosages of DNA vaccines up to 8 mg are safe and well-tolerated. The 4 mg dose was chosen for further evaluation as administration in a three vaccination schedule resulted in a promising immune response that is easier and less costly to administer than the 8 mg dose. Adverse events possibly related to vaccination included one case each of urticaria (grade 3), maculopapular rash (grade 2) and transient, asymptomatic neutropenia (grade 3); all resolved without sequelae. The VRC 004 immunogenicity results also suggested that the Gag/Pol/Nef fusion construct was not immunogenic in this context and could be improved.

The VRC 007 study was a small open-label study to evaluate a 4 mg dosage of a modified 6-plasmid DNA vaccine, VRC-HIVDNA016-00-VP. Changes to the construct included use of a

different promoter, separate DNA plasmids for the sequences encoding *gag, pol* and *nef*, and encoding for an additional 68 amino acids in the *gag* plasmid. Study results indicate that the 6-plasmid DNA vaccine is safe and well-tolerated. In the VRC 007 study one case of chronic urticaria (grade3) that was well controlled by self-administered antihistamines appears to have started after the second vaccination but was not diagnosed until the subject was nearing study completion. Because no other etiology for the urticaria could be confirmed, it was assessed as possibly related to study vaccine. An unexpected local injection site reaction observed after the 6-plasmid DNA vaccination was mild cutaneous lesions (0.5-1.0 cm diameter) at the vaccination site that occurred after 4 of 44 (9%) vaccinations [in 3 of 15 (20%) subjects].

The VRC 008 (05-I-0148) study opened in May 2005. As of December 2005 the planned 120 injections of the 6-plasmid DNA vaccine have been administered intramuscularly; half by Biojector® 2000 (hereafter referred to as "Biojector") jet injection device and half by needle and syringe. To date, 17 of 20 (85%) subjects who had one or more DNA vaccinations by Biojector have been found by clinician exam to have a small cutaneous papule or superficial skin lesion (0.1-0.5 cm diameter) at the injection site following one or more injections, however, only 6 of 20 (30%) subjects report awareness of these on diary cards. No papules or skin lesion have been observed by clinicians or reported by any of the 20 subjects who have had one or more DNA vaccinations by needle and syringe injection. Following the DNA vaccinations, local reactogenicity has been reported as mild in 35 of 40 (87.5%) and none in 5 of 40 (12.5%). The worst severity of systemic reactogenicity was reported as none in 19/40 (47.5%), mild in 17/40 (42.5%) and moderate in 4/40 (10%) subjects; the moderate symptoms included malaise and/or headache. Systemic symptoms overall included malaise in 18/40 (45%), myalgia in 9/40 (22%), headache in 14/40 (35%), chills in 2/40 (5%), nausea in 3/40 (7.5%) and fever in 1/40 (2.5%); the latter was a temperature of 37.7º C; defined as mild fever by severity grading criteria.

The reactogenicity of the two DNA vaccines at the 4 mg dosage, as reported in the final VRC 004, VRC 007 and VRC 008 diary cards, is shown in Table 1B. Among the 75 vaccinated subjects in these 3 studies, one reported mild fever following vaccination with either of the DNA vaccines administered. Malaise, myalgia and/or headache were both the most frequently recorded systemic symptoms and those included in the "moderate" severity systemic reactogenicity reported by 20% of subjects. Pain was the only local symptom reported as moderate in severity by some subjects.

## Table 1B: Reactogenicity of HIV-1 Multiclade DNA Vaccines at 4 mg in VRC Studies

| 4 mg DNA vaccine Experience | VRC 004 Biojector N=20 | VRC 007 Biojector N=15 | VRC 008 Biojector N=20 | VRC 008 Needle/Syringe N=20 |
|---|---|---|---|---|
| **Local Symptoms** | | | | |
| None | 1 (5.0%) | 0 | 0 ( 0.0%) | 5 ( 25.0%) |
| Mild | 14 (70.0%) | 13 (86.7%) | 20 (100.0%) | 15 ( 75.0%) |
| Moderate | 5 (25.0%) | 2 (13.3%) | 0 ( 0.0%) | 0 ( 0.0%) |
| Severe | 0 | 0 | 0 ( 0.0%) | 0 ( 0.0%) |
| **Systemic Symptoms** | | | | |
| None | 5 (25.0%) | 7 (46.7%) | 12 ( 60.0%) | 7 ( 35.0%) |
| Mild | 11 (55.0%) | 7 (46.7%) | 8 ( 40.0%) | 9 ( 45.0%) |
| Moderate | 4 (20.0%) | 1 (6.7%) | 0 ( 0.0%) | 4 ( 20.0%) |
| Severe | 0 | 0 | 0 ( 0.0%) | 0 ( 0.0%) |

The extramural studies, HVTN 052 and RV 156 are randomized, placebo-controlled studies that are ongoing. HVTN 052 (N=180; randomized 120 to vaccine and 60 to placebo schedules) is the largest study with the 4-plasmid DNA vaccine, but study results remain blinded. Sixty-one percent of all participants (including placebo recipients) experienced mild or moderate symptoms of systemic reactogenicity (malaise, myalgia, headache, nausea, vomiting, chills or arthralgia), the vast majority of which was mild. Local reactogenicity was reported by 88% of all participants with the worst severity usually mild, less frequently moderate, and in one case reported as severe. The severe injection site pain started 30 minutes after vaccination, was mild by the following day and resolved in 4 days. Other adverse events requiring expedited reporting, but assessed as unlikely to be related to vaccination, have included one case of newly diagnosed diabetes mellitus and one grade 3 asymptomatic thrombocytosis in a subject with an elevated pre-enrollment platelet count. An unblinded review of HVTN 052 safety data by the HVTN Safety Monitoring Board in February 2005 indicated that there were no significant differences in AEs or SAEs across treatment groups.

**<u>rAd5 Vaccine Dose and Safety</u>:** VRC and DAIDS have also collaborated to complete Phase I evaluation of a recombinant adenoviral vector vaccine (rAd5), VRC-HIVADV014-00-VP Table 1C below shows the status as of December 2005 of both intramural and extramural studies of the rAd5 vaccine.

### Table 1C: Experience with the Adenoviral Vector Vaccine in Uninfected Subjects

| rAd5 vaccine formulation | Study | Dose (PU)<br>(all single dose) | # active<br>rAd5 doses | Comment |
|---|---|---|---|---|
| **VRC-HIVADV014-00-VP**<br>4 adenoviral vectors with inserted plasmids (multiclade):<br><br>clade B *gag-pol*,<br>clade A *env*,<br>clade B *env*,<br>clade C *env*. | VRC 006<br>(BB-IND 11661) | $10^9$<br>$10^{10}$<br>$10^{11}$ | 10<br>10<br>10 | rAd5 vaccine alone; study includes 6 placebo subjects. Ad5Ab titer not a factor in eligibility or randomization. |
| | VRC 008<br>(BB-IND 12326) | $10^{10}$<br>$10^{11}$ | ~19<br>~19 | rAd5 boost of 6-plasmid DNA; no placebos. Randomization to enroll equal numbers with low and high Ad5Ab. 2/40 planned boosts not given; dose still blinded. . |
| | VRC 009<br>(BB-IND 11894) | $10^{10}$ | 10 | rAd5 boost of VRC 004 (4-plasmid DNAx3); no placebo subjects. Ad5Ab titer not a factor . |
| | VRC 010<br>(BB-IND 12326) | $10^{10}$ | 4 | rAd5 boost of VRC 007 (6-plasmid DNAx3); No placebo subjects. Ad5Ab not a factor. |
| | HVTN 054<br>(BB-IND 11661) | $10^{10}$<br>$10^{11}$ | 20<br>20 | rAd5 vaccine alone; also includes 8 placebo subjects; all subjects Ad5Ab negative. |
| | HVTN 057<br>(BB-IND 11894) | $10^{10}$ | 60 | rAd5 boost of HVTN 052 (4-plasmid DNA x2 or x3); also includes 10 placebo subjects. Ad5Ab not a factor. |
| | RV 156A<br>(BB-IND 11894) | $10^{10}$ | pending | rAd5 boost of RV 156 (4-plasmid DNAx3) subjects. Ad5Ab not a factor. |

| rAd5 vaccine formulation | Study | Dose (PU) (all single dose) | # active rAd5 doses | Comment |
|---|---|---|---|---|
| | HVTN 068 (BB-IND 11894) | $10^{10}$ | enrollment in progress | Planned accrual: 66 subjects (6 get placebo; 30 get 4-plasmid DNAx2 with rAd5 boost and 30 get rAd5 prime with rAd5 boost). Open to accrual February 2006. |

Protocol VRC 006 (04-I-0172) was conducted by the VRC Clinic and was the first Phase I, randomized, placebo-controlled dose escalation study to evaluate the rAd5 vaccine as a single agent. The unblinded study data indicates that the vaccine is safe for healthy subjects at the three dose levels evaluated. There were no serious adverse events attributed to study vaccine. There were three grade 2 (moderate) adverse events possibly related to vaccination including: 1) asymptomatic neutropenia noted 21 days after study injection in subject with prior history of low neutrophil counts; 2) diarrhea (duration one day) on the third day after study injection and 3) asymptomatic steatohepatitis (fatty liver) which was evaluated by a hepatologist as likely to be a pre-existing condition. When administered as a single agent in VRC 006 the $10^9$ and $10^{10}$ PU (particle unit) doses were associated with less reactogenicity than the $10^{11}$ PU dose. At the $10^{10}$ PU dose none of the subjects had fever and the other reactogenicity was mild or none. At the $10^{11}$ PU dose four subjects had a flu-like set of symptoms that included fever, headache, muscle aches, malaise and/or chills starting within 24 hours after vaccination and lasting several hours. Symptoms were mild to moderate in severity. The $10^{10}$ PU dosage was chosen for the initial evaluation of the rAd5 vaccine as a booster vaccine because it is less reactogenic than the $10^{11}$ PU dose, exhibits significant immunogenicity, and would be less costly to manufacture.

The intramural small Phase I booster studies, VRC 009 (05-I-0081) and VRC 010 (05-I-0140), together provide Phase I safety data for the rAd5 vaccine as a booster for the 4-plasmid and 6-plasmid DNA vaccines, respectively. As shown in Table 1D, the reactogenicity of the rAd5 vaccine at $10^{10}$ PU may be somewhat greater when administered as a booster vaccine.

**Table 1D: Reactogenicity of Multiclade HIV-1 rAd5 Vaccine at $10^{10}$ PU in VRC Studies**

| $10^{10}$ PU rAd5 Experience | VRC 006 (rAd5 alone) N=10 | VRC 009/010 (rAd5 booster) N=14 |
|---|---|---|
| Local Symptoms | | |
| None | 2 (20%) | 0 |
| Mild | 8 (80%) | 13 (93%) |
| Moderate | 0 | 1 (7%) |
| Severe | 0 | 0 |
| Systemic Symptoms | | |
| None | 4 (40%) | 5 (36%) |
| Mild | 6 (60%) | 3 (21%) |
| Moderate | 0 | 6 (43%) |
| Severe | 0 | 0 |

The extramural study, HVTN 057 (N=70; randomized 60 to vaccine and 10 to placebo), is ongoing. This study includes the largest experience with the $10^{10}$ PU dose of the rAd5 vaccine

administered as a booster (to the 4-plasmid DNA vaccine). The still blinded reactogenicity results include local reactogenicity in 79% of participants and systemic reactogenicity in 43% of participants; reactogenicity was generally mild but sometimes moderate in severity. The most commonly reported symptoms were malaise and/or fatigue, myalgia and headache. Six (8.5%) participants reported mild fever.

HVTN 054 is an ongoing Phase I, randomized, placebo-controlled extramural trial to evaluate the rAd5 vaccine as a single agent at both $10^{10}$ PU and $10^{11}$ PU in subjects with no pre-existing adenovirus serotype 5 antibody (Ad5Ab). VRC 008 will also help elucidate whether pre-existing Ad5Ab affects the safety and immunogenicity of the rAd5 vaccine at the$10^{10}$ PU and $10^{11}$ PU doses. Extramural international studies are expected to open in Fall 2006 for further Phase I and II evaluation of the 6-plasmid DNA prime with rAd5 boost in HIV-uninfected subjects.

The rAd5 booster vaccinations in VRC 008 began in November 2005. These include both $10^{10}$ and $10^{11}$ PU vaccinations. Consistent with prior Phase I studies, some of the rAd5 booster vaccinations to date have been followed by the previously noted flu-like symptoms. Vaccinations are ongoing and the dosage administered to each subject remains blinded.

**DNA vaccine and rAd5 vaccine immunogenicity:** The VRC Immunology Core Laboratory has accumulated Phase I data indicating that the study vaccines show enough promising cellular and humoral immunogenicity to warrant further evaluations as preventive HIV vaccines. Although there is a growing body of data on the frequency and magnitude of cellular and humoral immune responses to IM administration, the VRC 011 study will include an IM vaccination cohort to serve as the comparison group for those randomized to SC and ID administration.

Table 1E provides a brief overview of the cellular immune response, as assessed by enzyme-linked immunospot (ELISpot) assay at 4 weeks after three DNA vaccinations (week 12) and 4 weeks after a rAd5 vaccine alone. The greatest frequency and magnitude of response was detected by stimulation with peptide pools representing the EnvA antigen. Preliminary booster data (not shown) in subjects primed with 4- or 6-plasmid DNA and then boosted with the rAd5 vaccine are that the mean ELISpot responses are 3-fold or greater in magnitude compared to those induced by the DNA or rAd5 vaccine alone [6]. For example, EnvA responses as measured by ELISpot are 176, 100 and 112 spot forming units (SFU)/$10^6$ PBMC (arithmetic mean) for the 4-plasmid DNA, 6-plasmid DNA and rAd5 vaccine alone, respectively, while the mean for EnvA in the subjects (n=10) who were boosted in VRC 009 is 1337 SFU/$10^6$ PBMC ($\log_{10}$ GMT = 2.82 [CI 2.41, 3.24]). The confidence intervals for the geometric mean titers (GMT) are shown for VRC 004, VRC 007 and VRC 006 in Table 1E. If the same range of variance around the mean is seen in this study, the group sizes should be adequate to detect a 3-fold difference in EnvA-specific ELISpot responses between any two routes of priming vaccine administration with 80% power. The statistical section of the protocol provides a formal estimate of the power to detect differences in the immune response between groups. Given that the administration of the DNA by the ID route will use a much smaller amount of vaccine, if even 50% of the subjects have a positive response, regardless of the magnitude of the response, the ID route of administration will be considered worth further evaluation in a larger study. Although not detailed in this brief review, T cell responses after DNA prime-rAd5 boost were also evaluated by intracellular cytokine staining (ICS) assays and flow cytometry. These data

are generally consistent with the ELISpot data and have shown that the T cell response to the prime-boost regimen is more polyfunctional than the response to either the DNA vaccine or rAd5 vaccine alone.

**Table 1E**: **Frequency and Magnitude of T cell responses 4 weeks after vaccination as assessed by ELISpot in VRC 004, VRC 007 and VRC 006**

| ELISpot | **VRC 004 (4 mg dose)** | | **VRC 007 (4 mg dose)** | | **VRC 006 ($10^{10}$ PU dose)** | |
|---|---|---|---|---|---|---|
| Peptide Pool | **4-plasmid DNA Week 12 Frequency [Exact 95% CI]** | **Week 12 Mean SFU/$10^6$ PBMC ($log_{10}$ GMT) [95%CI] *n*=19** | **6-plasmid DNA Week 12 Frequency [95% CI]** | **Week 12 Mean SFU/$10^6$ PBMC ($log_{10}$ GMT) [95%CI] *n*=14** | **rAd5 Vaccine Week 4 Frequency [95% CI]** | **Week 4 Mean SFU/$10^6$ PBMC ($log_{10}$ GMT) [95%CI] *n*=10** |
| Env (A) | 14/19 = 74% [49%, 91%] | 175.88 (1.89) [1.54, 2.24] | 9/14 = 64% [35%, 87%] | 99.76 (1.79) [1.49, 2.08] | 6/10 = 60% [26%, 88%] | 112.17 (1.79) [1.45, 2.12] |
| Env (B) | 11/19 = 58% [33%, 88%] | 135.61 (1.77) [1.46, 2.07] | 8/14 = 57% [29%, 82%] | 61.90 (1.65) [1.42, 1.89] | 4/10 = 40% [12%, 74%] | 79.67 (1.75) [1.46, 2.03] |
| Env (C) | 6/19 = 32% [13%, 57%] | 51.93 (1.46) [1.19, 1.72] | 4/14 = 29% [8%, 58%] | 36.07 (1.43) [1.22, 1.65] | 3/10 = 30% [ 7% , 65%] | 57.50 (1.60) [1.32, 1.88] |
| Gag (B) | 1/19 = 5% [ 0%, 26%] | 10.35 (0.88) [0.68, 1.07] | 5/14 = 36% [13%, 65%] | 53.21 (1.54) [1.21, 1.87] | 2/10 = 20% [ 3% , 56%] | 23.33 (1.10) [0.65, 1.55] |
| Nef | 0/19 = 0% [0%, 18%] | 3.33 (0.49) [0.31, 0.67] | 4/14 = 29% [8%, 58%] | 154.76 (1.50) [1.08, 1.91] | N/A | N/A |
| Pol (B)-1 | 0/19 = 0% [0%, 18%] | 5.53 (0.63) [0.41, 0.85] | 0/14 = 0% [0%, 23%] | 7.74 (0.53) [0.19, 0.87] | 2/10 = 20% [ 3% , 56%] | 124.67 (1.13) [0.51, 1.76] |
| Pol (B)-2 | 0/19 = 0% [0%, 18%] | 6.58 (0.69) [0.49, 0.90] | 0/14 = 0% [0%, 23%] | 6.31 (0.66) [0.38, 0.95] | 2/10 = 20% [ 3% , 56%] | 27.50 (1.30) [0.99, 1.60) |
| Any | 14/19 = 74% [49%, 91%] | | 11/14 = 79% [49%, 95%] | | 7/10 = 70% [35%, 93%] | |

In antigen-specific antibody assays, EnvC-specific antigens were associated with the greatest magnitude and frequency of response by research ELISA assay for both the DNA vaccines and the rAd5 vaccine. The geometric mean titers for EnvC antibody responses were in the range of 30-240 for the 4-plasmid DNA and 30-600 for the rAd5 vaccine, with significantly higher responses (e.g. as high as >36,000) after a rAd5 booster vaccination (VRC 009). Detection of a 5-fold difference in EnvC specific response between any two routes of priming vaccine administration should be feasible with the proposed group sizes. As noted above, if 50% of the subjects who receive the DNA vaccine by the ID route have a positive response this will be considered worthy of further evaluation given the much smaller dose of DNA administered ID. Table 1F shows the frequency of positive HIV-1 ELISA responses by commercial diagnostic kits of the vaccines alone and in combination. As with the research ELISA, the magnitude and frequency of the antibody response to the booster administration of rAd5 vaccine is stronger than either the DNA or rAd5 vaccines alone. This is indicated by the higher optical densities of the positive responses (not shown) and the consistently positive response observed after a booster vaccination.

### Table 1F: Frequency of Vaccine-Induced Antibody Response by Commercial Test (maximum at any timepoint)

| Study | HIV-1 RNA PCR | | Commercial ELISA Results | | *Commercial Western Blot Results | | |
|---|---|---|---|---|---|---|---|
| | Neg. | Pos. | Neg. | Pos. | Neg. | Indeter-minate | Pos |
| VRC 004 (4 mg DNA) N=20 | 20 | 0 | 9 (45%) | 11 (55%) | 6 (54.5%) | 5 (45.5%) | 0 |
| VRC 007 (4 mg DNA) N=14 | 14 | 0 | 6 (43%) | 8 (57%) | 1 (12.5%) | 5 (62.5%) | 2 (25%) |
| VRC 006 ($10^{10}$ PU rAd5) N=10 | 10 | 0 | 4 (40%) | 6 (60%) | 2 (33.3%) | 4 (66.7%) | 0 |
| VRC 009/010 ($10^{10}$ PU rAd5) N=14 | 14 | 0 | 0 | 14 (100%) | 0 | 6 (43%) | 8 (57%) |
| *Western Blots are done only for positive ELISA results; greatest response at any timepoint shown. Percentages are percent of positive ELISA. | | | | | | | |

## 1.3 Rationale for Evaluating Different Routes of Vaccine Administration

When licensed vaccines are used, a preferred route of administration is included in the recommendations. Currently licensed vaccines have preferred routes of administration that include IM (e.g., diphtheria, tetanus, pertussis; inactivated influenza, etc), SC (measles, mumps, rubella; varicella, menigococcal polysaccharide, etc), ID (smallpox and BCG) and intranasal (live attenuated influenza). As an investigational vaccine is being evaluated it is important to consider whether the route of administration has an effect on the safety of the vaccine or the quantitative or qualitative aspects of the immune response. Use of a smaller dose by a particular route may affect the cost of developing an effective vaccination strategy. To date, only the IM route of administration has been evaluated for each vaccine. It is known that the jet injection of vaccine by the Biojector device results in a "cone" shaped distribution of injectate. Although the majority of the DNA vaccine administered by Biojector may be deposited in muscle, some portion of the injectate is deposited in skin and another portion is deposited in the subcutis. The observation of mild cutaneous skin papules or lesions associated with the DNA vaccine injection by Biojector, but not by needle and syringe, may be due to the portion that is deposited in the skin or subcutaneous tissue. Vaccine deposited into the skin may elicit more immunogenicity. For example, in VRC 008 among the 38 subjects with Week 12 (4 weeks after 3$^{rd}$ DNA vaccination) results, 0/19 (0%) subjects in the needle and syringe group compared to 6/19 (31.6%) in the Biojector group have a vaccine-induced positive HIV ELISA by commercial diagnostic test.

There are biological reasons why depositing a vaccine in the skin or subcutaneous tissue may induce a different pattern of immune responses than those induced following intramuscular vaccination. The hypothesis underlying intradermal delivery is that antigen presentation by Langerhans cells will be superior to antigen presentation by other dendritic cell (DC) subsets. Langerhans cells are the primary antigen presenting cell (APC) in the skin [7, 8]. Intradermal injections place vaccine in immediate proximity to Langerhans cells and a higher proportion of the DNA plasmids may have the opportunity to directly transduce these APCs. DCs take-up vaccine antigen and with the appropriate activation migrate to regional lymph nodes where they present processed antigens to lymphocytes to initiate the adaptive immune response. It is

possible that targeting Langerhans cells by intradermal injection may require less total DNA to elicit a protective immune response [9, 10]. In order to gain a better understanding of the effect of depositing the injectate in these separate layers of tissue, needle injection will be used in this study to better control where the vaccine is deposited. The theoretical differences in immune response that may occur by targeting different layers of tissue are of interest for both the DNA and the rAd5 vaccine.

Intradermal administration may provide an advantage over other routes of administration if dose-reduction can be achieved while maintaining or improving immunogenicity. This concept has been studied for whole inactivated and subunit vaccines in clinical trials of the licensed trivalent influenza and hepatitis B vaccines.

In an open-label clinical study of trivalent influenza vaccine that included 100 subjects, ages 18-40 years, a reduced-dose ID vaccination elicited similar or superior immunogenicity compared to traditional IM vaccination. Subjects were randomized to be vaccinated with 0.3 µg hemagglutinin per strain in 0.1 mL by intradermal route (1/5 of the standard dose) or with 15 µg hemagglutinin per strain in 0.5 mL IM (standard method). Seroconversion and seroprotection rates were similar in the two groups. The ID vaccinees had more frequent complaints of local reactogenicity, but these were mild and transient and there were no overall differences in systemic symptoms or serious adverse events compared to the intramuscular dose group [11]. In another clinical study of trivalent influenza vaccine, a standard dosage was administered IM to 69 subjects 18-60 years old and 50 subjects ages >60 years old and 40% of the standard dosage was administered ID to 60 subjects 18-60 years old and 58 subject >60 year-old cohort. In the 18-60 year old cohorts a similarly vigorous antibody response was observed for the two methods of administration. The subjects in the >60 year-old cohorts had a vigorous response by ID route but with a trend toward better response by IM [12].

Intradermal administration of hepatitis B vaccine, which is normally administered IM on a three injection schedule, was evaluated in a study that included a group of HIV-infected subjects (N=20) who received 0.1 mL ID of the licensed hepatitis B vaccine (on a 0, 2, 12 month schedule). Induction of antibody concentrations in the protective range was similar to that usually observed with IM administration [13]. A three-injection schedule of hepatitis B vaccine by IM administration of 40 µg (2 mL) was compared to intradermal administration of 20 µg (1 mL total in 2 injections) at 0, 1, and 4 months in chronic hemodialysis patients. The seroconversion rates (HBs-Ab titer >10 IU/L) were 55.6% and 50% in the IM group, and 54.3% and 50% in the ID group, at 1 and 6 months, respectively (p = NS). The ID and IM doses elicited similar seroconversion rates and neither dose route was associated with local or systemic adverse events [10].

Previous preclinical and clinical studies of route of administration of investigational DNA vaccines and investigational rAd5 vaccines have indicated that the IM, SC and ID routes of injection are safe and well-tolerated and that the route of administration may affect the functional properties of the immune response, including the pattern of cytokine production by lymphocytes [14].

### 1.3.1 Rationale for Evaluating IM, SC and ID Administration of the DNA Vaccine

The study plan is to administer the DNA vaccine using standard needle and syringe methods appropriate for the route of administration (IM, SC, ID). The DNA vaccines developed by the VRC have been administered in several different clinical trials intramuscularly (IM) by a needle-free delivery device (Biojector® 2000), manufactured by Bioject Corporation (Portland, OR) from 2001 to the present with more than 300 study subjects total. Beginning in May 2005, DNA vaccinations have been administered IM by standard needle and syringe injection in study VRC 008 (05-I-0148). The IM route has been the only route of administration used to date for VRC DNA vaccines and is the most commonly used route of administration for licensed vaccines.

The wider dispersion pattern of the injectate observed with needle-free injection devices, such as Biojector, and the resultant local inflammatory response may also enhance immunogenicity through increased antigen presentation and recruitment of immune-competent inflammatory cells [15, 16]. For example, it was reported that in rabbits immunized with Biojector, significantly improved antibody response to a *Plasmodium falciparum* circumsporozoite (*Pf*CSP) DNA malaria vaccine was detected as compared to needle and syringe administration [17]. Some human studies have also reported increased antibody response following use of the Biojector device. In a study evaluating trivalent influenza vaccine administered by needle and syringe or by needle-free jet injectors (either VitaJet subcutaneously or Biojector intramuscularly), the Biojector group had higher post-vaccination antibody titers to one of the three influenza antigens [18]. In another study, subjects vaccinated with HAVRIX by Biojector had a significant increase in anti-hepatitis A virus antibody geometric mean titers and seroconversion rates when compared with those vaccinated by needle and syringe [19]. Preliminary data in VRC 008 are that at week 12 (4 weeks after 3rd DNA vaccination) 5/19 (26%) of the Biojector vaccinees had positive vaccine-induced HIV ELISA by commercial assay, whereas 0/19 (0%) of the needle vaccinees had a positive test (one subject in each group have no data for this evaluation). It may be that intentionally injecting more of the vaccine into the subcutis or dermal tissue would increase the magnitude of the immune response.

To date, the SC route of administration has not been the focus of VRC preclinical studies with DNA vaccines, but it is a frequently and safely used route for a variety of injected vaccines and medications. Four VRC investigational DNA vaccines [for HIV, Ebola virus, Severe Acute Respiratory Syndrome (SARS) and West Nile virus (WNV)] have been evaluated under Good Laboratory Practices (GLP) preclinical biodistribution studies, in which the vaccines were administered IM by Biojector to rabbits. A consistent result of the IM administration is that the DNA plasmids were observed to distribute primarily to the subcutis. The following are the briefly stated findings reported in the respective Investigator's Brochures for these DNA vaccines):

<u>HIV DNA vaccine</u>: In a biodistribution study of an HIV DNA vaccine (VRC-HIVDNA006-00-VP), which is similar to the vaccines that were taken into clinical trials, evaluation on days 8, 30, and 60 showed that the highest signals were found in the tissues at or adjacent to the injection site. The magnitude of positive signal produced from day 8 tissues was greatly diminished at the later time points, indicating eventual clearance of the test article. Results from the 8- 30-and 60-

day PCR evaluations showed that the test article was primarily localized to the subcutis at the injection site.

Ebola DNA vaccine: In a biodistribution study of the Ebola DNA vaccine (VRC-EBODNA012-00-VP) in the muscle tissue samples, the frequency of positive tissues and copy number decreased from study day (SD) 8 through SD60. Copy number in the subcutis was greatest at the SD8 necropsy and decreased progressively through the SD30 and 60 necropsies, but the frequency of positive findings did not decrease as dramatically as they did in the muscle samples during the study period. The data suggested that the mechanism of injection by the Biojector may contribute to the low level persistence of the plasmids in the subcutis of the injection site.

SARS DNA vaccine: In a biodistribution study of the SARS DNA vaccine (VRC-SRSDNA015-00-VP) results from the 9- 30- and 61-Day PCR evaluations showed that the test article was primarily localized to the subcutis at the injection site. There was both a higher frequency of positive samples, as well as higher copy count range in subcutis compared to muscle.

WNV DNA vaccine: In the biodistribution study of the WNV vaccine (VRC-WNVDNA017-00-VP), test article was present at the injection site subcutis (9/10 animals on SD9; 1/10 animals on SD31 and 3/10 animals on SD 60) and muscle (7/10 animals on SD 9; 0/10 animals on SD 31; and 0/10 animals on SD 60). The number of copies of the VRC-WNVDNA017-00-VP test article decreased considerably from SD9 to SD60 in all tissues with positive findings and the results were comparable to a plasmid control that was tested concurrently.

Studies of other vaccines have noted an increase in local inflammation in individuals vaccinated via needle-free injection devices [15, 18-21]. The published literature support the idea that the increase in local reactogenicity may reflect the greater distribution of injectate, as well as the minor tissue injury associated with needle-free injection systems [15, 19]. The Department of Safety Assessment, Merck Research Laboratories, published a study that included comparison of needle and syringe to Biojector for intramuscular (IM) administration of DNA plasmid vaccines to guinea pigs. Six weeks after IM injection by either method, the vast majority of the DNA plasmid was in the muscle and skin near the injection site, with skin showing higher copy counts than muscle. Low levels of DNA plasmids were also detectable in draining lymph nodes. In the early timepoints (1-7 days) low systemic exposure could also be detected. Biojector delivery, as compared to needle injection, increased the uptake of DNA plasmids in both muscle and the skin near the injection site, as well being associated with slightly more dispersion to distal sites. Neither method was associated with integration of DNA plasmid into host cellular DNA [22].

Prior to initiating the VRC 008 clinical trial, a preclinical study in non-human primates indicated that the immunogenicity and systemic safety of VRC-HIVDNA016-00-VP are similar when administered IM whether by needle and syringe or by needle-free injection device. Both the published literature and the VRC experience indicate that the Biojector is associated with more injection site reactogenicity than a standard needle and syringe for IM injections. In the VRC's experience with Biojector administration of DNA vaccines to human clinical trials subjects, occasional small (1 mm to 1 cm diameter) cutaneous lacerations have been noted; these have not been seen with the needle and syringe injections of the VRC-HIVDNA016-00-VP vaccine. The mild cutaneous lesion observed with IM Biojector administration, but not IM needle and syringe

administration, may be due to the wider dispersal of the vaccine to subcutaneous and dermal tissue when Biojector is used. Although it is not possible to determine what the biodistribution of the VRC DNA vaccines has been in the human participants in clinical trials, it is presumed on the basis of the VRC rabbit studies, as well as published studies by others, that a significant portion of the vaccine administered IM has distributed to the SC tissue and dermal tissue and remained there for several weeks until fully metabolized and eliminated from the body.

The ID route of administration is less frequently used for vaccinations and medications than either IM or SC routes and has not been evaluated to date for VRC DNA vaccines. However, other investigational DNA vaccines for hepatitis B, influenza, malaria, and diabetes have been evaluated in both animals and humans by the ID route of administration, with comparison to IM route of administration. The published data discussed below support the safety of the ID route for DNA vaccine administration in humans. The ID route may offer the benefit of allowing a good immune response while using a smaller dose of vaccine.

A DNA vaccine against hepatitis B virus (HBV) was evaluated in a human clinical trial for safety and immunogenicity when coated onto gold particles and administered intradermally (ID) using a Powderjet$^{TM}$ system. Vaccination doses were 0.5 μg into two sites (1 μg DNA total); 1 μg DNA into two sites (2 μg DNA total) or 1 μg DNA into four sites (4 μg DNA total) delivered into the upper arm. There were 12 volunteers with 4 in each of the 3 dose groups. Each volunteer was vaccinated at Weeks 0, 8 and16. There were mild and transient local reactions associated with ID injection of particle-mediated DNA vaccinations. A non-hematogenous scab was observed at one site on Day 14. For hepatitis B it is known that a serum anti-HBsAg antibody level of 10 mIU/mL is protective against HBV infection. In the dose range of 1-4 μg ID, the particle-mediated DNA vaccine elicited antibody titers in the protective range for all subjects, but the titers elicited were 4-10-fold lower in peak geometric mean titer than those elicited by plasma-derived or recombinant protein vaccines [14].

In a human clinical trial (N=20) of the *Pf*CSP DNA malaria vaccine four groups of 5 study subjects each were sequentially enrolled to receive three injections intramuscularly by needle and syringe at 4 dose levels that ranged between 20 μg and 2.5 mg per injection. The vaccine was safe and well tolerated when delivered by needle and syringe. A majority of subjects developed antigen-specific CTL responses with more responses in the higher dose groups, but none of the subjects had detectable antibodies to the *Pf*CSP. On the basis of preclinical evaluation of safety and immunogenicity when different injection devices were used for administration, different injection devices were evaluated in a subsequent human clinical trial (N=21). The three injection methods were: needle IM, Biojector IM, and Biojector with 70% of the dose administered IM and 30% administered intradermally (ID). Each of the three groups included 5 vaccinees (2.5 mg dose) and 2 control subjects. The schedule included injections of 1 mL total volume at weeks 0, 4 and 8. The IM/ID administration plan required 4 injections at each time point. Adverse events were generally mild and limited to the injection site with Biojector injections associated with about twice as many adverse events per injection. However, study subjects preferred the Biojector injections [15]. Regardless of the method of administration, anti-*Pf*CSP-specific antibodies could not be detected in the human clinical trial samples, although antigen-specific immune responses as assessed by IFN-γ enzyme-linked immunospot (ELISpot) were detected in all vaccinees [15, 23].

An interim report on a human trial of an HIV-1 DNA (gp120 and Gag) prime with a protein (gp120) boost regimen was presented at the AIDS Vaccine 2005 Conference in Montreal, Canada. In this study DNA vaccinations were administered either IM or ID (1.2 mg total) at weeks 0, 4 and 12 and protein boosts were administered IM with an adjuvant (QS-21) at weeks 20 and 28. HIV-1 specific T cell responses were observed in 3/5 receiving DNA by IM administration and 4/4 receiving DNA by ID administration. Mild skin reactions were the most common adverse event with a more severe skin reaction observed after the protein boost [24, 25].

In a SHIV primate model in which a regimen of DNA (expressing SIV proteins Gag, Pol, Vif, Vpx and Vpr and HIV-1 proteins Env, Tat, Rev) followed by rMVA booster (expressing SIV proteins Gag and Pol and HIV-1 proteins Env) ID and IM routes of administration were compared. The DNA injections were administered as either by ID or IM at doses of 0.25 mg or 2.5 mg with a Bioject (Portland, Oregon). The study included 4 vaccinated groups of 6 rhesus macaques each and 6 unvaccinated animals; all were challenged with the SHIV-89.6P virus. All vaccinated animals became infected, but within the follow-up period 22 of the 24 vaccinated animals controlled the viremia until time of euthanasia at 200 weeks postchallenge at which time these animals had low to undetectable viral loads and normal CD4 count. In the same study 5 of the 6 control animals failed to control viremia and died within a year, while 1 control animal survived with slow recovery of CD4 count. Animals in both the IM and ID vaccination groups had T cell responses that were stable over time and high titers of binding and neutralizing antibodies that persisted [26].

### 1.3.2 Rationale for Evaluating rAd5 Prime with rAd5 Boost Regimen that Include IM, SC and ID Routes of Administration

Clinical studies have evaluated many different routes of administration for investigational adenoviral vector products [27-37]. The side effects were minor, local or absent in most cases where the agents were administered intradermally or intramuscularly, with no significant vector-induced toxicities. A summary of ten clinical trials for the safety parameters [38] and risk factors [39] of low ($<10^9$ PU) and intermediate ($10^9$-$10^{10}$ PU) – dose adenoviral vectors, delivered by various routes (nasal, bronchial, percutaneous to solid tumor, intradermal, epicardial injection of myocardium and skeletal muscle) to 90 individuals and 12 controls for treatment of a variety of conditions (cystic fibrosis, colon cancer metastases, severe coronary artery disease, and peripheral vascular disease) notes that local administration at these doses of adenoviral vector was well tolerated. The major adverse events appeared to be primarily associated with the study population (age, co-morbid conditions) and/or trial procedures (surgery) rather than dose, route of administration, transgene or number of administrations of adenoviral vectors. Although adenoviral vector products have been in clinical trials for several years and are generally safe, a noteworthy exception is that a large dose ($3.8 \times 10^{13}$ PU) of E1/E4-deleted serotype 5 adenoviral vector, which was intended to be a therapeutic product, was administered directly into the liver and caused the death of an 18 year-old patient with the rare liver disorder ornithine transcarbamylase deficiency (OTCD). An NIH report summarizing a review of clinical data from the case concluded that the participant’s death was most likely due to a systemic adenoviral vector-induced shock syndrome, caused by a cytokine cascade that led to disseminated intravascular coagulation, acute respiratory distress and multiorgan failure. Post-mortem bone marrow biopsy revealed red cell aplasia. These data suggested that the high dose of adenoviral

vector delivered directly to the liver quickly saturated available receptors for the vector in that organ and then spilled systemically leading to the fatal response [40]. The particle units of that adenoviral vector product delivered directly into the liver of this patient was 1000-fold more than that planned for the IM, SC and ID vaccinations in this study and is not representative of the experience with adenoviral vector vaccines.

Human experience with the recombinant adenoviral vector vaccine (encoding for Gag, Pol, Nef) developed by Merck was recently presented at the AIDS Vaccine 2005 conference (Montreal, Canada) [41], as well as the plan for evaluating it in a Phase II proof of concept study, which includes a three-dose strategy for administration of the vaccine. Merck reports that a three-dose schedule of their recombinant adenoviral vector vaccine alone to be associated with promising enough immunogenicity to warrant Phase II evaluation. MRKAd5 vaccine experience was also reviewed at the AIDS Vaccine 2004 Conference (Lausanne, Switzerland) [42]. It was noted in 2004 that more than 400 subjects have received one of the investigational MRKAd5 HIV vaccines; the total includes many hundreds more since then. Fever was more common in subjects with low (≤1:200) Ad5 Ab titer and was more common at the 1 x $10^{11}$ viral particle (VP) dose, but the Ad5-based vaccines were generally well tolerated. Preexisting anti-Ad5 neutralizing antibody was also noted to "dampen" the immunogenicity of the Ad5 vector vaccine but this could be overcome at higher dose levels. Therefore, in order to evaluate safety and immunogenicity of rAd5 vector vaccines it is helpful to consider the pre-existing Ad5Ab titers in the randomization schema to either achieve a balance or evaluate either negative or positive subjects only. To date about half the potential study subjects who have screened for enrollment in VRC HIV vaccine studies are Ad5Ab negative (<1:12) and half are Ad5Ab positive (≥1:12). In this study, there will be an equal number of positive and negative subjects in each schedule evaluated in order to gain preliminary information in healthy adults regarding the safety and immunogenicity response to the VRC adenoviral vector vaccine by different routes of administration without biasing the results from each group by having different proportions of negative and positive subjects in one schedule compared to another.

The MRKAd5 HIV vaccine and the VRC rAd5 HIV vaccine have been safe and well-tolerated in healthy volunteers by IM injection at dosages up to $10^{11}$ PU, although more reactogenicity at higher dosages is expected. There is ongoing experience with administering the MRKAd5 vaccine constructs in repeated dosing regimens.

The VRC rAd5 vaccine has not previously been administered in repeated dose regimens in human clinical trials. Preclinical safety studies of the VRC rAd5 vaccine did include a repeat dose toxicology study in New Zealand white rabbits. This study is summarized in the VRC-HIVADV014-00-VP Investigator Brochure and supports the plan for taking a repeat dose regimen into clinical study. HVTN 068 is a recently developed multicenter protocol (submitted to BB-IND 11894) that includes randomization of 30 subjects to repeat dosing schedule of the rAd5 vaccine. In the planned regimen all vaccinations are $10^{10}$ PU by IM administration. VRC 011 as a single site intramural study will include a small cohort of 10 subjects who will receive the same regimen, as well as including two cohorts of 10 each who will receive the first rAd5 vaccinaton by either SC or ID administration. All cohorts will receive the rAd5 booster vaccination by IM administration. The Phase I investigation of the route objective will provide the first opportunity to consider if there are potential advantages to alternate routes and will be complementary to the safety and immunogenicity data provided by HVTN 068.

Published studies of other rAd5 products have also provided data on the administration of recombinant adenoviral vectors to healthy volunteers by the ID route. Adenovirus mediated gene delivery by intradermal (RAC # 9701-171) [30, 39, 43] administration has been tested in healthy volunteers in an effort to define the normal host responses and persistence of the vector. Six healthy volunteers received a single 8 x $10^7$ or 8 x $10^8$ PU ID injection of an $E1^-$, $E3^-$ Ad5-based vector carrying the *E. coli* cytosine deaminase gene $Ad_{GV}$CD.10 [30, 43]. A total of nine healthy volunteers were treated, with one additional subject receiving three administrations of vector and two others receiving a single administration with concomitant oral prednisone. No adverse effects attributed to the vector were observed in any of the nine participants [39]. Detailed cellular reactogenicity was described for a subset of the participants [30]. Skin induration was observed at the injection site in six participants, peaking at day 3 and gradually declining by day 14. Mild or moderate local cellular responses were observed in skin biopsies as measured by cellular infiltration in specimens at all doses studied and adenoviral DNA could be detected in tissue collected 18 days after injection. Modest systemic anti-Ad cellular immune responses were found by quantification of lymphocyte proliferation to adenovirus serotype 5 (Ad5) antigens or $Ad_{GV}$CD.10 vectors in three patients studied. The investigators observed higher peak serum Ad5 neutralizing antibody titers in individuals with evidence of pre-existing antibody to wild-type subgroup C adenovirus prior to treatment, but there was no correlation with the dose of the adenoviral vector. Although these early results suggest that pre-existing immunity to adenovirus may limit the utility of vaccine strategies using repeated dosing of adenoviral vectors [43], as noted above Merck investigators have reported that repeat dosing is a promising strategy.

As with the DNA vaccine (delivered IM by Biojector), the VRC preclinical biodistribution study of the rAd5 vaccine (delivered IM by needle and syringe) indicates that a significant portion of the vaccine distributes to the subcutis. Gene Logic Inc. conducted a single-dose biodistribution study of the rAd5 vaccine, VRC-HIVADV014-00-VP, in New Zealand White rabbits under Good Laboratory Practices (GLP) using intramuscular injections delivered by a needle and syringe. The vaccinated animals received 0.95 x $10^{11}$ PU of VRC-HIVADV014-00-VP in 0.5 mL on SD1. The lower limit of detection for the PCR assay was 10 copies of the target/µg of DNA, the lower limit of quantification for the assay is 50 copies of the target/µg of DNA. The test article was present at the injection site subcutis (5/10 animals on SD 9; 2/10 animals on SD 61) and muscle (4/10 animals on SD 9), with some systemic distribution. The average number of copies was higher in the subcutis (8088 copies target/µg DNA on SD9) than in the muscle (2751 copies target/µg DNA on SD 9) and decreased considerably by SD 61 in all tissues with positive findings. On the basis of this biodistribution study in rabbits, it is presumed that the rAd5 administered IM in human clinical trials is also distributing to SC tissue.

### 1.4 MEASURES OF IMMUNOGENICITY

This Phase I study will provide a preliminary assessment of the immunogenicity of VRC-HIVDNA016-00-VP and VRC-HIVADV014-00-VP in six prime-boost regimens involving IM, SC and ID routes of administration by employing ELISpot and intracellular cytokine staining (ICS) assays that evaluate CTL responses, as well as assays that evaluate HIV-specific antibody responses. Clade-specific peptides will be used to detect T-cell responses by an ELISpot assay modified from a previously published method [44]. The ICS assay is based upon previously published methods [45] and quantitates the frequency of $CD4^+$ and $CD8^+$ cells that produce interleukin-2 or interferon-gamma and other functional parameters of T cell function, in response

to pools of overlapping peptides representing HIV antigens (Gag, Pol, Nef or Env) from specific HIV clades. The ICS will be valuable in observing whether there is evidence of functional differences in the T cell responses elicited by different routes of administration.

The frequency and magnitude of HIV-specific antibodies will be evaluated using an enzyme-linked immunosorbent assay (ELISA) [46].

The ability of the vaccine to elicit neutralizing antibody against HIV-1 strains from clades A, B, and C will be evaluated by a single round of replication Env-pseudovirus assay with a luciferase read-out [47, 48]. The pre-existing and post-vaccination presence of adenovirus serotype 5 neutralizing antibody in study volunteers will be evaluated using a previously published luciferase transgene detection method [49]. Other assays may also be completed from stored samples at a later date if further elucidation of immunogenicity is of interest.

# 2. BACKGROUND ON VACCINE

## 2.1 HIV-1 DNA VACCINE PLASMIDS IN VRC-HIVDNA016-00-VP

VRC-HIVDNA016-00-VP, a six-component multiclade plasmid DNA vaccine, expressing Gag, Pol and Nef proteins from clade B HIV-1 and Env glycoproteins from clades A, B and C, is intended for use as a preventive HIV-1 vaccine. The vaccine has been designed to elicit immune responses against several proteins from a variety of HIV-1 strains. The manufacturer of each plasmid DNA drug substance for preclinical safety studies and clinical trial material, from the establishment of the master cell bank (MCB) through final product, is Vical Incorporated (San Diego, CA). Non-GMP material for preclinical immunological studies was manufactured by Althea Technologies, Inc. (San Diego, CA) by a similar process.

The drug substances for VRC-HIVDNA016-00-VP are comprised of six closed circular plasmid DNA macromolecules, VRC-4401, VRC-4409, VRC-4404, VRC-5736, VRC-5737 and VRC-5738 combined in equal concentrations (mg/mL). VRC 4401 encodes for the clade B HIV-1 Gag structural core protein that encapsidates the viral RNA and exhibits highly conserved domains. VRC-4409 encodes for clade B polymerase (Pol), which is also highly conserved, and VRC-4404 encodes for clade B Nef, an accessory protein against which a vigorous T-cell response is mounted in natural infection. The DNA plasmid expressing HIV-1 Pol has been modified to reduce potential toxicity through the incorporation of changes in the regions affecting the protease, reverse transcriptase, and integrase activities. Two amino acids in the myristoylation site in the HIV-1 *nef* gene were deleted to abrogate MHC class I and CD4+ down-regulation by the Nef protein [50, 51]. No modifications were made to the amino acid sequence of Gag. The other three plasmids express synthetic versions of modified, truncated envelope glycoproteins (gp145) from three strains of HIV-1: VRC-5736 (clade A), VRC-5737 (clade B) and VRC-5738 (clade C). The sequences used to create the DNA plasmids encoding Env are derived from three HIV-1 CCR5-tropic strains of virus. These genes have been modified to improve immunogenicity, which has been demonstrated in mice [52] and monkeys [53]. The vaccine will potentially elicit immune responses to a broad range of HIV-1 strains.

Plasmids containing Gag, Pol, Nef and Env complementary DNA (cDNA) were used to subclone the relevant inserts into plasmid DNA expression vectors that use the CMV/R promoter and the bovine growth hormone polyadenylation sequence. All the plasmids expressing the HIV-1 genes

were made synthetically with sequences designed to disrupt viral RNA structures that limit protein expression by using codons typically found in humans, thereby increasing gene expression. The translational enhancer region of the CMV immediate early region 1 enhancer was substituted with the 5′-untranslated HTLV-1 R-U5 region of the human T-cell leukemia virus type 1 (HTLV-1) long terminal repeat (LTR) to optimize gene expression further. The DNA expression vectors are similar to those used for other candidate vaccines currently undergoing evaluation in clinical studies by the VRC and DAIDS/NIAID/NIH.

The DNA plasmids have been produced in bacterial cell cultures containing a kanamycin selection medium. In all cases, bacterial cell growth is dependent upon the cellular expression of the kanamycin resistance protein encoded by a portion of the plasmid DNA. Following growth of bacterial cells harboring the plasmid, the plasmid DNA is purified from cellular components. The Gag plasmid (VRC-4401) is 5886 nucleotide pairs in length and has an approximate molecular weight of 3.9 MDa; the Pol plasmid (VRC-4409) is 7344 nucleotide pairs in length and has an approximate molecular weight of 4.8 MDa; the Nef plasmid (VRC-4404) is 5039 nucleotide pairs in length and has an approximate molecular weight of 3.3 MDa; the clades A, B, and C Env plasmids (VRC-5736, VRC-5737, and VRC-5738) are 6305, 6338 and 6298 nucleotides in length, respectively, and have an approximate molecular weight of 4.2 MDa.

The plasmid and host *E. coli* strain used in the production of the vaccine are characterized in accordance with the relevant sections of the “Points to Consider in the Production and Testing of New Drugs and Biologicals Produced by Recombinant DNA Technology” (1985), the “Supplement: Nucleic Acid Characterization and Genetic Stability” (1992), “Points to Consider in Human Somatic Cell Therapy and Gene Therapy” (1991, 1998), and “Points to Consider on Plasmid DNA Vaccines for Preventive Infectious Disease Indications” (1996).

## 2.2 ADENOVIRAL VECTORS IN VRC-HIVADV014-00-VP

VRC-HIVADV014-00-VP is a replication-deficient, combination vaccine containing four recombinant adenoviral vectors. These vectors contain gene sequences that code for clade B HIV-1 Gag and Pol as well as clade A, clade B, and clade C Env proteins. *In vitro* expression by these vectors produces immunogens that induce an immune response against HIV. The envelope genes were chosen as representative primary isolates from each of the three clades.

The process for constructing the four VRC-HIVADV014-00-VP recombinant adenoviral vectors is based upon a rapid vector construction system (AdFAST™, GenVec, Inc.) used to generate adenoviral vectors that express the four HIV antigens gp140(A), gp140(B)dv12, gp140(C) and GagPol(B) driven by the cytomegalovirus (CMV) immediate-early promoter. Manufacturing is based upon production in a proprietary cell line (293-ORF6), yielding adenoviral vectors that are replication deficient. The vectors are purified using CsCl centrifugation. The product is formulated as a sterile liquid injectable dosage form for intramuscular injection.

The GV11 adenoviral backbone was chosen to reduce the risk of replication-competent adenovirus (RCA) generation during clinical production. The GV11 backbone contains deletions of two essential regions, E1 and E4, as well as a partial E3 deletion that render the vaccine product replication-deficient. The generation of RCA would require two independent recombination events in a single adenovirus genome, predicted to be an extremely rare event [54].

The $Ad_{GV}$ (HIV).11D vectors contain HIV-1 antigen open reading frame (ORF) expression cassettes inserted to replace the deleted adenovirus E1 gene region. Other deleted adenovirus regions include a partial E3 and all of E4, which has been replaced with a transcriptionally inert spacer element (T1S1) that enhances production of the adenoviral vectors [55].

The 293-ORF6 cell line used to propagate these E1, E4 and partial E3 deleted vectors was developed at GenVec, Inc. These cells were constructed by stably transforming 293 cells (which are of human embryonic kidney origin) with an inducible E4-ORF6 expression cassette. This enables the cells to efficiently complement the E1-, E4-, and partial E3-deleted adenoviral vectors, provide increased transgene capacity and greatly reduce the potential to generate replication-competent adenovirus. The particular clone that has given rise to the cell line is the A232 clone. All references to the 293-ORF6 cell line refer to cells derived from the original A232 clone. This replication-deficient adenoviral vector system has been used to produce TNFerade, a TNF-alpha gene-based product [56]. An assay for replication-competent adenovirus is performed in the final release testing for all vectors; RCA has not been observed in this packaging system during the manufacture of multiple gene-based products.

The four vaccine adenoviral vectors are generated by introducing a DNA plasmid consisting of the adenoviral genome into the 293-ORF6 cells. The adenoviral vector in the lysate from the transfected cells is serially passaged to expand the titer of adenoviral vector. The identity and integrity of the passages is verified by polymerase chain reaction (PCR) and expression of the HIV-1 gene is confirmed by Western Blot analysis. Purified adenoviral vector is produced by infecting the 293-ORF6 cells with the adenoviral vector in the lysate; after the infection of the cells is complete, the material is collected and the vector is purified from the cells. The four vaccine adenoviral vectors are purified using a cesium chloride (CsCl) gradient centrifugation process. CsCl is removed by dialyzing the virus preparation against the final formulation buffer (VRC-DILUENT013-DIL-VP). Purified adenoviral vector serves as a vector bank for subsequent production of the four vaccine adenoviral vectors. This vector bank is tested for sterility, mycoplasma and other adventitious agents prior to its being used for manufacturing of clinical supplies.

## 2.3 PREPARATION OF THE BULK PLASMID AND FINAL PRODUCT

### 2.3.1 VRC-HIVDNA016-00-VP

Bulk plasmid DNA is manufactured at Vical Incorporated (San Diego, CA). One source of each plasmid (supplied by VRC) is used to prepare a Master Cell Bank (MCB) for each plasmid. Vical Incorporated formulates the bulk DNA for each of the components at 4 mg/mL in phosphate buffered saline (PBS). The six plasmids are mixed to form the final bulk vaccine product. Sterile filtration and fill operations are conducted under aseptic conditions in a Class 100 environment. Vical Incorporated fills and performs release testing for the VRC-HIVDNA016-00-VP with the exception of expression, which is tested by the VRC or a subcontractor.

Clinical trial material is tested as bulk plasmid DNA and final product. VRC, or a subcontractor, conducts the gene expression testing on the filled VRC-HIVDNA016-00-VP product and bulk VRC-4401, VRC-4409, VRC-4404, VRC-5736, VRC-5737 and VRC-5738. Upon confirming gene expression, VRC releases the product. Final product meeting all test specifications is released for use in the proposed clinical study.

#### 2.3.2 VRC-HIVADV014-00-VP

The investigational vaccine, VRC-HIVADV014-00-VP, is manufactured by GenVec, Inc. (Gaithersburg, MD) at a contract manufacturer, Molecular Medicine (San Diego, CA). DNA plasmids produced by the Vaccine Research Center, NIAID, NIH (Bethesda, MD) are used to construct the adenoviral vector clinical seed stock. The Phase I clinical production for each adenoviral vector is performed by Molecular Medicine from clinical seed stock produced by Bioreliance (Rockville, MD).

The multiclade adenoviral vector vaccine product, VRC-HIVADV014-00-VP, is a 3:1:1:1 ratio of the adenoviral vectors that encode for HIV-1 Gag/Pol polyprotein from clade B and HIV-1 Env glycoproteins from clades A, B, and C, respectively. Final product meeting all test specifications will be released for use in the proposed clinical study. Vials are filled to 1.2 mL volume with 1 x$10^{10}$ PU/mL or 1 x $10^{11}$ PU/mL.

The final formulation buffer (FFB) is custom manufactured by Cambrex (Walkerville, MD). The FFB is composed of sodium chloride, Tris buffer, trehalose•$2H_2O$ (low endotoxin), magnesium chloride•$6H_2O$, monooleate (Tween 80) and water for injection (WFI).

## 3. STUDY OBJECTIVES

### 3.1 PRIMARY OBJECTIVES

- To evaluate the safety and tolerability of the IM, SC and ID routes of administration for the VRC-HIVDNA016-00-VP vaccine priming injections when dosages of 4 mg, 4 mg and 400 µg, respectively, are administered.
- To evaluate the safety and tolerability of the IM, SC and ID routes of administration for the VRC-HIVADV014-00-VP vaccine priming injections when $10^{10}$ PU dosage is administered.
- To evaluate the safety and tolerability of a $10^{10}$ PU IM booster dose of VRC-HIVADV014-00-VP vaccine when administered after 6 different priming vaccination schedules.

### 3.2 SECONDARY OBJECTIVES

- To evaluate whether VRC-HIVDNA016-00-VP at a dose of 4 mg SC results in at least a 3-fold increase compared to a dose of 4 mg IM in the magnitude of antigen-specific T cells responses to EnvA by ELISpot or 5-fold increase in antigen-specific ELISA (antibody) responses to EnvC at 4 weeks after completing the priming vaccinations (*i.e.*, at Study Week 12).
- To evaluate whether VRC-HIVDNA016-00-VP at a dose of 400 µg ID results in a positive antigen-specific T cells responses to EnvA by ELISpot or antigen-specific ELISA(antibody) responses to EnvC at 4 weeks after completing the priming vaccinations (*i.e.*, at Study Week 12) in 5 of 10 (50%) of subjects.
- To evaluate whether VRC-HIVADV014-00-VP at a dose of $10^{10}$ PU SC or $10^{10}$ PU ID results in at least a 3-fold increase compared to $10^{10}$ PU IM in the magnitude of antigen-specific T cells responses to EnvA by ELISpot or 5-fold increase in antigen-specific

ELISA(antibody) responses to EnvC at 4 weeks after completing the priming vaccination (*i.e.*, at Study Week 4).

- To evaluate adenovirus serotype 5 neutralizing antibody titers at 4 weeks after the first VRC-HIVADV014-00-VP injection administered; this will be a priming vaccination in the Group 2 (rAd5 prime-rAd5 boost) schedules and a booster vaccination in the Group 1 (DNA prime-rAd5 boost) schedules.
- To monitor the social impact of participating in an HIV-1 vaccine clinical trial.

### 3.3 Exploratory Objectives

- To evaluate whether vaccine delivery route influences the functional or phenotypic properties of the HIV-specific T cell responses as measured by multi-parameter flow cytometry.
- To evaluate whether for each of the six priming vaccination schedules, the VRC-HIVADV014-00-VP booster of $10^{10}$ PU IM results in similar frequency and magnitude of T cell responses (intracellular cytokine staining and ELISpot) and antibody response (vaccine antigen-specific ELISA) at 4 weeks after completing the booster vaccination.
- To evaluate the frequency and magnitude of immune responses to the six prime-boost regimens as indicated by intracellular cytokine staining, ELISpot, vaccine antigen-specific ELISA, neutralization assays and other immunological assays at intervals throughout the study.
- To evaluate the long-term immunogenicity of the prime-boost regimen in subjects who agree to have blood drawn at about Week 94.

## 4. STUDY DESIGN

This Phase I, randomized, open-label study will evaluate the safety and tolerability of and the immune responses to two different prime-boost regimens when IM, subcutaneous (SC) or intradermal (ID) routes of administration are used for the priming vaccinations. Sixty HIV-uninfected subjects (18-50 years old), 30 with negative (<1:12) screening adenovirus type 5 antibody (Ad5Ab) titers and 30 with positive (≥1:12) Ad5Ab screening titers, will be equally randomized to six vaccination schedules. Group 1 includes three schedules of DNA prime with rAd5 boost in which equal numbers of subjects (N=10) will have the DNA prime vaccinations administered by IM, SC and ID routes, respectively. Group 2 includes three schedules of rAd5 prime with rAd5 boost, in which equal numbers of subjects (N=10) will have the rAd5 prime vaccination administered by the IM, SC or ID routes, respectively. In both groups for all schedules the rAd5 boost injection will be administered IM. The study will allow exploratory observation of the immune response when different routes of administration are used. By ensuring a balance of pre-entry Ad5Ab titers in each schedule any effect of the pre-entry Ad5Ab titer will not disproportionately affect one schedule more than another. The schema is shown in the table that follows:

| **DNA prime with rAd5 boost schedules** | **Group 1** | **pre-entry Ad5Ab Titer** | **N=** | **DNA Prime Day 0** | **DNA Prime Day 28±7** | **DNA Prime Day 56±7** | **rAd5 Boost Day 168 (-7, +14 days)** |
|---|---|---|---|---|---|---|---|
| | | | | (at least 21 days between DNA vac.) | | | |
| IM prime (N=10) | 1A | <1:12 | 5 | 4 mg IM | 4 mg IM | 4 mg IM | $10^{10}$ PU IM |
| | 1B | ≥1:12 | 5 | 4 mg IM | 4 mg IM | 4 mg IM | $10^{10}$ PU IM |
| SC prime (N=10) | 1C | <1:12 | 5 | 4 mg SC | 4 mg SC | 4mg SC | $10^{10}$ PU IM |
| | 1D | ≥1:12 | 5 | 4 mg SC | 4 mg SC | 4mg SC | $10^{10}$ PU IM |
| ID prime (N=10) | 1E | <1:12 | 5 | 400 μg ID | 400 μg ID | 400 μg ID | $10^{10}$ PU IM |
| | 1F | ≥1:12 | 5 | 400 μg ID | 400 μg ID | 400 μg ID | $10^{10}$ PU IM |
| **rAd5 prime with rAd5 boost schedules** | **Group 2** | **pre-entry Ad5 Ab Titer** | **N=** | **rAd5 Prime Day 0** | **-** | **-** | **rAd5 Boost Day 168 (-7, +14 days)** |
| IM prime (N=10) | 2A | <1:12 | 5 | $10^{10}$ PU IM | - | - | $10^{10}$ PU IM |
| | 2B | ≥1:12 | 5 | $10^{10}$ PU IM | - | - | $10^{10}$ PU IM |
| SC prime (N=10) | 2C | <1:12 | 5 | $10^{10}$ PU SC | - | - | $10^{10}$ PU IM |
| | 2D | ≥1:12 | 5 | $10^{10}$ PU SC | - | - | $10^{10}$ PU IM |
| ID prime (N=10) | 2E | <1:12 | 5 | $10^{10}$ PU ID | - | - | $10^{10}$ PU IM |
| | 2F | ≥1:12 | 5 | $10^{10}$ PU ID | - | - | $10^{10}$ PU IM |
| **TOTAL** | 2 groups<br>6 schedules | N=60<br>[30 Ad5Ab neg.;<br>30 Ad5Ab pos.] | | DNA prime N= 30 (90 DNA vacs.)<br>rAd5 prime N=30 (30 rAd5 prime vacs) | | | 60 rAd5 boosts |

The hypotheses are: 1) IM, SC and ID are all safe routes of administration for both the DNA and rAd5 vaccines; 2) all regimens will elicit immune responses to HIV-1-specific peptides; 3) intradermal administration will allow a lower dosage of the DNA vaccine to be used for eliciting an immune response; and 4) rAd5 booster administered after a rAd5 prime will boost the cellular and humoral immune response. All subjects will receive a booster vaccination with $10^{10}$ PU rAd5 IM at Week 24. Safety of the vaccine regimens will be evaluated at scheduled study visits and by study subject report.

Specimens to evaluate immunogenicity will be taken at baseline and at specified time points. The HIV-1-specific immune responses will be assessed by cellular immune function assays and humoral immunity assays. The study subjects will require 42 weeks on study to complete the prime-boost regimen and follow-up.

### 4.1 STUDY POPULATION

All study activities will be carried out at the National Institutes of Health. Sixty healthy, HIV-negative volunteers will be recruited through IRB-approved advertising and will be screened through VRC 000 (02-I-0127), a screening protocol for healthy volunteers who are interested in participating in HIV vaccine clinical trials, to confirm eligibility requirements for participation. The screening and education process required prior to enrollment should ensure that subjects comprehend the purpose and details of the study. This Phase I study to establish safety of the prime-boost vaccination schedules in healthy individuals will be limited to adults who are 18-50 years old at the time of enrollment.

Prior to signing the VRC 011 informed consent, eligible volunteers will take a short “Assessment of Understanding” quiz to test understanding of this vaccine study. Incorrect answers will be explained to the volunteer and they will sign the informed consent document only after the study coordinator is satisfied with their understanding of the study.

#### 4.1.1 Inclusion Criteria

***A participant must meet all of the following criteria:***

1. 18 to 50 years old.
2. Available for clinical follow-up through Week 42 of the study.
3. Able to provide proof of identity to the satisfaction of the study clinician completing the enrollment process.
4. Complete an Assessment of Understanding prior to enrollment and verbalize understanding of all questions answered incorrectly.
5. Able and willing to complete the informed consent process.
6. Willing to receive HIV test results and willing to abide by NIH guidelines for partner notification of positive HIV results.
7. Willing to donate blood for sample storage to be used for future research.
8. Willing to discuss HIV infection risks and amenable to risk reduction counseling.
9. In good general health without clinically significant medical history.
10. Physical examination and laboratory results without clinically significant findings and a body mass index (BMI) less than 40 within the 28 days prior to enrollment.

***Laboratory Criteria within 28 days prior to enrollment:***

11. Hemoglobin $\geq$ 11.5 g/dL for women; $\geq$13.5 g/dL for men.

12. White blood cells (WBC) = 3,300-12,000 cells/$mm^3$.

13. Differential either within institutional normal range or accompanied by site physician approval.

14. Total lymphocyte count ≥ 800 cells/$mm^3$.

15. Platelets = 125,000 – 550,000/$mm^3$.
16. Alanine aminotransferase (ALT) ≤ 1.25 x upper limit of normal.

17. Serum creatinine ≤ upper limit of normal.

18. Normal urinalysis defined as negative glucose, negative or trace protein, and no clinically significant blood in the urine.

19. Negative Food and Drug Administration (FDA)-approved HIV blood test.

20. Negative hepatitis B surface antigen.

21. Negative anti-HCV (hepatitis C virus antibody) and negative HCV PCR.

***Female-Specific Criteria:***

22. Negative β-HCG (human chorionic gonadotropin) pregnancy test (urine or serum) on day of enrollment for women presumed to be of reproductive potential.

23. A female participant must meet any of the following criteria:

    No reproductive potential because of menopause [one year without menses] or because of a hysterectomy, bilateral oophorectomy, or tubal ligation,

    or

    Participant agrees to be heterosexually inactive at least 21 days prior to enrollment and through Week 42 of the study,

    or

    Participant agrees to consistently practice contraception at least 21 days prior to enrollment and through Week 42 of the study by one of the following methods:
    - condoms, male or female, with or without a spermicide
    - diaphragm or cervical cap with spermicide
    - intrauterine device
    - contraceptive pills or patch, Norplant, Depo-Provera or other FDA-approved contraceptive method
    - male partner has previously undergone a vasectomy.

#### 4.1.2 Exclusion Criteria

***A volunteer will be excluded if one or more of the following conditions apply***:

***Women:***

1. Woman who is breast-feeding or planning to become pregnant during the 42 weeks of study participation.

***Volunteer has received any of the following substances:***

2. HIV vaccine in a prior clinical trial.
3. Immunosuppressive medications or cytotoxic medications or inhaled corticosteroids within the past 3 months (with the exception of corticosteroid nasal spray for allergic rhinitis; topical corticosteroids for an acute, uncomplicated dermatitis, short-acting beta-agonists in controlled asthmatics; or a course of corticosteroids that was 10 days or fewer in duration that was completed at least 2 weeks prior to study enrollment for a non-chronic condition).
4. Blood products within 120 days prior to HIV screening.
5. Immunoglobulin within 60 days prior to HIV screening.
6. Investigational research agents within 30 days prior to initial study vaccine administration.
7. Live attenuated vaccines within 30 days prior to initial study vaccine administration.
8. Medically indicated subunit or killed vaccines, e.g. influenza, pneumococcal, or allergy treatment with antigen injections, within 14 days of study vaccine administration.
9. Current anti-tuberculosis prophylaxis or therapy.

***Volunteer has a history of any of the following clinically significant conditions:***

10. Serious adverse reactions to vaccines such as anaphylaxis, hives, respiratory difficulty, angioedema, or abdominal pain.
11. Autoimmune disease or immunodeficiency.
12. Asthma that is unstable or required emergent care, urgent care, hospitalization or intubation during the past two years or that requires the use of oral or intravenous corticosteroids.
13. Diabetes mellitus (type I or II), with the exception of gestational diabetes.
14. History of thyroidectomy or thyroid disease that required medication within the past 12 months.
15. Serious angioedema episodes within the previous 3 years or requiring medication in the previous two years.
16. Hypertension that is not well controlled by medication or is more than 145/95 at enrollment.

17. Bleeding disorder diagnosed by a doctor (e.g. factor deficiency, coagulopathy, or platelet disorder requiring special precautions) or significant bruising or bleeding difficulties with IM injections or blood draws.

18. Syphilis infection that is active or a positive serology due to a syphilis infection treated less than six months ago.

19. Malignancy that is active or treated malignancy for which there is not *reasonable* assurance of sustained cure or malignancy that is likely to recur during the period of the study.

20. Seizure disorder other than: 1) febrile seizures under the age of two, 2) seizures secondary to alcohol withdrawal more than 3 years ago, or 3) a singular seizure not requiring treatment within the last 3 years.

21. Asplenia, functional asplenia or any condition resulting in the absence or removal of the spleen.

22. Psychiatric condition that precludes compliance with the protocol; past or present psychoses; past or present bipolar disorder; disorder requiring lithium; or within five years prior to enrollment, history of a suicide plan or attempt.

23. Any medical, psychiatric, social condition, occupational reason or other responsibility that, in the judgment of the investigator, is a contraindication to protocol participation or impairs a volunteer's ability to give informed consent.

24. A subject with 3 or more of the 5 health risk factors noted below will be excluded:
    · Current smoker (or quit smoking less than 28 days prior to enrollment)
    · BMI >35
    · Fasting low density lipoprotein (LDL) > 159 mg/dL <u>or</u> fasting cholesterol >239 mg/dL
    · Systolic blood pressure >140 mm Hg or diastolic blood pressure >90 mm Hg
    · Fasting blood glucose >125 mg/dL
    
    <u>Note</u>: The fasting blood tests require 8 hours fast prior to the blood draw. The results used for eligibility screening must be from tests completed no more than 12 weeks (84 days) prior to day of enrollment. The individual criteria for BMI (inclusion item 10) and blood pressure (exclusion item 16) must also be met.

### 4.2 SCHEDULE OF CLINICAL PROCEDURES AND LABORATORY ASSAYS

Evaluation of the safety of this vaccine will include laboratory studies, medical history, physical assessment by clinicians, and subject self-assessment recorded on a diary card. Potential adverse reactions will be further evaluated prior to continuing the immunization schedule. Blood tests for immune responses will be performed at the Vaccine Research Center. The study schedule is described in Section 4.2.2 and presented in the form of a Table in Appendix III. Total blood volume drawn from each subject will not exceed the NIH Clinical Center Guidelines of 450 mL in any 6-week period.

#### 4.2.1 Screening

Screening for this study will be completed through the Vaccine Research Center's Screening Protocol, VRC 000 (NIH 02-I-0127). The evaluations and sample collection that will be included in VRC 000 screening are a medical history, physical exam, complete blood count with differential, prothrombin time (PT), partial thromboplastin time (PTT), chemistry panel (including fasting glucose, cholesterol and LDL), quantitative immunoglobulins, rapid plasma reagin (RPR), hepatitis B surface antigen, anti-hepatitis C antibody, HCV PCR, anti-dsDNA, HIV ELISA/Western Blot, HIV PCR, T-cell subsets, adenovirus serology, urinalysis, pregnancy test (for females of reproductive potential), and questions regarding sexual behavior and other practices. The adenovirus serology, fasting glucose, cholesterol and LDL used in the eligibility screening must be obtained within 12 weeks (84 days) prior to enrollment. Any test that has a specific eligibility requirement must be done within the window needed to meet study eligibility. Risk status for HIV infection will be determined by a series of questions designed to identify risk factors. Storage samples of peripheral blood mononuclear cells (PBMCs) and serum will also be collected. General eligibility for clinical trials will be dependent on results of laboratory tests and answers to the interview questions. Informed consent documents for vaccine trials will be reviewed, and counseling relating to the potential risks of becoming pregnant during this trial and avoiding HIV infection will be provided. An Assessment of Understanding of VRC 011 is completed on the day the subject is scheduled to enroll in VRC 011.

#### 4.2.2 Day 0 through Week 42 Clinical Follow-Up and Week 94 Follow-up

Day 0 is defined as the day of VRC 011 enrollment and first injection. VRC 011-specific eligibility is reviewed on Day 0 as part of the enrollment process. Pregnancy test results for women of childbearing potential must be confirmed as negative prior to enrollment on Day 0 and also on each injection day prior to the study vaccination. Subjects will begin their randomized prime-boost schedule on the day of enrollment with the first vaccination. Day 0 evaluations prior to the first vaccination are the baseline for subsequent safety assessments. Refer to the itemized list in this section and the table in Appendix III for details on when each type of evaluation must be completed.

All vaccinations will be administered according to the randomization assignment to one of the six schedules. Neither clinic staff nor subjects will know in advance the group or schedule to be assigned to the sequential enrollees in the study. The Protocol Statistician will prepare the randomization plan and provide it to the Site Pharmacy and Data Management Center in advance so that the pharmacy database and study randomization confirmation screens can be set up prior to opening the study to accrual. However, the group and schedule assignment will become known to both clinic staff and the subject immediately after completing the subject's electronic enrollment for the study on Day 0. After the enrollment is accepted by the database, the study identification number prints out along with the group and schedule assignment. Enrollment on Day 0 is followed on the same day by the first study injection.

**Vaccination schedule for Group 1 (DNA prime-rAd5 boost)**: Day 0, Day 28 ± 7 and Day 56 ± 7 (with at least 21 days between injections). The rAd5 booster vaccination will be scheduled as close to Day 168 as possible (with a -7 days to +14 days window permitted for scheduling).

**Vaccination schedule for Group 2 (rAd5 prime-rAd5 boost)**: Day 0 for the prime vaccination and as close to Day 168 as possible for the rAd5 booster vaccination (with a -7 days to +14 days

window permitted for scheduling).

All vaccinations will be administered with a needle and syringe using an appropriate technique to ensure IM, SC or ID administration according to the randomized schedule of the given subject. The IM injections will be given in the deltoid muscle. The SC injections will be given in the triceps area of the upper arm. The ID injections will be administered in the skin overlying the deltoid area of the arm. Left and right arms will usually be alternated for the sequential DNA injections unless there is a medical indication (*e.g.*, an arm injury or local skin problem) or significant tattoo that would preclude injecting or evaluating the arm that is next in the alternating sequence. Subjects may choose to have the booster vaccination in the non-dominant arm even if it is not next in the alternating sequence because the booster vaccination is sometimes associated with significant local reactogenicity and because there is a 4-6 month interval between the prime(s) and the boost.

**Administration of DNA or rAd5 vaccines by IM injection**: The clinician administering the injection will select a 23-gauge needle, with a length of 1 or 1.5 inch (depending on subject arm size) and use standard IM injection technique in order to ensure IM injection. This method applies to the three prime DNA vaccine injections for Groups 1A and 1B, the prime rAd5 injections for Groups 2A and 2B and all booster injections of the rAd5 vaccine for all Groups.

**Administration of DNA or rAd5 vaccines by SC injection**: The clinician administering the injection will select a 23-gauge needle, with a length of 1/2 inch and use standard SC injection technique in order to ensure SC injection. This method applies to the three prime DNA vaccine injections for Groups 1C and 1D and the prime rAd5 injections for Groups 2C and 2D.

**Administration of DNA or rAd5 vaccine by ID injection**: The clinician administering the injection will select a 25-gauge needle, with a length of 5/8 inch. The needle will be inserted into the skin at a 15 degree angle to the skin and bevel side up until the bevel is seen to be fully under the skin. The needle bevel will then be rotated about 45 degrees. The syringe contents will be injected to form a small bleb. Rotating the bevel is intended to ensure that the needle bevel is fully under the skin and to reduce the chance of inadvertent leaking of the injectate during the injection. This method applies to the three prime DNA vaccine injections for Groups 1E and 1F and the prime rAd5 injections for Groups 2E and 2F.

Each used needle and syringe is disposed of in the medical waste sharps container and the sharps container is disposed of in the medical pathology waste (MPW) container for incineration.

Following each study injection (DNA vaccine or rAd5 vaccine), subjects will be observed for a minimum of 30 minutes. Vital signs (temperature, blood pressure, pulse and respiratory rate) will be completed between 30 and 45 minutes post-immunization and the injection site will be inspected for evidence of local reaction. Subjects will be given a “Diary Card” on which to record temperature and symptoms daily for 5 days.

**Group 1 Follow-up Schedule for DNA Prime vaccinations**: Subjects in Group 1 will have a clinic visit at 3 ±1 days following each of the three DNA injections. These visits will include interim history, vital signs, lymph node exam, and examination of the vaccination site. Erythema, induration or skin lesions will be documented by measurement of perpendicular diameters. A photograph of the vaccination site will be taken if there are any clinical findings. If there is evidence of a skin lesion, the visit at 7 ±1 days after vaccination will also be a clinic

visit; otherwise follow-up at this timepoint will be first by telephone with a clinic visit scheduled if the subject reports changes suggesting a skin lesion may be forming. When photos are taken, clinic staff will attempt to photograph the vaccination site at the same distance each time and with similar lighting conditions. A tag with metric ruler, subject number, date, and visit identifiers will be placed below the vaccination site prior to photographing the site. The Diary Card will be collected at the first clinic visit after each DNA vaccination when it is complete.

At 14 ±3 days after each DNA injection, study subjects will be evaluated at a clinic visit that includes interim history, vital signs, lymph node exam, examination of the vaccination site, and collection of urine and blood samples. A photograph will be taken if there is a skin lesion or need to document resolution of any findings that were present at a prior visit.

The Group 1 DNA prime follow-up schedule also includes a visit at Week 12 (±3 days) and includes interim history, vital signs, lymph node exam, and collection of blood samples for research immunology. Research immunology samples will also be collected at the Week 8 (±7 days) visit prior to administration of the DNA vaccination.

**Group 2 Follow-up Schedule for rAd5 PRIME vaccination**: Subjects in Group 2 will have a clinic visit at 3 ±1 days following the rAd5 prime injection. These visits will include interim history, vital signs, lymph node exam, and examination of the vaccination site. Erythema, induration or skin lesions will be documented by measurement of perpendicular diameters. A photograph of the vaccination site will be taken if there are any clinical findings. If there is evidence of a skin lesion, the visit at 7 ±1 days after vaccination will also be a clinic visit; otherwise follow-up at this timepoint will be first by telephone with a clinic visit scheduled if the subject reports changes suggesting a skin lesion may be forming. When photos are taken, clinic staff will attempt to photograph the vaccination site at the same distance each time and with similar lighting conditions. A tag with metric ruler, subject number, date, and visit identifiers will be placed below the vaccination site prior to photographing the site. The Diary Card will be collected at the first clinic visit after each DNA vaccination when it is complete.

At 14 ±3 days after the rAd5 prime injection, study subjects will be evaluated at a clinic visit that includes interim history, vital signs, lymph node exam, examination of the vaccination site, and collection of urine and blood samples. A photograph will be taken if there is a skin lesion or need to document resolution of any findings that were present at a prior visit.

The Group 2 rAd5 prime follow-up schedule also includes visits at Week 4 (±3 days) and Week 12 (±3 days) and include interim history, vital signs, lymph node exam, and collection of blood samples for research immunology.

**Group 1 and Group 2 Follow-up Schedule for rAd5 Booster vaccinations**: The rAd5 vaccinations are administered to both Groups on all schedules at Week 24 (-7 days to +14 days). The first follow-up for any rAd5 booster vaccination will be performed by telephone on the first or second day afterwards. A clinic visit will occur within 24 hours if indicated by the telephone interview. Events reported in the telephone interview that will require a clinic visit include rash, urticaria (hives), fever of 38.7˚C (Grade 2) or higher that does not resolve within 24 hours, or significant impairment in the activities of daily living (ADL). At 14 ±3 days after the rAd5 booster vaccination (i.e., the "Week 26" visit), study subjects will be evaluated at a clinic visit. This visit will include interim history, vital signs, lymph node exam and examination of the vaccination site. The 5-day Diary Card will be collected.

For both Groups the schedule of booster injection follow-up visits is Week 28 (±7 days), Week 30 (±7 days) and Week 42 (±14 days). These visits will include interim history, vital signs, lymph node exam, and collection of blood samples for research immunology. Note that if the rAd5 booster injection is delayed to Days 176 through 182 then the Week 28 visit may be adjusted further, if needed to keep the interval after the rAd5 injection close to 4 weeks post injection, respectively. The schedule of follow-up visits is shown in the table in Appendix III. Subjects are followed in the clinic until Week 42 (±14 days). This will be about 18 weeks of follow-up after the rAd5 booster vaccine.

**Optional Skin Biopsy**: Following a vaccine injection, a skin biopsy is an optional assessment if a skin lesion appears to be forming. Although not previously observed with these vaccines, it is theoretically possible that a reaction can occur at a prior vaccination site following a subsequent vaccination; such reactions may warrant a skin biopsy also. Skin biopsies are included in this study for research purposes to better understand the etiology (i.e., distinguish between immune, allergic, infectious or foreign body reaction) of skin lesions sometimes associated with vaccinations. Subjects may refuse to have a skin biopsy. The preferred timepoint for obtaining a skin biopsy is at the earliest visit after a vaccination where there is evidence of a skin lesion. This is expected to be 3 ±1 days post-vaccination in most cases, but may be earlier or later depending upon individual circumstances. Subjects will not be asked to have more than two skin biopsies of vaccination site skin lesions solely for research purposes. However, if a skin biopsy is recommended for clinical care purposes, additional skin biopsies may be done with subject consent. A standard procedure consent will be obtained prior to obtaining a skin biopsy.

At intervals throughout the study subjects will have blood drawn for immunologic assays. Any cells, serum or plasma not used will be stored for future virological and immunological assays. Subjects will also be interviewed at the final clinical visit regarding social harms, including problems with employment, travel, immigration, access to insurance, medical or dental care, and negative reactions from family, friends, and co-workers. Study visit procedures and tests through last clinic visit are as follows:

- "VRC 011 Assessment of Understanding" Quiz (Day 0)
- Signature of study participation informed consent form for VRC 011 (Day 0)
- Clinical evaluations: vital signs and weight (every visit day); axillary lymph node exam (vaccination visits and follow-up visits through 4 weeks after each vaccination); targeted physical exam on any visit if indicated by interim complaints or laboratory findings.
- Interim medical history (every visit).
- Counseling on HIV and avoidance of pregnancy (Day 0; offered every subsequent visit)
- Study vaccinations; schedule varied by randomized assignment (refer to study schema).
- Post-injection vital signs and assessment of injection site at 30 to 45 minutes after a study vaccination.
- Diary Card: Baseline on day of vaccination; 5-day diary card for self-assessment by subject following each vaccination. The diary card will include the parameters: unusually tired/feeling unwell, muscles aches (at other than injection site), headache, chills, nausea, and pain/tenderness at injection site. Subjects will also record highest measured temperature,

measurement of perpendicular diameters for redness and swelling at injection site and note if there is evidence of a skin lesion at the vaccination site. The diary cards are collected at the earliest clinic visit after each injection when the card is complete.

- Photograph of DNA vaccination site if there are any skin lesion or other significant clinical findings at the vaccination site.
- DNA vaccination site skin biopsy: optional assessment of DNA vaccination sites with skin lesion. Limited to two per subject unless medically indicated for clinical care purposes.
- Serum or urine pregnancy test, for females of reproductive potential (vaccination visits and last clinic visit).
- HLA (human leukocyte antigen) type: blood sample collected Week 2 for convenience, but may be done from a sample collected at any timepoint, if needed.
- CBC, differential, platelet count: Day 0 and Weeks 2, 4, 12, 24, 26, 28, 30 and 42 for all subjects; also Weeks 6, 8 and 10 for subjects on DNA prime schedules.
- Creatinine and ALT: Day 0 and Weeks 2, 4, 12, 24, 26, 28, 30 and 42 for all subjects; also Weeks 6, 8 and 10 for subjects on DNA prime schedules.
- Urinalysis: Day 0 and Weeks 2, 4, 24 and 26 for all subjects; also Weeks 6, 8 and 10 for subjects on DNA prime schedules.
- T cell FACS (fluorescence-activated cell sorter) for CD4/CD8 (Day 0 and Weeks 12, 24, 30 and 42) for all subjects.
- HIV testing: ELISA (also Western blot if ELISA is positive) and HIV PCR (Day 0 and Weeks 12, 24, 30 and 42) for all subjects.
- HIV specific antibody research assays: Day 0 and Weeks 8, 12, 24, 28 and 42 for subjects on DNA prime schedule; Day 0 and Weeks 4, 12, 24, 28 and 42 for subjects on rAd5 prime schedules. Note: The assays will not be performed immediately, but rather completed at a later date using frozen samples. Additional timepoints using stored sera may be performed if of interest.
- ELISpot and Intracellular cytokine staining (ICS) assays: Day 0 and Weeks 6, 8, 10, 12, 24, 28, 30 and 42 for subjects on DNA prime schedules; Day 0 and Weeks 4, 12, 24, 28, 30 and 42 for subjects on rAd5 prime schedules. Note: The assays will not be performed immediately, but rather completed at a later date using frozen samples. PBMC and plasma for storage will be saved from the blood collected for these assays. Other immunological assays, such as multiparameter flow cytometry may also be performed from stored samples.
- Social Impact Questionnaire (Week 42). The Social Impact questionnaire will include parameters: personal relationships, travel or immigration, employment, education, medical or dental, health insurance, life insurance, housing, military/other government agency and other.
- Serum for archiving: Day 0 and Weeks 4, 12, 24, 28, 30 and 42 for all subjects; also at Weeks 6, 8 and 10 for subjects on DNA prime schedules.
- Adenovirus Serology: Day 0 and Weeks 24, 28 and 42 for all subjects; also at Week 4 for

subjects on rAd5 prime schedules. This assay may be done from the serum for archiving and does not require a separate blood collection.

Week 94 Long-term Follow-up:

After the Week 42 clinic visit, subjects will be contacted one year later. Subjects will be encouraged to return for a clinic visit at Week 94 (± 28 days) to be interviewed about any interval life-threatening adverse events, persistent or significant disability/incapacity, non-elective hospitalizations, new chronic diseases requiring ongoing medical management or medication or outcomes of any pregnancies (including if there were any congenital anomalies/birth defects), as well as to have HIV testing (ELISA with Western blot if positive and HIV PCR) and for a research immunology blood draw (PBMC, plasma and serum). A subject may opt to be contacted only by telephone, mail or e-mail to allow collection of the interview information specified above without a follow-up blood draw. If there are any subject deaths in the interval between Week 42 and Week 94, an attempt will be made to obtain information about the cause of death. Follow-up testing for vaccine-induced HIV antibody is permitted for up to 5 years after the Week 42 visit (see Section 4.3). Subjects may also be contacted at other times to confirm contact information and provide notification of release of study results.

### 4.3 MONITORING FOR HIV INFECTION

It is possible that this vaccination regimen will induce immunologic responses that are detected by standard HIV screening techniques, even though the vaccines will not cause HIV infection. The following steps will be taken to ensure detection of HIV infection and to protect participants from adverse consequences associated with an HIV antibody test that indicates an antibody response to the vaccine:

- Study participants will receive regularly scheduled counseling regarding avoidance of HIV infection in accordance with the most recent CDC HIV Counseling Guidelines.

- Study participants will be screened for HIV infection periodically while participating in the study (see Appendix III for schedule of testing).

- If there is any clinical or laboratory indication of HIV infection, any test required to make a definitive diagnosis, including Western blot analysis, viral load measurement (PCR), or other tests will be performed.

- Confirming tests will be performed as soon as possible once a positive antibody response is identified. Participants will be promptly informed if they are HIV-infected. Participants who are found to have vaccine-induced antibody responses, but with no evidence of HIV infection, will be informed that they are not HIV-infected. Written documentation describing any vaccine-induced antibody response and confirming data will be provided when the study is completed. This should be sufficient evidence that the antibody response as of the date of testing resulted from vaccination and not from naturally occurring infection. Participants with vaccine-induced antibody will be provided with the opportunity for HIV antibody testing annually for five years to monitor their serological status. If vaccine-induced antibody persists beyond five years, testing may be performed through a VRC sample collection protocol. Participants will be counseled regarding the potential for antibody responses and the implications of such responses prior to participation in the study.

## 4.4 INTERCURRENT HIV INFECTION

The vaccines cannot cause HIV infection. Subjects who become HIV infected due to other causes while participating in the study will be referred for their medical care and treatment and management of the disease. They may be given the opportunity to enroll in an appropriate study of acute HIV infection or a long-term follow-up study, if one is available. The NIH investigators will not be responsible for providing ongoing medical care or antiretroviral medications in the event of HIV-1 infection.

## 4.5 CONCOMITANT MEDICATIONS

Concomitant medications are recorded at screening and every study visit. The concomitant medications eligibility criteria for enrollment continue to apply for the subject to remain eligible for each study injection. If an enrolled subject develops the need for a medication that is prohibited by the eligibility criteria, then further study injections will be discontinued. If an FDA-approved live attenuated vaccine is required during the study vaccination schedule for an immediate medical need, then study injections must be discontinued if it cannot be administered with at least 14 days after the previous study vaccination and 30 days before the next study vaccination. If an FDA-approved subunit or killed vaccine is required for an immediate medical need, then it must be given at least 14 days before or 14 days after any study injection for the subject to remain eligible for additional study injections. If it will not imperil a subject’s health, FDA-approved vaccines should be deferred until at least 30 days after the final study injection. Any subject who receives at least one study injection will continue with the clinical and laboratory evaluations specified by the study through the 12 months of follow-up.

## 4.6 CRITERIA FOR WITHDRAWAL OF A SUBJECT FROM INJECTION SCHEDULE

Under certain circumstances, a subject will be terminated from participating in further injections. Participants who are discontinued from additional study vaccinations will continue to be followed according to the schedule of safety and immunogenicity evaluations, except that the follow-up evaluations that are specifically for safety follow-up on a vaccination do not need to be completed when a vaccination is not given. Referring to Appendix III, these are the “A”, “B” and “C” visits that follow a DNA vaccination and the “A” and “B” visits that follow the rAd5 vaccination. Specific events that will require withdrawal of a subject from the vaccination schedule include:

1. HIV infection;
2. Pregnancy;
3. Grade 2 adverse event classified as <u>possibly</u> associated with immunization that does not resolve to baseline in time for the next scheduled immunization;
4. Grade 2 adverse event classified as <u>probably or definitely</u> associated with immunization (with the exception of grade 2 fever, pain/tenderness, fatigue/malaise, nausea, headache, chills or myalgia);
5. Grade 3 or 4 systemic or injection site adverse event classified as possibly, probably or definitely associated with immunization;
6. Type 1 hypersensitivity associated with immunization;

7. An intercurrent illness that is not expected to resolve prior to the next scheduled immunization and is judged by the Principal Investigator (or designee) to require discontinuation;
8. Treatment with systemic glucocorticoids (e.g., prednisone or other glucocorticoid) or other immunomodulators (other than NSAIDs) for any reason (with the exception of a course of corticosteroids that was 10 days or fewer in duration for a non-chronic condition and completed at least 14 days prior to the "booster" study injection);
9. Medical need for concomitant vaccine during the period of study vaccinations that requires discontinuation from the study vaccination schedule (see section 4.5);
10. Repeated failure to comply with protocol requirements;
11. The IND sponsor, study sponsor or Principal Investigator decides to stop or cancel the study;
12. The IRB or the FDA request that the study be stopped.

### 4.7 CRITERIA FOR STOPPING STUDY

The Principal Investigator will closely monitor and analyze study data as they become available and will make determinations regarding the presence and severity of adverse events. The DAIDS Medical Officer will provide an independent review of adverse events that have a bearing on study pauses. In keeping with a recently modified (November 9, 2005) set of pause rules for the VRC 008 study, this protocol will not be paused for the signs and symptoms consistent with the self-limited fever and flu-like syndrome that may occur in the rAd5 post-vaccination period. The plan will be as follows:

Any Grade 2 or Grade 3 post-vaccination reactogenicity adverse events of pain/tenderness, fever, malaise, fatigue, headache, chills, nausea, myalgia, or arthralgia will be reviewed by the IND Sponsor's Medical Officer and the Principal Investigator at weekly safety monitoring review (see Section 8.9) while vaccinations are ongoing. From the initiation of the vaccinations through 4 weeks after the rAd5 booster vaccinations, a quarterly safety monitoring report will be submitted to the IND until all subjects have completed at least 4 weeks of follow-up for the rAd5 booster vaccination.

The administration of study injections and new enrollments will be paused and the IND sponsor will be promptly notified according to the criteria that follow. Counting of events of the types listed is done for each investigational vaccine separately:

**Grade 4 Pause Rules**:

- **One** (or more) subject experiences a Grade 4 adverse event that is assessed as possibly, probably or definitely related to a study vaccine;

**Grade 3 Pause Rules**:

- **One** (or more) subject experiences a Grade 3 adverse event assessed as possibly, probably or definitely related to a study vaccine: this criterion applies to erythema, induration, vomiting, laboratory abnormalities or other clinical adverse experiences, but does not apply to the local or systemic post-vaccination adverse events of pain/tenderness, fever, malaise, fatigue, headache, chills, nausea, myalgia, or arthralgia;

### Grade 2 Pause Rules:

- **One** (or more) subject experiences Grade 2 erythema or induration assessed as possibly, probably or definitely related to a study vaccine at an injection site;
  OR
- **Two** (or more) subjects experience the **same** Grade 2 or higher adverse event assessed as possibly, probably or definitely related to the same vaccine: this criterion applies to vomiting, laboratory abnormalities or other clinical adverse experiences, but does not apply the local or systemic post-vaccination adverse events of pain/tenderness, fever, malaise, fatigue, headache, chills, nausea, myalgia, or arthralgia.

**Plan for review of pauses and deciding about whether or not to resume**:

The study injections and enrollments would resume only if review of the adverse events that caused the pause resulted in a recommendation to permit further study injections and study enrollments. The reviews to make this decision will occur as follows:

Grade 2 events that meet the pause criteria: The IND Sponsor, in consultation with the Principal Investigator, will conduct the review and make the decision to resume or close the study for any Grade 2 events leading to a pause.

Grade 3 or Grade 4 events that meet the pause criteria: The IND Sponsor, with participation by the Principal Investigator, will consult with the FDA to conduct the review and make the decision to resume or close the study for any Grade 3 and Grade 4 adverse events that meet the criteria for pausing the study.

Safety data reports and changes in study status are submitted to the IRB promptly in accordance with Section 5.4 and institutional policy.

## 5. SAFETY AND ADVERSE EVENT REPORTING

### 5.1 ADVERSE EVENTS

An adverse event is any unfavorable or unintended change in body structure, body function or laboratory result associated temporally with the use of study treatment, whether or not considered related to the study treatment. Each adverse event will be graded according to the Table for Grading Severity of Adverse Events (see Appendix IV).

### 5.2 SERIOUS ADVERSE EVENTS (SAE)

The term "Serious Adverse Drug Experience" is defined in 21 CFR 312.32 as follows: "Any adverse drug experience occurring at any dose that results in any of the following outcomes: Death, a life-threatening adverse drug experience, inpatient hospitalization or prolongation of existing hospitalization, a persistent or significant disability/incapacity, or a congenital anomaly/birth defect. Important medical events that may not result in death, be life-threatening, or require hospitalization may be considered a serious adverse drug experience when, based upon appropriate medical judgment, they may jeopardize the subject or require medical or surgical intervention to prevent one of the outcomes listed in this definition. Examples of such medical events include allergic bronchospasm requiring intensive treatment, blood dyscrasias or convulsions that do not result in inpatient hospitalization, or the development of drug

dependency or drug abuse."

In Section 5.3 the term "Expedited Adverse Event" (EAE) encompasses the events that would be considered an SAE by the 21 CFR 312.32 definition.

### 5.3 ADVERSE EVENT REPORTING TO THE IND SPONSOR

Information on adverse events (AEs) is collected by Study Nurses and other clinic staff and entered into a computer database. The Principal Investigator and the Study Coordinator review these data on an ongoing basis.

The expedited adverse event (EAE) reporting requirements and definitions for this study and the methods for expedited reporting of AEs to the DAIDS Regulatory Compliance Center (RCC) Safety Office are defined in "The Manual for Expedited Reporting of Adverse Events to DAIDS" (DAIDS EAE Manual) dated May 6, 2004. The DAIDS EAE Manual is available on the RCC website: http://rcc.tech-res-intl.com/.

AEs reported on an expedited basis must be documented on the DAIDS Expedited Adverse Event Reporting Form (EAE Reporting Form) available on the RCC website: http://rcc.tech-res-intl.com. RCC contact information is provided in Appendix II.

EAE Reporting Level:

This study uses the Standard Level of expedited AE reporting as defined in the DAIDS EAE Manual. Briefly summarized, Standard Level reporting requires completion of an EAE report form for the following types of AEs occurring after exposure to the study agent:

- Result in death regardless of relationship to study agent.
- Are congenital anomalies, birth defects, or fetal losses regardless of relationship to study agent.
- Result in persistent or significant disabilities or incapacities regardless of relationship to study agent.
- Are a suspected adverse drug reaction (i.e., definitely, probably, possibly, or probably not related to study agent) that requires hospitalization, or prolongs existing hospitalization OR requires intervention to prevent significant/permanent disability or death.
- Are life-threatening (including all Grade 4 adverse events) suspected adverse drug reactions (i.e., assessed as definitely, probably, possibly or probably not related to study agent).

In addition, any event, regardless of grade, which in the judgment of a site investigator represents a serious adverse event, may be reported to the IND sponsor as an expedited report.

EAE Reporting Period:

AEs must be reported on an expedited basis at the Standard Level during the protocol-defined EAE Reporting Period, which for this study is from study enrollment until the last required clinical visit at Week 42 or until discontinuation of the subject from study participation for any reason.

After the end of the protocol-defined EAE reporting period stated above, the site must report serious, unexpected, clinical suspected adverse vaccine reactions if the study site staff becomes

aware of the event on a passive basis, i.e. from publicly available information.

Study Agents for Expedited Reporting to DAIDS:

The study agents that must be considered when determining relationships of AEs requiring expedited reporting to DAIDS are: VRC-HIVDNA016-00-VP and VRC-HIVADV014-00-VP.

Grading Severity of Events:

The Table for Grading the Severity of Adult Adverse Events is: "The Division of AIDS Table for Grading the Severity of Adult and Pediatric Adverse Events, Version 1.0, Dec 2004" (see Appendix IV).

The EAE report must be reported on the EAE form and submitted by the clinical site to the IND sponsor (DAIDS) through the Regulatory Compliance Center (RCC) Safety Office (RCCSafetyOffice@tech-res.com) as soon as possible, but no later than 3 working days after the clinical site becomes aware of events meeting these criteria. The IND sponsor is responsible for submitting IND safety reports to the FDA, as necessary, per 21 CFR 312.32. DAIDS submits IND safety reports as soon as possible, but no later than 15 days after initial receipt of the information.

## 5.4 Adverse Event Reporting to the Institutional Review Board

Adverse event reporting requirements to the NIAID Institutional Review Board (IRB) for this protocol are as follows:

- Investigators will submit a completed serious adverse event report to the NIAID IRB within 7 days after becoming aware of a subject death, a potentially life-threatening (grade 4) serious adverse event that is possibly, probably or definitely related to investigational agent, an inpatient hospitalization (other than elective), a persistent or significant disability/incapacity, or a congenital anomaly/birth defect.

- Investigators will submit a completed serious adverse event report to the NIAID IRB within 15 days after becoming aware of any Grade 3 (severe) adverse event that is possibly, probably or definitely related to investigational agent.

- Investigators will report within 15 days on any other event or condition regardless of grade, which in their judgment represents an event reportable to the IRB.

- Investigators will forward all IND safety reports and related FDA communications to the IRB within 15 days of receipt.

- A summary of all adverse events will be reported to the NIAID IRB with submission of a request for continuing review.

## 5.5 Serious Adverse Event Reporting to the Institutional Biosafety Committee

The Institutional Biosafety Committee (IBC) has a responsibility to review research using recombinant DNA for compliance with NIH Guidelines. In keeping with IBC requirements, any SAE reports sent to the IRB will be provided to the IBC at the same time.

# 6. STATISTICAL CONSIDERATIONS

## 6.1 Overview

This study is a single-center, randomized trial to assess the safety and tolerability of intramuscular, intradermal and subcutaneous routes of administration of two HIV vaccines in HIV-uninfected adults. A preliminary assessment of immunogenicity will also be performed.

## 6.2 Objectives

The primary objective is to evaluate the safety and tolerability in humans of the vaccination regimens. Secondary objectives include evaluating the immunogenicity of the vaccination regimens, the development of adenovirus serotype 5 neutralizing antibody and the social impact of participating in an HIV-1 vaccine trial. The study will provide preliminary information on whether the IM, SC and ID routes of administration are similar in safety and immunogenicity and whether pre-enrollment adenovirus serotype 5 antibody (Ad5Ab) titer affects safety of or immune response to the rAd5 vaccination.

## 6.3 Endpoints

### 6.3.1 Safety

Assessment of product safety will include clinical observation and monitoring of hematological and chemical parameters. Safety will be closely monitored after injection and evaluated through 18 weeks following the adenoviral vector booster vaccination. See Section 4.2 and Appendix III for details and specified time points. The following parameters will be assessed:

- Local reactogenicity signs and symptoms
- Systemic reactogenicity signs and symptoms
- Laboratory measures of safety
- Adverse and serious adverse experiences

### 6.3.2 Immunogenicity

The principal immunogenicity endpoints for cellular immune responses are measured at Week 0 (baseline), 4 weeks after third DNA vaccination (in Group 1), 4 weeks after the rAd5 prime vaccination (Group 2) and 4 weeks after rAd5 vaccine booster in both Groups. They will consist of HIV-1-specific T cell responses, as measured by ELISpot and intracellular cytokine staining (ICS) assays and by research ELISA for vaccine-specific antigens. These and other immunogenicity assays will be performed at other study timepoints as exploratory evaluations.

### 6.3.3 Social Impacts

Social impact variables, as measured by questionnaire at the last clinic visit, include any negative experiences or problems the participant experienced due to his/her participation in this study. The following social impacts will be followed during the course of the study: personal relationships, travel or immigration, employment, education, medical or dental care, health insurance, life insurance, housing, military/other government agency and other impacts identified by a participant.

### 6.4 SAMPLE SIZE AND ACCRUAL

Recruitment will target 60 healthy, HIV-uninfected adult participants between age 18 and 50 years old. The required clinical follow-up is through Study Week 42. Sample size will be 30 for subjects with safety data for the DNA prime-rAd5 boost regimens and 30 for the rAd5 prime-rAd5 boost regimens. For other evaluations, using a factorial design, the following samples sizes apply:

IM DNA vaccine primes: N = 10
SC DNA vaccine primes: N=10
ID DNA vaccine primes: N=10
IM rAd5 primes: N=10
SC rAd5 primes: N=10
ID rAd5 primes: N=10

#### 6.4.1 Randomization of Treatment Assignments

The randomization sequence will be obtained by computer-generated random numbers and provided to the study pharmacist by the statistician. Equal numbers of subjects with no pre-existing Ad5Ab titer (<1:12) and subjects with positive Ad5Ab titer (≥1:12) will be enrolled and equally randomized to each study group and schedule. Study numbers 01011001 through 01011030 will be used for the randomization of subjects with no pre-existing Ad5Ab titer (<1:12) to all regimens and study numbers 01011031 through 01011060 will be used for the randomization of subjects with positive Ad5Ab titer (≥1:12) to all regimens; within each stratum study numbers have been randomly assigned for the route of administration each subject will receive for the prime vaccination(s). Subjects with both high and low Ad5Ab titer will be enrolled simultaneously. The pharmacist, data management center and the statistician are responsible for maintaining security of the treatment assignments. To maintain blinding, any discussion of the treatment assignment between the VRC clinicians and the pharmacy staff or data management personnel is prohibited until after the assignments are permitted to be known to all.

To decrease the potential for participant dropouts during the period between randomization and initial vaccination, randomization will occur on Day 0 after the study consent is signed and eligibility is confirmed. The study number is assigned through completion of the eligibility checklist in the electronic study database and will be the next sequential number in the study number sequence for the subject's Ad5Ab titer category. The assignment to a study schedule and a route of administration for the prime vaccination(s) will become known to subjects and protocol staff shortly after study enrollment, as this will be included in the confirmation of study ID printed out from the study database.

#### 6.4.2 Power Calculations for Safety

The goal of the safety evaluation for this study is to identify safety concerns associated with injection. Sample size calculations for safety are expressed in terms of the ability to detect serious adverse experiences.

The ability of the study to identify serious adverse experiences is best expressed by the maximum true rate of events that would be unlikely to be observed and the minimum true rate of events that would very likely be observed. Specifically, there is a 90% chance of observing at least 1 serious adverse experience in the 60 volunteers if the true rate of such an event is at least

0.040; there is a 90% chance that we would not observe at least 1 serious adverse experience if the true rate is less than 0.002. Within any of the arms, there is a 90% chance of observing at least 1 serious adverse experience in the 10 volunteers if the true rate of such an event is at least 0.35; there is a 90% chance that we would not observe at least 1 serious adverse experience if the true rate is less than 0.010. Probabilities of observing 0 or 2 or more serious adverse experiences among the total sample size (N=60), within each baseline titer group (N=30), and within each treatment arm (N=10) are presented in Table 6.1 for a range of possible true event rates. These calculations provide a more complete picture of the sensitivity of this study design to identify potential safety problems with the vaccine.

**Table 6-1: Probability of response for different safety and immunogenicity scenarios**

| True Event rate | Pr(0/60) | Pr(2+/60) | Pr(0/30) | Pr(2+/30) | Pr(0/10) | Pr(2+/10) |
|---|---|---|---|---|---|---|
| 0.001 | 0.942 | 0.002 | 0.970 | <0.001 | 0.990 | <0.001 |
| 0.002 | 0.886 | 0.006 | 0.942 | 0.002 | 0.980 | <.001 |
| 0.003 | 0.835 | 0.014 | 0.914 | 0.004 | 0.970 | <0.001 |
| 0.005 | 0.740 | 0.037 | 0.860 | 0.010 | 0.951 | 0.001 |
| 0.010 | 0.547 | 0.121 | 0.740 | 0.036 | 0.904 | 0.004 |
| 0.030 | 0.161 | 0.541 | 0.401 | 0.227 | 0.737 | 0.035 |
| 0.040 | 0.086 | 0.698 | 0.294 | 0.339 | 0.665 | 0.058 |
| 0.050 | 0.046 | 0.808 | 0.215 | 0.446 | 0.599 | 0.086 |
| 0.075 | 0.009 | 0.945 | 0.096 | 0.669 | 0.459 | 0.170 |
| 0.100 | 0.002 | 0.986 | 0.042 | 0.816 | 0.349 | 0.264 |
| 0.150 | <0.001 | 0.999 | 0.008 | 0.952 | 0.197 | 0.456 |
| 0.200 | <0.001 | >0.999 | 0.001 | 0.989 | 0.107 | 0.624 |
| 0.250 | <0.001 | >0.999 | <.001 | 0.998 | 0.056 | 0.756 |
| 0.300 | <0.001 | >0.999 | <0.001 | >0.999 | 0.028 | 0.851 |
| 0.350 | <0.001 | >0.999 | <0.001 | >0.999 | 0.013 | 0.91 |

Table 6-2 gives the upper and lower bounds for 95% exact binomial confidence intervals for several possible numbers of events. For example, if none of the 60 participants receiving the vaccine experience serious adverse experiences to the vaccine, the 95% exact 2-sided upper confidence bound for the rate of such reactions in the population is 0.060. Within a group of 10, the confidence interval would range from 0 to 0.308.

**Table 6-2: 95% Confidence Intervals for all possible observed rates**

| | 95% CI | | 95% CI | | 95% CI |
|---|---|---|---|---|---|
| 0/60 | 0,0.060 | 0/30 | 0, 0.116 | 0/10 | 0,0.308 |
| 1/60 | 0,0.089 | 1/30 | 0.001, 0.172 | 1/10 | 0.003, 0.445 |
| 2/60 | 0.004, 0.115 | 2/30 | 0.008, 0.221 | 2/10 | 0.025, 0.556 |
| 3/60 | 0.010, 0.139 | 3/30 | 0.021, 0.265 | 3/10 | 0.067, 0.652 |
| 4/60 | 0.018, 0.162 | 4/30 | 0.038, 0.307 | 4/10 | 0.122, 0.738 |
| 5/60 | 0.028, 0.184 | 5/30 | 0.056, 0.347 | 5/10 | 0.187, 0.813 |
| 10/60 | 0.083, 0.285 | 10/30 | 0.173, 0.528 | 6/10 | 0.262, 0.878 |
| 20/60 | 0.217, 0.467 | 15/30 | 0.313, 0.687 | 7/10 | 0.348, 0.933 |

| | 95% CI | | 95% CI | | 95% CI |
|---|---|---|---|---|---|
| 30/60 | 0.368, 0.632 | 20/30 | 0.472, 0.827 | 8/10 | 0.44, 0.975 |
| 40/60 | 0.533, 0.783 | 25/30 | 0.653, 0.944 | 9/10 | 0.555, 0.997 |
| 50/60 | 0.715, 0.917 | 30/30 | 0.884, 1 | 10/10 | 0.692, 1 |
| 60/60 | 0.940, 1 | | | | |

### 6.4.3 Sample Size Calculations for Immunogenicity

The primary goal of this trial regarding immunogenicity outcomes is a preliminary estimation of response rates. The definition of response is based on comparing the percent of responding cells when stimulated to the background levels specific for each person at each time point. A statistical test is used to determine if the percent of responding cells is significantly higher than background; if so this is considered a response at this time point. A 1% false-positive rate is built into the statistical criteria and the methods were validated on both HIV-positive and HIV-negative samples. Table 6-2 is applicable to the immunogenic response rates, and gives the exact 95% confidence interval for possible numbers of responses among the different groups of volunteers. For example, if we observe 5 responses among the 30 vaccinees with no pre-existing Ad5Ab titer, our 95% exact binomial confidence interval for the true rate will range from 0.056 to 0.347

There is also interest in estimating the immunological response among the vaccinees who receive each of the three routes of administration for DNA prime vaccinations and among the vaccinees who receive each of the three routes of administration for rAd5 prime vaccinations. An order of magnitude difference between the IM route of administration and an alternate route of administration (i.e, SC or ID) at 4 weeks after completing the prime vaccination(s) (i.e., Week 12 for DNA prime schedules and Week 4 for rAd5 prime schedules) will be used for the exploratory evaluation of the effect of route of administration on immune response. Likewise at least an order of magnitude difference after the rAd5 booster vaccination will be used for the exploratory evaluation of the effect of priming route of administration on booster immune response.

**Table 6-3 Power to detect a significant difference in magnitude of ELISpot SFU between two groups of size 10 based on 2-sided alpha=0.05, equal variance t-test**

| | Difference in log(SFU) with corresponding “fold change” in parentheses | | | | | |
|---|---|---|---|---|---|---|
| SD in log(SFU) | | .3 (2) | .4 (2.5) | .5 (3) | .6 (4) | .7 (5) |
| | .3 | 56 | 80 | 94 | 98 | 99 |
| | .4 | 35 | 56 | 75 | 88 | 95 |
| | .5 | 24 | 39 | 56 | 71 | 84 |

Although the study sample size was chosen to provide the first Phase I safety evaluation of the different routes of administration, past experience with ELISpot in small Phase I studies suggests that there would be adequate power to detect a difference of .55 or larger in the magnitude of the $\log_{10}$ transformed count of spot-forming units from the ELISpot measure if the standard deviation

is approximately 0.4. A set of estimates of the standard deviation of log(SFU) within groups and at specific timepoints from some of the available data from VRC 007 yielded estimates of standard deviations between 0.3 and 0.5; therefore the power to detect a difference of a specified size for these standard deviations is shown in table 6-3. A three-fold increase would correspond to a difference on the log scale of 0.48; while the study was not designed based on the power to make these comparisons, there is a good chance that there will be adequate power to detect a three-fold difference based on our current best estimates of the standard deviation. While the magnitude of the response to ELISpot is of interest, so is the proportion of people who are judged as responders at each time point.

Table 6-4 gives the power to detect a difference for various proportions in two groups of size 30; the shaded cells show the combination of rates that give at least 80% power. This table can be used to compare the immune response rates in the strata with and without preexisting positive Ad5Ab titer (≥1:12). For example, if the true rate of immune response in one of the groups is .3, then the other group would have to have a true rate of at least .7 in order to have at least 80% power to detect a difference between the two groups. Table 6-5 shows similar calculations for comparing two groups of size 10.

**Table 6-4: Power to detect difference in two groups of size 30 based on underlying proportions**

| | Group 1 true proportion | | | | | | |
|---|---|---|---|---|---|---|---|
| Group 2 true proportion | | .3 | .4 | .5 | .6 | .7 | .8 |
| | .1 | 37 | 70 | 91 | 98 | >99 | >99 |
| | .2 | 8 | 29 | 59 | 85 | 97 | >99 |
| | .3 | | 7 | 25 | 56 | 83 | 97 |
| | .4 | 7 | | 7 | 25 | 56 | 85 |
| | .5 | 25 | 7 | | 7 | 25 | 59 |
| | .6 | 56 | 25 | 7 | | 7 | 29 |
| | .7 | 83 | 56 | 25 | 7 | | 8 |
| | .8 | 97 | 85 | 59 | 29 | 8 | |
| | .9 | >99 | 98 | 91 | 70 | 37 | 9 |

**Table 6-5: Power to detect difference in two groups of size 10 based on underlying proportions**

| | Group 1 true proportion | | | | | | | |
|---|---|---|---|---|---|---|---|---|
| Group 2 true proportion | | .3 | .4 | .5 | .6 | .7 | .8 | .9 |
| | .1 | 5 | 15 | 29 | 47 | 66 | 84 | 95 |
| | .2 | 2 | 5 | 13 | 25 | 42 | 63 | 84 |
| | .3 | | 2 | 5 | 12 | 24 | 44 | 66 |
| | .4 | 2 | | 2 | 5 | 12 | 25 | 47 |
| | .5 | 5 | 2 | | 2 | 5 | 13 | 29 |
| | .6 | 12 | 5 | 2 | | 2 | 5 | 15 |
| | .7 | 24 | 12 | 5 | 2 | | 2 | 5 |

## 6.5 STATISTICAL ANALYSIS

Since enrollment is concurrent with receiving the first study vaccination, all participants will have received at least one vaccination and therefore will provide some safety data.

All statistical analyses will be performed using SAS and S-Plus statistical software.

No formal multiple comparison adjustments will be employed for safety endpoints or secondary endpoints.

### 6.5.1 Analysis Variables

The analysis variables consist of baseline variables, safety variables, immunogenicity and social impact variables for primary and secondary objective analyses.

### 6.5.2 Baseline Demographics

Baseline characteristics including demographics and laboratory measurements will be summarized using descriptive statistics.

### 6.5.3 Safety Analysis

**Reactogenicities**

The number and percentage of participants experiencing each type of reactogenicity sign or symptom will be tabulated by severity. For a given sign or symptom, each participant's reactogenicity will be counted once under the maximum severity for all assessments. Reactogenicities for each priming route of administration will be tabulated separately. Reactogenicities for the booster rAd5 vaccination will be tabulated separately for the low titer and high titer subjects.

**Adverse Experiences**

Adverse experiences (AEs) are coded into MedDRA preferred terms. The number and percentages of participants experiencing each specific adverse event will be tabulated by severity and relationship to treatment. For the calculations in these tables, each participant's adverse experience will be counted once under the maximum severity or strongest recorded causal relationship to treatment.

Adverse experiences following priming vaccination with VRC-HIVDNA016-00-VP will be summarized separately from AEs following priming vaccination with VRC-HIVDNA014-00-VP. Adverse experiences occurring after the VRC-HIVADV014-00-VP booster vaccination through 18 weeks after the booster vaccination will be summarized separately.
A complete listing of adverse experiences for each participant will provide details including severity, relationship to treatment type, onset, duration and outcome.

**Local laboratory values**

Boxplots of local laboratory values will be generated for baseline values and for values measured during the course of the study. Each boxplot will show the 1st quartile, the median, and the 3rd quartile. Outliers, or values outside the boxplot, will also be plotted. If appropriate, horizontal lines representing boundaries for abnormal values will be plotted.

### 6.5.4 Immunogenicity Analysis

The statistical analysis for immunogenicity will employ the intent-to-treat principle, i.e., all data from enrolled participants will be used. The only exception will be to exclude data from HIV-infected participants at or post infection. If the HIV positivity status of an infected participant is unknown at the time that the first sample for immunogenicity assessments is drawn, then all data from that participant will be excluded from the analysis.

If assay data are qualitative (i.e., positive or negative) then analyses will be performed by tabulating the frequency of positive response for each assay at each time point that an assessment is performed. Binomial response rates will be presented with their corresponding exact 95% confidence interval estimates. Response rates by strata, injection method and Ad boost will be summarized in contingency tables and compared using Fisher's exact test. Missing responses will be assumed to be missing at random, i.e., conditional on the observed data the missingness is independent of the unobserved responses. Graphical descriptions of the longitudinal immune responses will also be given.

Some immunologic assays have underlying continuous or count-type readout that is often dichotomized into responder/nonresponder categories. For these assays, graphical and tabular summaries of the underlying distributions will be made. These summaries may be performed on transformed data (e.g., log transformation) to better satisfy assumptions of symmetry and homoscedasticity.

### 6.5.5 Social Impact Analysis

Social impacts will be tabulated by type of event and impact on quality of life. The number and percentage of participants experiencing each type of social impact will also be tabulated by impact on quality of life. For this calculation multiple events of the same type for a participant will be counted once under the maximum impact for all post-vaccination visits.

In addition, a listing will be generated of all participants who experienced a major disturbance of their quality of life due to study participation. The listing includes all social impacts experienced by these participants, descriptions of each impact, impact on quality of life and whether or not there was a resolution.

### 6.5.6 Interim Analyses

Interim analyses of immunogenicity for each Group may be performed after all ICS assays up to and including 4 weeks after completion of the priming vaccination have been completed on all participants in a Group and again 4 weeks after completion of the booster vaccinations have been completed on all participants in a Group. The purpose of the reports is to provide basic immunogenicity data to inform those who are making future clinical trial development-related decisions in a timely manner. The route of administration is not blinded. The results of this interim immunogenicity analysis will not influence the conduct of the VRC 011 trial in terms of early termination or completion of later safety or immunogenicity endpoint assessments.

# 7. PHARMACY PROCEDURES

## 7.1 STUDY AGENTS FOR PRIME-BOOST REGIMEN

### 7.1.1 DNA 6-Plasmid Vaccine, VRC-HIVDNA016-00-VP

The investigational DNA plasmid vaccine, VRC-HIVDNA016-00-VP, is produced under current Good Manufacturing Practices (cGMP) conditions by Vical Incorporated (San Diego, CA). It is composed of six closed circular plasmid DNA macromolecules. Plasmids VRC-4401, VRC-4409 and VRC-4404 are designed to express clade B HIV-1 Gag, Pol, or Nef, respectively. VRC-5736, VRC-5737, and VRC-5738 are designed to express HIV-1 Env glycoprotein from clade A, clade B, and clade C, respectively. The DNA plasmids have been modified to reduce toxicity.

The vaccine is supplied as a 2 mL glass vial containing a clear colorless isotonic sterile solution. Each vial contains 20% (mg) over the amount to be injected of each plasmid as shown in the table below. Each vial also contains GMP grade phosphate buffered saline (PBS). Each vaccine vial contains 1.2 mL as shown in the table below:

| Dose/mL | VRC-4401 | VRC-4409 | VRC-4404 | VRC-5736 | VRC-5737 | VRC-5738 | VRC-HIVDNA016-00-VP (mixture in study vials) |
|---|---|---|---|---|---|---|---|
| 4 mg/mL | 0.8 mg | 0.8 mg | 0.8 mg | 0.8 mg | 0.8 mg | 0.8 mg | 4.8 mg (1.2 mL) |

The vaccine will be shipped to the study pharmacist on dry ice and stored at -20° C or below until use. Vials of vaccine will be removed from the freezer and allowed to equilibrate to room temperature prior to preparing the injection.

One 1 mL injection of the 4 mg/mL preparation will be administered for each 4 mg dose.

Vials may be stored for the duration of the study (not to exceed 2 years) at -20°C or below. Vials should not be refrozen after thawing. VRC-HIVDNA016-00-VP will be tested for stability according to ICH (International Conference on Harmonization) Guidelines. Similar plasmids have been shown to be stable for a minimum of 24 months when stored at -30°C ± 10°C and for up to 24 hours when stored at room temperature. Vials are intended for single use only.

### 7.1.2 Adenoviral Vector Vaccine, VRC-HIVADV014-00-VP

The recombinant adenoviral vector product VRC-HIVADV014-00-VP (rAd5) is manufactured by GenVec, Inc (Gaithersburg, MD). It is produced under cGMP conditions by a contractor, Molecular Medicine (San Diego, CA). The rAd5 vaccine contains four recombinant serotype 5 adenoviral vectors. These vectors contain gene sequences that encode for clade B HIV-1 Gag and Pol as well as clade A, clade B, and clade C Env protein. *In vivo* expression by these vectors produces immunogens that induce an immune response against HIV. The envelope genes were chosen as representative primary isolates from each of the three clades.

Single use vials will be sent unblinded to the NIH Clinical Center pharmacy. The vial label notes a storage temperature of -10° C to -25° C. The product may be stored in a freezer that has temperatures as low as -30°C. However, if deviations in storage temperature below -30°C or above -10° C occur, the site pharmacist must report the storage temperature deviation promptly

to the IND sponsor. The product is shipped on dry ice, during which the product temperature is maintained at ≤ -60°C. Prior to shipping, the vials are sealed in Mylar bags to prevent $CO_2$ from inactivating the adenoviral vector product. The product vials should not be kept on dry ice without this protection. Once the VRC-HIVADV014-00-VP product is received at the clinical site it should be removed promptly from the dry ice package and the Mylar bag and stored as noted above.

The investigational vaccine vials will be provided at two different concentrations: 1 x $10^{10}$ PU/mL or 1 x $10^{11}$ PU/mL. Each vaccine vial will contain 1.2 mL/vial. The 1 x $10^{11}$ PU vials are used only for the ID administration of a 0.1 mL (i.e., 100 µL) volume of the rAd5 vaccine as described below in Section 7.2.2. Vials may be stored for the duration of the study (not to exceed two years). Vials of vaccine will be removed from the freezer and allowed to equilibrate to room temperature prior to preparing the injection. Vials should not be refrozen after thawing.

The lot release form notes the number of particle units (PU) in the final product. The clinical protocols specify the dose in particle units (PU). Particle units are the number of viral particles, active or not, found in the product as determined by spectrophotometry. Particle units, rather than plaque forming units (pfu) or fluorescent forming units (ffu), are used to determine dose because of the potential toxicity and host immune response to the viral particle, regardless of its ability to infect the target cells. It is also a more accurate measure than either pfu or ffu, which are highly dependent on methodology, and thus, more variable. Furthermore, the FDA and the Recombinant DNA Advisory Committee have recommended the use of "PU".

## 7.2 PREPARATION OF STUDY AGENT FOR ADMINISTRATION

Each study injection must be administered within 4 hours after removing the vaccine vial from the freezer.

### 7.2.1 Preparation of VRC-HIVDNA016-00-VP for Administration by Needle and Syringe

Intramuscular (IM) Injections: When the randomization plan includes IM injections of the DNA vaccine (Groups 1A and 1B), an individual syringe with 4 mg in a 1 mL volume will be prepared by the pharmacy and labeled with the subject identifier for transport to the clinic.

Subcutaneous (SC) Injections: When the randomization plan includes SC injections of the DNA vaccine (Groups 1C and 1D), an individual syringe with 4 mg in a 1 mL volume will be prepared by the pharmacy and labeled with the subject identifier for transport to the clinic.

Intradermal (ID) Injections: When the randomization plan includes ID injections of the DNA vaccine (Groups 1E and 1F), an individual syringe with 400 µg in a 100 µL volume will be prepared by the pharmacy and labeled with the subject identifier for transport to the clinic.

### 7.2.2 Preparation of VRC-HIVADV014-00-VP for Administration by Needle and Syringe

Intramuscular (IM) Injections: All randomization plans includes IM injections of the booster rAd5 vaccination and Groups 2A and 2B include a rAd5 prime vaccination by IM injection. To prepare a rAd5 $10^{10}$ PU IM injection, the pharmacy will prepare an individual syringe with 1 mL

volume from a 1 x $10^{10}$ PU/mL vial and label it with the subject identifier for transport to the clinic.

Subcutaneous (SC) Injections: The schedule for Groups 2C and 2D includes a SC injection of the rAd5 vaccine as the prime vaccination. To prepare a rAd5 $10^{10}$ PU SC injection, the pharmacy will prepare an individual syringe with 1 mL volume from a 1 x $10^{10}$ PU/mL vial and label it with the subject identifier for transport to the clinic.

Intradermal (ID) Injections: The schedule for Groups 2E and 2F includes an ID injection of the rAd5 vaccine as the prime vaccination. To prepare a rAd5 $10^{10}$ PU ID injection, the pharmacy will prepare an individual syringe with 0.1 mL volume from a 1 x $10^{11}$ PU/mL vial and label it with the subject identifier for transport to the clinic.

## 7.3 STUDY AGENT LABELING

Vials will be individually labeled with the name of the material, dose, pH, volume, lot number, concentration, storage instructions, Investigational Use Statement ("Caution: New Drug – Limited by Federal Law to Investigational Use"), and manufacturer information. If necessary, additional lots of vaccine will be produced.

## 7.4 PROCEDURES TO PRESERVE BLINDING

There are 6 vaccination schedules and each subject has an equal chance of being randomized to each of the 6 schedules. The subjects, the clinical staff, and the Principal Investigator will be blinded to treatment allocation until the subject enrollment is completed. After an enrollment is completed the assigned schedule becomes known to the subject and clinicians immediately after enrollment. The electronic enrollment database is programmed in advance with the randomized treatment assignment for each study ID number; this prints out of the database with the subject's enrollment confirmation. In addition, the pharmacist with primary responsibility for vaccine dispensing receives the randomization code from the protocol statistician in advance of opening the study. Each time a vaccine order is sent to the pharmacy by a study clinician, the pharmacist checks to ensure that the vaccine order matches the schedule assignment for that subject's study ID

The study pharmacist will be responsible for preparing the syringe with each vaccine dose indicated by the subject's randomization assignment and labeling it with the subject identification. The pharmacist will not be the same individual who is responsible for clinical follow-up.

## 7.5 STUDY AGENT ACCOUNTABILITY

### 7.5.1 Documentation

The study pharmacist will be responsible for maintaining an accurate record of the codes, inventory, and an accountability record of vaccine supplies for this study. Electronic documentation as well as paper copies will be used.

### 7.5.2 Disposition

The empty vials and the unused portion of a vial will be discarded in a biohazard containment

bag and incinerated or autoclaved. Any unopened vials that remain at the end of the study will be returned to the production facility or discarded at the discretion of the sponsor in accordance with policies that apply to investigational agents. Partially used vials will not be administered to other subjects or used for *in vitro* experimental studies. They will be disposed of in accordance with institutional or pharmacy policy.

# 8. HUMAN SUBJECT PROTECTIONS AND ETHICAL OBLIGATIONS

This research study will be conducted in compliance with the protocol, Good Clinical Practices (GCP), and all applicable regulatory requirements.

## 8.1 INFORMED CONSENT

The study informed consent is provided in Appendix I. It describes the investigational product to be used and all aspects involved in protocol participation.

Before a subject's participation in the study, it is the investigator's responsibility to obtain written informed consent from the subject, after adequate explanation of the aims, methods, anticipated benefits, and potential hazards of the study and before any protocol-specific procedures or study medications are administered.

The acquisition of informed consent will be documented in the subject's medical records, as required by 21 CFR 312.62. The informed consent form will be signed and personally dated by the subject and the person who conducted the informed consent discussion. The original signed informed consent form will be retained in the medical chart and a copy will be provided to the subject.

## 8.2 RISKS AND BENEFITS

### 8.2.1 Risks

VRC-HIVDNA016-00-VP: The risks noted for the DNA vaccine, VRC-HIVDNA016-00-VP, are based on risks of injections, risks of vaccines in general and interim results of a previous Phase 1 study with this vaccine, as well as other investigational HIV-1 DNA vaccines.

Potential side effects resulting from intramuscular injection include stinging, arm discomfort, or redness of the skin at vaccine injection sites. Intradermal and subcutaneous injections have the same risks, as well as pruritis. Subjects may exhibit general signs and symptoms associated with administration of a vaccine injection, including fever, chills, rash, aches and pains, nausea, headache, dizziness and fatigue. These side effects will be monitored, but are generally short term and do not require treatment. Study subjects may self administer medications such as acetaminophen, nonsteroidal anti-inflammatory drugs (NSAIDs), or antihistamines as required. Glucocorticoids will not be used in these study subjects; if such medication is required the study subject will receive no further immunizations, but continue to be monitored in follow-up visits.

Potential risks of DNA vaccines include: muscle damage, antibodies to DNA, insertion of the vaccine DNA into genomic DNA (a potential cancer risk), or insertion of the vaccine DNA into a bacteria or virus. Although these risks are possible, they have not been observed to date in laboratory, animal or human testing of DNA plasmid vaccines.

The most extensive human experience with a multiclade DNA vaccine study is with VRC-HIVDNA009-00-VP. In study VRC 004 (03-I-0022) 40 of the 50 subjects enrolled received

vaccine and 10 received placebo. This randomized study was unblinded in September 2004. Both placebo and vaccine recipients were noted to have occasional asymptomatic and self-limited changes in glucose, bilirubin, liver enzymes and urine protein. In the vaccine groups, there were three reportable adverse events possibly related to vaccine. These were a grade 3 asymptomatic neutropenia seen on test results from 27 days after 3rd vaccination (4 mg group) that was normal on repeat testing 5 days later, a grade 3 urticaria with onset 4 days after 3rd vaccination (4 mg group) and a grade 2 maculopapular rash with onset 27 days after 2nd vaccination (8 mg group). All resolved without sequelae. Other factors in the occurrence of the urticaria include concomitant bladder infection, yeast infection and multiple antibiotics. The rash resulted in discontinuation from the vaccination schedule after the 2nd injection and it was clinically consistent with either a drug eruption or a viral exanthem. Informed consents for protocols using similar DNA vaccines should note the potential risk of neutropenia, rash and hives. Extramural randomized, blinded studies with this vaccine are ongoing.

The multiclade DNA vaccine that will be used in this study is VRC-HIVDNA016-00-VP, which has been administered to 15 subjects in the open-label study VRC 007 (04-I-0254). No subject reported fever following vaccination. Reactogenicity was none to mild except that two subjects reported moderate injection site pain and one subject reported moderate nausea and malaise. There has been one grade 3 adverse event (chronic urticaria possibly related to vaccination) requiring expedited reporting to the IND sponsor. To date, there have been two moderate (grade 2) adverse events possibly attributed to vaccine. These were intermittent dizziness of 2 days duration beginning 13 days after the second vaccination in one subject (this subject received the third vaccination without recurrence of symptoms) and asymptomatic hypoglycemia in another subject, first noted at the follow-up visit that was 14 days after the third vaccination. Four of the 44 injections were associated with mild skin lesions (0.5-1.0 cm diameter) at the vaccination site. A small scab formed within a week after immunization and came off after a few days. The skin healed without treatment within a few weeks. One skin biopsy was obtained on day 6 post vaccination. It showed subcutaneous and dermal perivascular lymphocytic inflammation. There were rare eosinophils and rare giant cells noted, and the infiltrate was composed entirely of CD3 positive cells. It included both $CD4^+$ and $CD8^+$. The process appears to be primarily a subcutaneous inflammatory response to vaccination with cutaneous manifestations. There have been no serious adverse events to date.

VRC-HIVADV014-00-VP: The adenoviral vector vaccine, VRC-HIVADV014-00-VP has previously been administered at the NIH Clinical Center in Phase I clinical trials at dosages up to $10^{11}$ PU per injection. The first extramural study using this vaccine opened in November 2004. At the $10^{10}$ PU dose none of the subjects in the first Phase I study had fever and the other reactogenicity was mild or none. At the next higher dose ($10^{11}$ PU) four subjects had a flu-like set of symptoms with fever, headache, muscle aches, malaise and chills starting 12-16 hours after vaccination and lasting a few hours. Some of these symptoms were moderate in severity. A few subjects have had nausea. Some subjects have had injection site pain or discomfort in the first few days after a vaccination. These symptoms improved after treatment with over-the-counter medicine.

One subject with a history of intermittent low neutrophil count, had a neutrophil count that was moderately below normal shortly after the study vaccination. This returned to normal without any symptoms of illness. A different subject was noted to have persistent grade 1 ALT starting 25 days after study injection that lasted for about 5 months. An evaluation diagnosed fatty liver

(steatohepatitis). There were no clinical symptoms. The condition may have existed prior to study enrollment and the subject's alcohol use and recent weight gain may be contributing factors. One subject with a history of a single seizure three years prior to study enrollment, experienced a seizure 64 days after study injection. This subject now has a diagnosis of epilepsy and is on anticonvulsant therapy. Following review of the subject's medical records and given the timing of the seizure, the seizure was assessed as unrelated to study vaccine. Other subjects have had mild temporary changes in blood or urine tests. It is unknown whether the lab test changes, diarrhea, fatty liver, or seizure were due to vaccine or to other factors or to a combination of the vaccine with other factors.

The effect of the study vaccines on a fetus or nursing baby is unknown, so female subjects of child bearing potential will be required to agree to use birth control for sexual intercourse beginning 21 days prior to enrollment and continuing through Week 42. Women who are pregnant or nursing will be excluded from the study.

Either vaccine may cause a positive HIV antibody test using the standard screening test. A positive or indeterminate test may have a negative employment and social impact. Western blot analysis and HIV PCR or other testing will be done to either exclude or confirm HIV infection. ELISA, Western Blot, and PCR results will be discussed with the study subject as they become available.

Blood drawing may cause pain, bruising; may infrequently cause a feeling of lightheadedness or fainting, and rarely, may cause infection at the site where the blood is taken.

Subjects may believe that this vaccine provides protection, and therefore practice riskier behavior. They will receive extensive counseling throughout the study to address this potential problem.

#### 8.2.2 Benefits

It is unknown if any benefit will result from study participation. Others may benefit from knowledge gained in this study that may aid in the development of an HIV vaccine.

### 8.3 INSTITUTIONAL REVIEW BOARD

A copy of the protocol, proposed informed consent form, other written subject information, and any proposed advertising material will be submitted to the IRB for written approval.

The investigator must submit and, where necessary, obtain approval from the IRB for all subsequent protocol amendments and changes to the informed consent document. The investigator will notify the IRB of deviations from the protocol and serious adverse events.

The investigator will be responsible for obtaining IRB approval of the annual Continuing Review throughout the duration of the study.

### 8.4 PROTOCOL REGISTRATION

The Division of AIDS, NIAID is the IND sponsor for this protocol. Protocol registration must occur before subjects are enrolled in this study. The Institutional Review Board (IRB) must approve the protocol and consent form. The protocol must be submitted to the Institutional Biosafety Committee (IBC). Approval letters from both the IRB and IBC must be submitted to the Division of AIDS Regulatory Compliance Center (RCC) Protocol Registration Office (see

Appendix II) with the initial protocol registration. Subsequent protocol amendments must also be registered with and approved by the RCC Protocol Registration Office.

### 8.5 SUBJECT CONFIDENTIALITY

The investigator must ensure that the subject's anonymity is maintained. Individual identifying information will not be included in any reports; subjects will be identified only by coded numbers. All records will be kept confidential to the extent provided by federal, state and local law. Medical records are made available for review when required by the Food and Drug Administration or other authorized users, such as the vaccine manufacturer, only under the guidelines set by the Federal Privacy Act. Direct access includes examining, analyzing, verifying, and reproducing any records and reports that are important to the evaluation of the study. The investigator is obligated to inform the subjects that the above named representatives will review their study-related records without violating the confidentiality of the subjects.

### 8.6 PLAN FOR USE AND STORAGE OF BIOLOGICAL SAMPLES

The June 12, 2006 memorandum "Research Use of Stored Human Samples, Specimens or Data" requires that all NIH IRB-approved protocols in which intramural research program researchers intend to collect and store human specimens or data must include a written description of the intended use of the samples; how they will be stored; how they will be tracked; what will happen to them at the completion of the protocol, and what circumstances would prompt the PI to report to the IRB loss or destruction of samples. We will apply the specified provisions to the stored samples from this protocol as follows:

.

**Intended use of the samples/specimens/data**:

Samples, specimens and data collected under this protocol may be used to conduct protocol-related safety and immunogenicity evaluations, exploratory laboratory evaluations related to the type of infection the vaccine was designed to prevent, exploratory laboratory evaluations related to vaccine research in general and for research assay validation. Genetic testing may be performed in accordance with the genetic testing information that was included in the study informed consent.

**How stored samples, specimens and data from sample use will be stored**

All of the stored study research samples are labeled by a code (such as a number) that only the VRC Clinic can link to the subject. Samples are stored at the NIAID Vaccine Immune T-Cell and Antibody Laboratory (NVITAL), Gaithersburg, MD or VRC Laboratories in Building 40, which are both secure facilities with limited access. Data will be kept in password-protected computers. Only investigators or their designees will have access to the samples and data.

**How samples/specimens/data will be tracked**

Samples will be tracked in the Laboratory Information Management System (LIMS) database and uses of these samples are documented in the LIMS.

**What will happen to the samples/specimens/data at the completion of the protocol**

In the future, other investigators (both at NIH and outside) may wish to study these samples and/or data. IRB approval must be sought prior to any sharing of samples with investigators and any clinical information shared about those samples would similarly require prior IRB approval.

The research use of stored, unlinked or unidentified samples may be exempt from the need for prospective IRB review and approval. Exemption requests will be submitted in writing to the NIH Office of Human Subjects Research, which is authorized to determine whether a research activity is exempt.

At the time of protocol termination, samples will remain in the NVITAL facility or VRC laboratories or, after IRB approval, transferred to another repository. Data will be archived by the VRC in compliance with requirements for retention of research records, or after IRB and study sponsor approval, it may be either destroyed or transferred to another repository.

**Circumstances that would prompt the PI to report loss or destruction of samples/specimens/data to the IRB**

The NIH Intramural Protocol Violation definition related to loss of or destruction of samples will be followed in reporting to the IRB. Any loss or unanticipated destruction of samples (for example, due to freezer malfunction) or data (for example, misplacing a printout of data with identifiers) that would affect the scientific integrity of the study will be reported to the IRB. The PI will also notify the IRB if the decision is made to destroy the remaining samples.

## 8.7 SUBJECT IDENTIFICATION AND ENROLLMENT OF STUDY PARTICIPANTS

All study activities will be carried out at the Clinical Center at the National Institutes of Health. Study subjects will be recruited through on-site and off-site advertising done for the screening protocol, VRC 000 (02-I-0127). Effort will be made to include women and minorities in proportions similar to that of the community from which they are recruited. Because this Phase I study is designed to establish safety of the vaccine in healthy adults, enrollment will be limited to persons at least 18 years of age, and no older than 50 years of age.

### 8.7.1 Participation of Children

Children are not eligible to participate in this clinical trial because it does not meet the guidelines for inclusion of children in research. These guidelines (45 CFR 46, Subpart D, 401-409), state the Department of Health and Human Services protections for children who participate in research. Generally, healthy children can be studied when the research is considered as "not greater than minimal risk." Children can be involved in research with greater than minimal risk only when it presents the prospect of direct benefit to the individual child or is likely to yield generalizable knowledge about the child's disorder or condition.

## 8.8 COMPENSATION

Subjects will be compensated for time and inconvenience in accordance with the standards for compensation of the Clinical Research Volunteer Program. The compensation per visit will be $200 for visits that include injections and blood drawing, $175 for visits that include blood drawing but no injection, and $75 for visits for injection site inspection (and photo if needed). The approximate total compensation for the subject will be between $2425 (for Group 1) and $1700 (for Group 2), based on the projected 15 and 10 clinic visits, respectively, that include the schedule-specific number of study injections through Week 42. Subjects who consent to a skin biopsy will be compensated $100 per skin biopsy. Those who return at Week 94 for a long-term follow-up blood draw will be compensated $175 for that visit.

### 8.9 SAFETY MONITORING

Close cooperation between the designated members of the Protocol Team will occur to evaluate and respond to individual adverse events in a timely manner. Designated team members (Principal Investigator, Medical Officer, Protocol Specialist, Study Coordinator and other study clinicians) will review the summary study safety data reports on a weekly basis through 4 weeks after the last subject receives the last study injection in order to be certain that the vaccine has an acceptable safety profile and will continue to monitor the study safety data reports on a monthly basis through completion of the last Week 42 visit. The DAIDS Medical Officer will provide an independent review of adverse events that have a bearing on study stopping (see Section 4.7).

## 9. ADMINISTRATIVE AND LEGAL OBLIGATIONS

### 9.1 PROTOCOL AMENDMENTS AND STUDY TERMINATION

Protocol Amendments must be made only with the prior approval of the National Institute of Allergy and Infectious Diseases' Division of AIDS and Vaccine Research Center. Agreement from the investigator must be obtained for all protocol amendments and amendments to the informed consent document. All study amendments will be submitted to the IRB for approval.

The Division of AIDS, National Institute of Allergy and Infectious Diseases, the Vaccine Research Center, the Principal Investigator and the Food and Drug Administration reserve the right to terminate the study. The investigator will notify the IRB in writing of the study's completion or early termination.

### 9.2 STUDY DOCUMENTATION AND STORAGE

The investigator will maintain a list of appropriately qualified persons to whom trial duties have been delegated.

Source documents are original documents, data, and records from which the subject's data are obtained. These include but are not limited to hospital records, clinical and office charts, laboratory and pharmacy records, diaries, microfiches, radiographs, and correspondence.

The investigator and staff are responsible for maintaining a comprehensive and centralized filing system of all study-related (essential) documentation, suitable for inspection at any time by representatives from the National Institute of Allergy and Infectious Diseases' Division of AIDS and Vaccine Research Center, IRB, FDA, and/or applicable regulatory authorities. Elements include:

- Subject files containing completed informed consent forms, and supporting copies of source documentation (if kept)
- Study files containing the protocol with all amendments, Investigator Brochures, copies of all correspondence with the IRB and the National Institute of Allergy and Infectious Diseases' Division of AIDS and Vaccine Research Center

In addition, all original source documentation must be maintained and be readily available.

All essential documentation should be retained by the institution for the same period of time required for medical records retention. The FDA requires study records to be retained for up to two years after marketing approval or refusal (21 CFR 312.62). No study document should be destroyed without prior written agreement between the National Institute of Allergy and

Infectious Diseases' Division of AIDS and Vaccine Research Center and the investigator. Should the investigator wish to assign the study records to another party or move them to another location, they must notify the National Institute of Allergy and Infectious Diseases' Division of AIDS and Vaccine Research Center in writing of the new responsible person and/or the new location.

## 9.3 STUDY MONITORING, DATA COLLECTION AND DATA MONITORING

### 9.3.1 Study Monitoring

The National Institute of Allergy and Infectious Diseases' Division of AIDS and Vaccine Research Center regulatory authority inspectors or their authorized representatives are responsible for contacting and visiting the investigator for the purpose of inspecting the facilities and, upon request, inspecting the various records of the trial, provided that subject confidentiality is respected.

Site visits by study monitors will be made in accordance with the IND Sponsor (DAIDS) policy to monitor the following: study operations, the quality of data collected in the research records, the accuracy and timeliness of data entered in the database, and to determine that all process and regulatory requirements are met.

Site investigators will allow the study monitors, the NIAID IRB, and the FDA to inspect study documents (e.g., consent forms, drug distribution forms, case report forms) and pertinent hospital or clinic records for confirmation of the study data.

### 9.3.2 Data Collection

Clinical research data will be collected in a secure electronic data management system through a contract research organization, EMMES (Rockville, MD). Extracted data without patient identifiers will be sent to the Protocol Statistician for statistical analysis.

## 9.4 LANGUAGE

All written information and other material to be used by subjects and investigative staff must use vocabulary and language that are clearly understood.

## 9.5 POLICY REGARDING RESEARCH-RELATED INJURIES

The Clinical Center will provide short-term medical care for any injury resulting from participation in this research. In general, the National Institutes of Health, the Clinical Center, or the Federal Government will provide no long-term medical care or financial compensation for research-related injuries.

## 10. REFERENCES

1. WHO, *Treating 3 Million by 2005: The WHO Strategy*. 2003, World Health Organization: Geneva, Switzerland. p. 1-53.

2. UNAIDS/WHO. *AIDS Epidemic Update - December 2004.* 2004 [cited; Available from: http://www.unaids.org/wad2004/report_pdf.html.

3. *Approaches to the development of broadly protective HIV vaccines: challenges posed by the genetic, biological and antigenic variability of HIV-1: Report from a meeting of the WHO-UNAIDS Vaccine Advisory Committee Geneva, 21-23 February 2000.* AIDS, 2001. **15**(6): p. W1-W25.

4. Nabel, G., W. Makgoba, and J. Esparza, *HIV-1 Diversity and Vaccine Development.* Science, 2002. **296**(5577): p. 2335.

5. Osmanov, S., et al., *Estimated global distribution and regional spread of HIV-1 genetic subtypes in the year 2000.* J Acquir Immune Defic Syndr, 2002. **29**(2): p. 184-90.

6. Graham, B., *Safety and immunogenicity of a multiclade HIV-1 recombinant adenovirus vacine boost in prior recipients of a multiclade HIV-1 DNA vaccine* in AIDS Vaccine 2005 Conference Abstracts. Montreal, Quebec, Canada.

7. Sugaya, M., et al., *HIV-infected Langerhans cells preferentially transmit virus to proliferating autologous CD4+ memory T cells located within Langerhans cell-T cell clusters.* J Immunol, 2004. **172**(4): p. 2219-24.

8. Banchereau, J. and R.M. Steinman, *Dendritic cells and the control of immunity.* Nature, 1998. **392**(6673): p. 245-52.

9. Peachman, K.K., M. Rao, and C.R. Alving, *Immunization with DNA through the skin.* Methods, 2003. **31**(3): p. 232-42.

10. Roozbeh, J., et al., *Low dose intradermal versus high dose intramuscular hepatitis B vaccination in patients on chronic hemodialysis.* Asaio J, 2005. **51**(3): p. 242-5.

11. Kenney, R.T., et al., *Dose sparing with intradermal injection of influenza vaccine.* N Engl J Med, 2004. **351**(22): p. 2295-301.

12. Belshe, R.B., et al., *Serum antibody responses after intradermal vaccination against influenza.* N Engl J Med, 2004. **351**(22): p. 2286-94.

13. Ristola, M.A., et al., *Antibody responses to intradermal recombinant hepatitis B immunization among HIV-positive subjects.* Vaccine, 2004. **23**(2): p. 205-9.

14. Roy, M.J., et al., *Induction of antigen-specific CD8+ T cells, T helper cells, and protective levels of antibody in humans by particle-mediated administration of a hepatitis B virus DNA vaccine.* Vaccine, 2000. **19**(7-8): p. 764-78.

15. Epstein, J.E., et al., *Safety, tolerability, and lack of antibody responses after administration of a PfCSP DNA malaria vaccine via needle or needle-free jet injection, and comparison of intramuscular and combination intramuscular/intradermal routes.* Hum Gene Ther, 2002. **13**(13): p. 1551-60.

16. Mumper, R.J. and Z. Cui, *Genetic immunization by jet injection of targeted pDNA-coated nanoparticles.* Methods, 2003. **31**(3): p. 255-62.

17. Aguiar, J.C., et al., *Enhancement of the immune response in rabbits to a malaria DNA vaccine by immunization with a needle-free jet device.* Vaccine, 2001. **20**(1-2): p. 275-80.

18. Jackson, L.A., et al., *Safety and immunogenicity of varying dosages of trivalent inactivated influenza vaccine administered by needle-free jet injectors.* Vaccine, 2001. **19**(32): p. 4703-9.

19. Williams, J., et al., *Hepatitis A vaccine administration: comparison between jet-injector and needle injection.* Vaccine, 2000. **18**(18): p. 1939-43.

20. Mathei, C., P. Van Damme, and A. Meheus, *Hepatitis B vaccine administration: comparison between jet-gun and syringe and needle.* Vaccine, 1997. **15**(4): p. 402-4.

21. Fisch, A., et al., *Immunogenicity and safety of a new inactivated hepatitis A vaccine: a clinical trial with comparison of administration route.* Vaccine, 1996. **14**(12): p. 1132-6.

22. Manam, S., et al., *Plasmid DNA vaccines: tissue distribution and effects of DNA sequence, adjuvants and delivery method on integration into host DNA.* Intervirology, 2000. **43**(4-6): p. 273-81.

23. Wang, R., et al., *Induction of CD4(+) T cell-dependent CD8(+) type 1 responses in humans by a malaria DNA vaccine.* Proc Natl Acad Sci U S A, 2001. **98**(19): p. 10817-22.

24. Kennedy, J. *Interim report of a Phase 1 clinical trial evaluating the safety and immunogenicity of an HIV-1 DNA prime-boost vaccine (DP6-001) in healthy volunteers*. in AIDS Vaccine 2005 Conference Abstracts. Montreal, Quebec, Canada.

25. CytRx (2005) *DNA/Protein Vaccine Candidate DP6-001 Demonstrates Ability to Produce Neutralizing Antibody Responses to Multiple AIDS Virus Strains*. Press Release September 7, 2005, http://www.cytrx.com/prDetail.cfm?pr_id=213&showcspr=1.

26. Sadagopal, S., et al., *Signature for long-term vaccine-mediated control of a Simian and human immunodeficiency virus 89.6P challenge: stable low-breadth and low-frequency*

*T-cell response capable of coproducing gamma interferon and interleukin-2.* J Virol, 2005. **79**(6): p. 3243-53.

27. Harvey, B.G., et al., *Airway epithelial CFTR mRNA expression in cystic fibrosis patients after repetitive administration of a recombinant adenovirus.* J Clin Invest, 1999. **104**(9): p. 1245-55.

28. Bellon, G., et al., *Aerosol administration of a recombinant adenovirus expressing CFTR to cystic fibrosis patients: a phase I clinical trial.* Hum Gene Ther, 1997. **8**(1): p. 15-25.

29. Hay, J.G., et al., *Modification of nasal epithelial potential differences of individuals with cystic fibrosis consequent to local administration of a normal CFTR cDNA adenovirus gene transfer vector.* Hum Gene Ther, 1995. **6**(11): p. 1487-96.

30. Harvey, B.G., et al., *Cellular immune responses of healthy individuals to intradermal administration of an E1-E3- adenovirus gene transfer vector.* Hum Gene Ther, 1999. **10**(17): p. 2823-37.

31. Rosengart, T.K., et al., *Six-month assessment of a phase I trial of angiogenic gene therapy for the treatment of coronary artery disease using direct intramyocardial administration of an adenovirus vector expressing the VEGF121 cDNA.* Ann Surg, 1999. **230**(4): p. 466-70; discussion 470-2.

32. Rosengart, T.K., et al., *Angiogenesis gene therapy: phase I assessment of direct intramyocardial administration of an adenovirus vector expressing VEGF121 cDNA to individuals with clinically significant severe coronary artery disease.* Circulation, 1999. **100**(5): p. 468-74.

33. Sterman, D.H., et al., *Adenovirus-mediated herpes simplex virus thymidine kinase/ganciclovir gene therapy in patients with localized malignancy: results of a phase I clinical trial in malignant mesothelioma.* Hum Gene Ther, 1998. **9**(7): p. 1083-92.

34. Clayman, G.L., et al., *Adenovirus-mediated wild-type p53 gene transfer as a surgical adjuvant in advanced head and neck cancers.* Clin Cancer Res, 1999. **5**(7): p. 1715-22.

35. Swisher, S.G., et al., *Adenovirus-mediated p53 gene transfer in advanced non-small-cell lung cancer.* J Natl Cancer Inst, 1999. **91**(9): p. 763-71.

36. Gahery-Segard, H., et al., *Phase I trial of recombinant adenovirus gene transfer in lung cancer. Longitudinal study of the immune responses to transgene and viral products.* J Clin Invest, 1997. **100**(9): p. 2218-26.

37. Tursz, T., et al., *Phase I study of a recombinant adenovirus-mediated gene transfer in lung cancer patients.* J Natl Cancer Inst, 1996. **88**(24): p. 1857-63.

38. Crystal, R.G., et al., *Analysis of risk factors for local delivery of low- and intermediate-dose adenovirus gene transfer vectors to individuals with a spectrum of comorbid conditions.* Hum Gene Ther, 2002. **13**(1): p. 65-100.

39. Harvey, B.G., et al., *Safety of local delivery of low- and intermediate-dose adenovirus gene transfer vectors to individuals with a spectrum of morbid conditions.* Hum Gene Ther, 2002. **13**(1): p. 15-63.

40. *Assessment of adenoviral vector safety and toxicity: report of the National Institutes of Health Recombinant DNA Advisory Committee.* Hum Gene Ther, 2002. **13**(1): p. 3-13.

41. Isaacs, R., *Evaluating the efficacy of the Merck adenovirus serotype 5-based trivalent MRKAD5GAG/POL/NEF (paper #42)*, in *AIDS Vaccine 2005*. 2005: Montreal, Canada.

42. Isaacs, R., *Impact of pre-existing immunity on the immunogenicity of Ad5-based vaccines*, in *AIDS Vaccine 2004*. 2004: Lausanne, Switzerland.

43. Harvey, B.G., et al., *Variability of human systemic humoral immune responses to adenovirus gene transfer vectors administered to different organs.* J Virol, 1999. **73**(8): p. 6729-42.

44. Helms, T., et al., *Direct visualization of cytokine-producing recall antigen-specific CD4 memory T cells in healthy individuals and HIV patients.* J Immunol, 2000. **164**(7): p. 3723-32.

45. Betts, M.R., J.P. Casazza, and R.A. Koup, *Monitoring HIV-specific CD8+ T cell responses by intracellular cytokine production.* Immunol Lett, 2001. **79**(1-2): p. 117-25.

46. Malenbaum, S.E., D. Yang, and C. Cheng-Mayer, *Evidence for similar recognition of the conserved neutralization epitopes of human immunodeficiency virus type 1 envelope gp120 in humans and macaques.* J Virol, 2001. **75**(19): p. 9287-96.

47. Mascola, J.R., et al., *Recommendations for the design and use of standard virus panels to assess neutralizing antibody responses elicited by candidate human immunodeficiency virus type 1 vaccines.* J Virol, 2005. **79**(16): p. 10103-7.

48. Li, M., et al., *Human immunodeficiency virus type 1 env clones from acute and early subtype B infections for standardized assessments of vaccine-elicited neutralizing antibodies.* J Virol, 2005. **79**(16): p. 10108-25.

49. Sprangers, M.C., et al., *Quantifying adenovirus-neutralizing antibodies by luciferase transgene detection: addressing preexisting immunity to vaccine and gene therapy vectors.* J Clin Microbiol, 2003. **41**(11): p. 5046-52.

50. Peng, B. and M. Robert-Guroff, *Deletion of N-terminal myristoylation site of HIV Nef abrogates both MHC-1 and CD4 down-regulation.* Immunol Lett, 2001. **78**(3): p. 195-200.

51. Liang, X., et al., *Development of HIV-1 Nef vaccine components: immunogenicity study of Nef mutants lacking myristoylation and dileucine motif in mice.* Vaccine, 2002. **20**(27-28): p. 3413-21.

52. Chakrabarti, B.K., et al., *Modifications of human immunodeficiency virus envelope glycoprotein enhance immunogenicity for genetic immunization.* J Virol, 2002. **76**(11): p. 5357-5368.

53. Barouch, D.H., et al., *A human T-cell leukemia virus type 1 regulatory element enhances the immunogenicity of human immunodeficiency virus type 1 DNA vaccines in mice and nonhuman primates.* J Virol, 2005. **79**(14): p. 8828-34.

54. Seth, P., *Adenoviruses : basic biology to gene therapy*. Medical intelligence unit. 1999, Austin, TX: R.G. Landes Co.

55. Brough, D.E., et al., *A gene transfer vector-cell line system for complete functional complementation of adenovirus early regions E1 and E4.* J Virol, 1996. **70**(9): p. 6497-501.

56. Rasmussen, H., et al., *TNFerade Biologic: preclinical toxicology of a novel adenovector with a radiation-inducible promoter, carrying the human tumor necrosis factor alpha gene.* Cancer Gene Ther, 2002. **9**(11): p. 951-7.

DATE: January 15, 2008

RE: Letter of Amendment for VRC 011 (06-I-0149), Version 2.0 (March 6, 2007)

TO: Division of AIDS and NIAID IRB

FROM: Barney S. Graham, M.D., Ph.D., Principal Investigator

# Letter of Amendment #1

**This Letter of Amendment impacts the VRC 011 (06-I-0149), Version 2.0 (March 6, 2007) study and will be forwarded to the institutional review board (IRB) for their information and review. This must be approved by the IRB before implementation.**

The Division of AIDS applies the following conditions to a protocol Letter of Amendment:
The site IRB is responsible for determining whether the contents of this Letter of Amendment (LoA) require any changes to the study consent. This letter and any IRB correspondence with regard to this letter will be filed in the protocol regulatory file and other pertinent files. A Letter of Amendment is not required to be submitted to the IND Sponsor's protocol registration office unless the changes result in a change to the informed consent.

**Purpose of the Letter of Amendmen**t: At the time of this LoA, the protocol is fully enrolled and more than half of the subjects have completed the required study visits. Few study injections remain to be administered. The purpose of this LoA includes the following:

1. To amend the plan for completing injections of the VRC rAd5 vaccine booster injections in consideration of the recent results of the Step Study in which a different rAd5 vaccine was administered and a potential safety concern had been made public.
2. To amend the "List of Contacts" information in Appendix II of the current protocol.

**Preliminary Results of the Step Study (HVTN 502) with the Merck rAd5 vaccine**:
The preliminary results of the Step Study showed a trend in men with pre-existing immunity to adenovirus serotype 5 (Ad5) [as measured by positive antibody (Ab) titer prior to study enrollment] who then received the Merck rAd5 vaccine, suggesting an increased susceptibility to HIV infection if later exposed to HIV through the circumstances of their lives. The Step Study data do not indicate such a trend for subjects who are Ad5 Ab negative prior to vaccination with the Merck rAd5 vaccine.

**Considerations for the VRC rAd5 Vaccine**: The VRC rAd5 vaccine is different in several important respects from the Merck rAd5 vaccine. There are not similar data for the VRC rAd5 vaccine regarding susceptibility to HIV infection. However, a conservative approach to subject safety requires that the Merck rAd5 Step Study data be considered in the completion of the VRC 011 study injections. The VRC 011 study was paused on October 19, 2007 until data from the


40 CONVENT DRIVE, BETHESDA, MD 20892-3017
www.vrc.nih.gov

Step Study were made public and VRC and the IND Sponsor, DAIDS, had time to consider future study plans. VRC and DAIDS have concurred with the following plan:

1. All study participants will receive the attached information letter regarding the potential safety concern identified by the Step Study and the subjects who will be receiving additional study injections will also be re-consented.

2. VRC 011 Subjects with negative (<1:12) Ad5 Ab titer at enrollment who have not yet completed the rAd5 booster injection will be provided with the information letter and an updated consent. If willing to proceed, these subjects may receive the rAd5 booster injection. Since some subjects missed the protocol-preferred window for the booster injection (visit 06), the injection may be administered outside the protocol window, at the earliest convenience to the subject, as there is not a safety issue related to a longer boost interval. Follow-up visits will be adjusted as needed to the interval indicated by the protocol schedule (see Appendix III of the protocol).

3. VRC 011 Subjects with a positive (≥1:12) Ad5 Ab titer at enrollment who have not yet completed the rAd5 booster injection will be informed that the booster will not be administered and provided with the information letter.

4. VRC 011 subjects with both negative and positive Ad5 Ab titers at enrollment may complete any of the DNA injections remaining to be administered. These subjects will be given the information letter and the new consent form and asked for re-consent. Since some subjects missed the protocol-preferred window for the DNA prime injections (visits 03 or 04), the injection may be administered outside the protocol window, at the earliest convenience to the subject, as there is not a safety issue related to longer intervals between injections. Follow-up visits will be adjusted as needed to the interval indicated by the protocol schedule (see Appendix III of the protocol).

In this open label study, all subjects are aware of their negative or positive Ad5 Ab status and vaccination schedule assignment already. All will continue with HIV risk reduction counseling as has been ongoing throughout the study.

**<u>Study Informed Consent Update</u>**: Changes to the Study Consent are itemized below and the revised consent (Appendix I of the protocol) is included as an attachment to this LoA.

- The estimated number of people who have received the DNA vaccine was updated in the section called "DNA Vaccine Risks".
- The estimated number of people who have received the VRC rAd5 vaccine was updated to 800 in the section previously called "**Adenoviral Vector (rAd5) Vaccine Risks;"** this section now is called "**VRC Adenoviral Vector (rAd5) Vaccine Risks"**
- A new paragraph about the Step Study was added to the end of the section previously called "<u>Other Adenoviral Vectors</u>"; this section is now called "<u>Other Adenoviral Vector Vaccines</u>"
- A new sentence was added to the end of the section previously called "<u>Adenovirus antibodies;</u>" this section is now called "<u>Adenovirus Antibodies from Vaccine</u>"
- Bold and underline was added to an existing sentence in the section called "**Risks of Being in an HIV Vaccine Study**"

**<u>Contact Information Updates</u>**: Changes to the List of Contacts are itemized below and a revised Appendix II is included as an attachment to this LoA.

- Addition of Raymond Cruz, M.D. as a Subinvestigator
- Addition of Cynthia Starr Hendel, CRNP as a Study Coordinator
- Under VRC Production and Regulatory Affairs, replacement of Phillip Gomez III, Ph.D. with Kimberlee Wallace, Ph.D and addition of Michelle Conan-Cibotti, Ph.D.

**The above information will be incorporated into the next version of the protocol at a later time if it is amended.**

Attachments**:**

- Updated Appendix I: Study Informed Consent Form
- Updated Appendix II: Contact Information
- Information Letter for All Study Participants
